# High-quality health systems in the Sustainable Development Goals era: time for a revolution

**DOI:** 10.1016/S2214-109X(18)30386-3

**Published:** 2018-09-05

**Authors:** Margaret E Kruk, Anna D Gage, Catherine Arsenault, Keely Jordan, Hannah H Leslie, Sanam Roder-DeWan, Olusoji Adeyi, Pierre Barker, Bernadette Daelmans, Svetlana V Doubova, Mike English, Ezequiel García Elorrio, Frederico Guanais, Oye Gureje, Lisa R Hirschhorn, Lixin Jiang, Edward Kelley, Ephrem Tekle Lemango, Jerker Liljestrand, Address Malata, Tanya Marchant, Malebona Precious Matsoso, John G Meara, Manoj Mohanan, Youssoupha Ndiaye, Ole F Norheim, K Srinath Reddy, Alexander K Rowe, Joshua A Salomon, Gagan Thapa, Nana A Y Twum-Danso, Muhammad Pate

**Affiliations:** 1 Harvard T H Chan School of Public Health, Boston, MA, USA; 2 New York University College of Global Public Health, New York, NY, USA; 3 The World Bank, Washington, DC, USA; 4 Institute for Healthcare Improvement, Cambridge, MA, USA; 5 WHO, Geneva, Switzerland; 6 Mexican Institute of Social Security, Mexico City, Mexico; 7 KEMRI—Wellcome Trust Research Programme, Nairobi, Kenya; 8 Institute for Clinical Effectiveness and Health Policy, Buenos Aires, Argentina; 9 Inter-American Development Bank, Washington, DC, USA; 10 WHO Collaborating Centre for Research and Training in Mental Health, Neuroscience, Drug and Alcohol Abuse, University of Ibadan, Ibadan, Nigeria; 11 Northwestern University Feinberg School of Medicine, Chicago, IL, USA; 12 National Centre for Cardiovascular Disease, Beijing, China; 13 Federal Ministry of Health of Ethiopia, Addis Ababa, Ethiopia; 14 Bill & Melinda Gates Foundation, Seattle, WA, USA; 15 Malawi University of Science and Technology, Limbe, Malawi; 16 London School of Hygiene & Tropical Medicine, London, UK; 17 National Department of Health of the Republic of South Africa, Pretoria, South Africa; 18 Department of Global Health and Social Medicine, Harvard Medical School, Boston, MA, USA; 19 Duke University Sanford School of Public Policy, Durham, NC, USA; 20 Ministry of Health and Social Action of the Republic of Senegal, Dakar, Senegal; 21 Department of Global Public Health and Primary Care, University of Bergen, Bergen, Norway; 22 Public Health Foundation of India, New Delhi, India; 23 Malaria Branch, Division of Parasitic Diseases and Malaria, Center for Global Health, US Centers for Disease Control and Prevention, Atlanta, GA, USA; 24 Stanford Medical School, Stanford, CA, USA; 25 Legislature Parliament of Nepal, Kathmandu, Nepal; 26 MAZA, Accra, Ghana; 27 and Big Win Philanthropy, London, UK

## Executive summary

Although health outcomes have improved in low-income and middle-income countries (LMICs) in the past several decades, a new reality is at hand. Changing health needs, growing public expectations, and ambitious new health goals are raising the bar for health systems to produce better health outcomes and greater social value. But staying on current trajectory will not suffice to meet these demands. What is needed are high-quality health systems that optimise health care in each given context by consistently delivering care that improves or maintains health, by being valued and trusted by all people, and by responding to changing population needs. Quality should not be the purview of the elite or an aspiration for some distant future; it should be the DNA of all health systems. Furthermore, the human right to health is meaningless without good quality care because health systems cannot improve health without it.

We propose that health systems be judged primarily on their impacts, including better health and its equitable distribution; on the confidence of people in their health system; and on their economic benefit, and processes of care, consisting of competent care and positive user experience. The foundations of high-quality health systems include the population and their health needs and expectations, governance of the health sector and partnerships across sectors, platforms for care delivery, workforce numbers and skills, and tools and resources, from medicines to data. In addition to strong foundations, health systems need to develop the capacity to measure and use data to learn. High-quality health systems should be informed by four values: they are for people, and they are equitable, resilient, and efficient.

For this Commission, we examined the literature, analysed surveys, and did qualitative and quantitative research to evaluate the quality of care available to people in LMICs across a range of health needs included in the Sustainable Development Goals (SDGs). We explored the ethical dimensions of high-quality care in resource-constrained settings and reviewed available measures and improvement approaches. We reached five conclusions:

### The care that people receive is often inadequate, and poor-quality care is common across conditions and countries, with the most vulnerable populations faring the worst

Data from a range of countries and conditions show systematic deficits in quality of care. In LMICs, mothers and children receive less than half of recommended clinical actions in a typical preventive or curative visit, less than half of suspected cases of tuberculosis are correctly managed, and fewer than one in ten people diagnosed with major depressive disorder receive minimally adequate treatment. Diagnoses are frequently incorrect for serious conditions, such as pneumonia, myocardial infarction, and newborn asphyxia. Care can be too slow for conditions that require timely action, reducing chances of survival. At the system level, we found major gaps in safety, prevention, integration, and continuity, reflected by poor patient retention and insufficient coordination across platforms of care. One in three people across LMICs cited negative experiences with their health system in the areas of attention, respect, communication, and length of visit (visits of 5 min are common); on the extreme end of these experiences were disrespectful treatment and abuse. Quality of care is worst for vulnerable groups, including the poor, the less educated, adolescents, those with stigmatised conditions, and those at the edges of health systems, such as people in prisons.

Universal health coverage (UHC) can be a starting point for improving the quality of health systems. Improving quality should be a core component of UHC initiatives, alongside expanding coverage and financial protection. Governments should start by establishing a national quality guarantee for health services, specifying the level of competence and user experience that people can expect. To ensure that all people will benefit from improved services, expansion should prioritise the poor and their health needs from the start. Progress on UHC should be measured through effective (quality-corrected) coverage.

### High-quality health systems could save over 8 million lives each year in LMICs

More than 8 million people per year in LMICs die from conditions that should be treatable by the health system. In 2015 alone, these deaths resulted in US$6 trillion in economic losses. Poor-quality care is now a bigger barrier to reducing mortality than insufficient access. 60% of deaths from conditions amenable to health care are due to poor-quality care, whereas the remaining deaths result from non-utilisation of the health system. High-quality health systems could prevent 2·5 million deaths from cardiovascular disease, 1 million newborn deaths, 900 000 deaths from tuberculosis, and half of all maternal deaths each year. Quality of care will become an even larger driver of population health as utilisation of health systems increases and as the burden of disease shifts to more complex conditions. The high mortality rates in LMICs for treatable causes, such as injuries and surgical conditions, maternal and newborn complications, cardiovascular disease, and vaccine preventable diseases, illustrate the breadth and depth of the healthcare quality challenge. Poor-quality care can lead to other adverse outcomes, including unnecessary health-related suffering, persistent symptoms, loss of function, and a lack of trust and confidence in health systems. Waste of resources and catastrophic expenditures are economic side effects of poor-quality health systems. As a result of this, only one-quarter of people in LMICs believe that their health systems work well.

### Health systems should measure and report what matters most to people, such as competent care, user experience, health outcomes, and confidence in the system

Measurement is key to accountability and improvement, but available measures do not capture many of the processes and outcomes that matter most to people. At the same time, data systems generate many metrics that produce inadequate insight at a substantial cost in funds and health workers’ time. For example, although inputs such as medicines and equipment are commonly counted in surveys, these are weakly related to the quality of care that people receive. Indicators such as proportion of births with skilled attendants do not reflect quality of childbirth care and might lead to false complacency about progress in maternal and newborn health.

This Commission calls for fewer, but better, measures of health system quality to be generated and used at national and subnational levels. Countries should report health system performance to the public annually by use of a dashboard of key metrics (eg, health outcomes, people’s confidence in the system, system competence, and user experience) along with measures of financial protection and equity. Robust vital registries and trustworthy routine health information systems are prerequisites for good performance assessment. Countries need agile new surveys and real-time measures of health facilities and populations that reflect the health systems of today and not those of the past. To generate and interpret data, countries need to invest in national institutions and professionals with strong quantitative and analytical skills. Global development partners can support the generation and testing of public goods for health system measurement (civil and vital registries, routine data systems, and routine health system surveys) and promote national and regional institutions and the training and mentoring of scientists.

### New research is crucial for the transformation of low-quality health systems to high-quality ones

Data on care quality in LMICs do not reflect the current disease burden. In many of these countries, we know little about quality of care for respiratory diseases, cancer, mental health, injuries, and surgery, as well as the care of adolescents and elderly people. There are vast blind spots in areas such as user experience, system competence, confidence in the system, and the wellbeing of people, including patient-reported outcomes. Measuring the quality of the health system as a whole and across the care continuum is essential, but not done. Filling in these gaps will require not only better routine health information systems for monitoring, but also new research, as proposed in the research agenda of this Commission. For example, research will be needed to rigorously evaluate the effects and costs of recommended improvement approaches on health, patient experience, and financial protection. Implementation science studies can help discern the contextual factors that promote or hinder reform. New data collection and research should be explicitly designed to build national and regional research capacity.

### Improving quality of care will require system-wide action

To address the scale and range of quality deficits we documented in this Commission, reforming the foundations of the health system is required. Because health systems are complex adaptive systems that function at multiple interconnected levels, fixes at the micro-level (ie, health-care provider or clinic) alone are unlikely to alter the underlying performance of the whole system. However, we found that interventions aimed at changing provider behaviour dominate the improvement field, even though many of these interventions have a modest effect on provider performance and are difficult to scale and sustain over time. Achieving high-quality health systems requires expanding the space for improvement to structural reforms that act on the foundations of the system.

This Commission endorses four universal actions to raise quality across the health system. First, health system leaders need to govern for quality by adopting a shared vision of quality care, a clear quality strategy, strong regulation, and continuous learning. Ministries of health cannot accomplish this alone and need to partner with the private sector, civil society, and sectors outside of health care, such as education, infrastructure, communication, and transport. Second, countries should redesign service delivery to maximise health outcomes rather than geographical access to services alone. Primary care could tackle a greater range of low-acuity conditions, whereas hospitals or specialised health centres should provide care for conditions, such as births, that need advanced clinical expertise or have the risk of unexpected complications. Third, countries should transform the health workforce by adopting competency-based clinical education, introducing training in ethics and respectful care, and better supporting and respecting all workers to deliver the best care possible. Fourth, governments and civil society should ignite demand for quality in the population to empower people to hold systems accountable and actively seek high-quality care. Additional targeted actions in areas such as health financing, management, district-level learning, and others can complement these efforts. What works in one setting might not work elsewhere, and improvement efforts should be adapted for local context and monitored. Funders should align their support with system-wide strategies rather than contribute to the proliferation of micro-level efforts.

In this Commission, we assert that providing health services without guaranteeing a minimum level of quality is ineffective, wasteful, and unethical. Moving to a high-quality health system—one that improves health and generates confidence and economic benefits—is primarily a political, not technical, decision. National governments need to invest in high-quality health systems for their own people and make such systems accountable to people through legislation, education about rights, regulation, transparency, and greater public participation. Countries will know that they are on the way towards a high-quality, accountable health system when health workers and policymakers choose to receive health care in their own public institutions.

## Introduction

The past 20 years have been called a golden age for global health.^1^ Fuelled by a major increase in domestic health spending and donor funding, LMICs have vastly expanded access to health determinants (eg, clean water and sanitation) and health services alike (eg, vaccination, antenatal care, and HIV treatment).^2–4^ These expansions have saved the lives of millions of children, men, and women, largely by averting deaths from infectious diseases.^5^ However, these past decades were not as favourable for preventing deaths from non-communicable diseases and acute conditions, such as ischaemic heart disease, stroke, diabetes, neonatal mortality, and injuries, for which mortality stagnated or increased.^6^ The lowest-income countries and the poorest people within countries generally had the worst outcomes, despite considerable efforts to increase use of health care.^7^ The strategy that brought big wins for child health and infectious diseases will not suffice to reach the health-related SDGs. The newly ascendant health conditions, including chronic and complex conditions, require more than a single visit or standardised pill pack; they require highly skilled, longitudinal, and integrated care. Such care is also needed to address the substantial residual mortality from maternal and child conditions and infectious diseases. In short, it is becoming clear that access to health care is not enough, and that good quality of care is needed to improve outcomes. India learned this with Janani Suraksha Yojana, a cash incentive programme for facility births, which massively increased facility delivery but did not measurably reduce maternal or newborn mortality.^8^

High-quality care involves thorough assessment, detection of asymptomatic and co-existing conditions, accurate diagnosis, appropriate and timely treatment, referral when needed for hospital care and surgery, and the ability to follow the patient and adjust the treatment course as needed.

Health systems should also take into account the needs, experiences, and preferences of people and their right to be treated with respect.^9^ Although many consumer services make user experience a central mission, health systems—like other public sector systems—are often difficult to use, indifferent to the time and preferences of people, and reluctant to share decision-making processes.^10^ Indeed, some providers are rude and even abusive—a fundamental abrogation of human rights and health system obligations.^9^ At the same time, health workers might not receive the support and respect required to have a fulfilling professional life. Finally, systems can be inefficient, wasting scarce resources on unnecessary care and on low-quality clinics that people bypass, while imposing high costs on users.^11^

The SDG era demands new ways of thinking about health systems. Although they are only one contributor to good health—other major contributors being social determinants of health such as education, wealth, employment, and social protections, and cross-sectoral public health actions such as tobacco taxation and improved food, water, and road and occupational safety regulations^12^—access to high-quality health care is a human right and moral imperative for every country.^13^ Moreover, health systems are a powerful engine for improving survival and wellbeing and they are the focus of our report.^14,15^ We endorse WHO’s definition of a health system as consisting of “all organisations, people, and actions whose primary intent is to promote, restore, or maintain health”, and we focus this Commission on the organised health sector, public and private, including community health workers.^16^ Although informal providers (those with little or no formal clinical training) also provide care in some countries, there are—with a few notable exceptions—insufficient data on the quality of care offered by these providers, and we do not cover them in this Commission.

Addressing quality of care is particularly pertinent as countries begin to implement UHC.^17^ UHC represents a substantial new investment of national resources—one that embodies new concrete commitments about the type of care that people have a right to expect. Newly transparent benefit packages can, in turn, create public expectations that governments will be under pressure to fulfil. Furthermore, new investments in health care will face scrutiny from finance ministers, who will demand efficient use of resources and better results measured in longer lifespans, restored physical and mental functions, user satisfaction, and economic productivity.

What should a high-quality health system look like in countries with resource constraints and competing health priorities that aspire to reach the SDGs? *The Lancet Global Health* Commission on High-Quality Health Systems in the SDG Era, comprised of 30 academics, policy makers, and health system experts from 18 countries, seeks to answer this question.^18^ In this Commission, we propose new ways to define, measure, and improve the performance of health systems. We review evidence of past approaches and look for strategies that can change the trajectory of health systems in LMICs.

Our work is informed by several principles. First, the principle that health systems are for people. Health systems need to work with people not only to improve health outcomes, but also to generate non-health-related value, such as trust and economic benefit for all people, including the poor and vulnerable. Second, the principle that people should be able to receive good quality, respectful care for any health concern that can be tackled within their country’s resource capacity. Third, the principle that high-quality care should be the raison d’être of the health system, rather than a peripheral activity in ministries of health. Finally, the principle that fundamental change should be prioritised over piecemeal approaches. We recognise that health systems are complex adaptive systems that resist change and can be impervious to isolated interventions; indeed, multiple small-scale efforts can be deleterious. Quality of care is an emergent property that requires shared aims among all health system actors, favourable health system foundations, and is honed through iterative efforts to improve and learn from successes and failures. These considerations guided our analysis.

We are also aware of other major efforts on quality of care at the time of the writing of this Commission. WHO convened the Quality of Care Network to facilitate joint learning, accelerate scale-up of quality maternal, newborn, and child services, and strengthen the evidence for cost-effective approaches. WHO, the World Bank, and the Organisation for Economic Co-operation and Development (OECD) published a global report on quality of health care earlier in 2018.^19^ The US National Academy of Medicine has begun a study on improving the quality of health care across the globe.

There is also new interest in stronger primary care that can promote health, prevent illness, identify the sick from the healthy, and efficiently manage the needs of those with chronic disease.^20^ The Primary Health Care Performance Initiative, a multistakeholder effort, is focusing on measuring and comparing the functioning of primary health-care systems and identifying pathways for improvement.^21^ Primary care has been a main platform for provision of health care in low-income countries, but there—as elsewhere—the changing disease burden, urbanisation, and rising demand for advanced services and excellent user experience are challenging this current model of care.

Our work was substantially strengthened with input from nine National High-Quality Health Systems Commissions that were formed to explore quality of care in their local contexts alongside the global Commission. To ensure that our work reflects the needs of people and communities, we have sought input from a people’s voice advisory board and we obtained advice and policy perspectives from an external advisory council. Our intended audiences for the report are people, national leaders, health and finance ministers, policy makers, managers, providers, global partners (bilateral and multilateral institutions and foundations), advocates, civil society, and academics.

This report is arranged in the following manner: in section 1, we propose a new definition for high-quality health systems; in section 2, we describe the state of health system quality in LMICs, bringing together multiple national and cross-national data on quality of care for the first time; in section 3, we tackle the ethics of good quality of care and propose mechanisms for ensuring that the poor and vulnerable benefit from improvement; in section 4, we review the current status of quality measurements and propose how to measure better and more efficiently; in section 5, we reassess the available options for improvement and recommend new structural solutions; in section 6, we conclude with a summary of our key messages, our recommendations, and a research agenda.

We recognise that the level of ambition implied in our recommendations might be daunting to low-income countries that are struggling to put in place the basics of health care. In this Commission, we are describing a new aspiration for health systems that can guide policies and investments now. Regardless of starting point, every country has opportunities to get started on the path to high-quality health systems.

## Section 1: Redefining high-quality health systems

The systematic examination of health-care quality began with the work of Avedis Donabedian, whose 1966 article^22^ proposed a framework for quality of care assessment that described quality along the dimensions of structure, process, and outcomes of care. At the turn of the 21st century, the Committee on Quality of Health Care in America of the Institute of Medicine (IOM) produced two influential quality reports^23,24^ that galvanised the examination of quality in the US health system and prompted similar investigations in other industrialised countries. The IOM Committee defined quality of care as “the degree to which health services for individuals and populations increase the likelihood of desired health outcomes and are consistent with current professional knowledge”.^23^ The committee noted that 21st century health systems should seek to improve performance on six dimensions of quality of care: safety, effectiveness, patient-centredness, timeliness, efficiency, and equity. The committee also observed that “the current care systems cannot do the job. Trying harder will not work. Changing systems of care will.”^23^ In 2010, Michael Porter proposed^25^ that health systems be fundamentally accountable for producing value, which should be defined around the user. International organisations, such as WHO, and many low-income and high-income countries have relied on the IOM definition of quality and its core dimensions. WHO has also separately defined integrated people-centred health systems as systems where “all people have equal access to quality health services that are coproduced in a way that meets their life course needs”.^26^

Building on this and other work, this section sets out our rationale for an updated definition of high-quality health systems and a conceptual framework ready for the health challenges, patient expectations, and rising ambitions of today.^27,28^

The improvement of health outcomes is the sine qua non of health systems; these outcomes include longer lives, better quality of life, and improved capacity to function. In addition to better health, people derive security and confidence from having a trusted source of care when illness renders them most vulnerable. In this way, health systems also function as key social institutions, both deriving from and shaping social norms and able to promote or corrode public trust.^29,30^ Finally, health systems cannot be static and must adapt to changing societal needs. This Commission defines a high-quality health system as the following:

A high-quality health system is one that optimises health care in a given context by consistently delivering care that improves or maintains health outcomes, by being valued and trusted by all people, and by responding to changing population needs.

Context is paramount in this definition; health systems have been shaped by different histories and, as a result, function differently across LMICs.

High-quality health systems are underpinned by four values: high-quality health systems are for people and are equitable, resilient, and efficient. A focus on people begins with the self-evident observation that health systems must reach people—access is a prerequisite for benefiting from health care. However, this focus also signifies that people are not just beneficiaries of health services, but have a right to health care and have agency over their health and health-care decisions. Therefore, people become accountability agents, able to hold health system actors to account. The emphasis on people-centredness is especially crucial in health care because of the asymmetry of power and information between provider and patient. The focus on people works not only as a moral imperative to protect against the adverse effects of this power imbalance, but also as a corrective action that reduces the imbalance through patient empowerment and better accountability. Health systems must also treat well the people that work within them, who deserve a supportive work environment (safe working conditions, efficient and supportive management, and appropriate role assignment) and are themselves health-care users. Demotivated providers cannot contribute to a high-quality health system.

A focus on equity means that high-quality health care needs to be available and affordable for all people, regardless of underlying social disadvantages. Measures of quality need to be disaggregated by stratifiers of social power—such as wealth, gender, or ethnicity—and quality improvements should explicitly include poor and vulnerable people to redress existing inequities.

Health systems in LMICs have been slow to change from their legacy functions focused on infectious diseases and maternal and child health, but health needs and expectations are shifting, sometimes quickly. Health crises, such as the Ebola epidemic, acutely illustrate the need for resilient systems, defined as systems that can prepare for and effectively respond to crises while maintaining core functions and reorganising if needed.^31^ High-quality health systems also need everyday resilience to respond to routine challenges, and this requires accountable leaders who respect and motivate their front-line staff.^32^

Lastly, health systems must be efficient: although spending on health systems is tightly associated with income and therefore varies greatly across LMICs, all health systems should aim to avoid waste and achieve the maximum possible improvement in health outcomes with the investment received.

**Figure 1:**
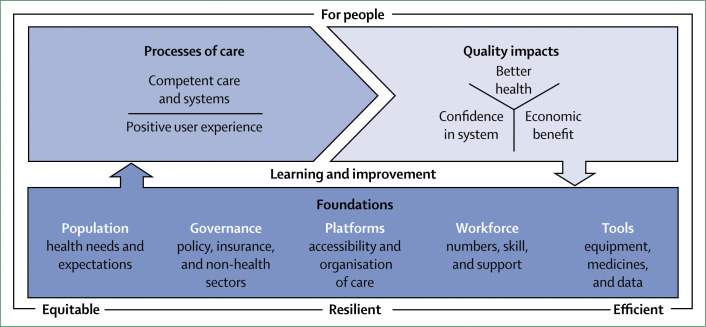
High-quality health system framework

We propose a new conceptual framework for high-quality health systems with three key domains: foundations, processes of care, and quality impacts (figure 1). This framework stems from our definition of high-quality health systems and is informed by past frameworks in the fields of health systems and quality improvement, including Donabedian’s framework,^22^ WHO’s building blocks^16^ and maternal quality of care^27^ frameworks, Judith Bruce’s family planning quality framework,^28^ Getting Health Reform Right,^33^ the Juran trilogy, and the Deming quality cycle.^34^

Our high-quality health system framework focuses on health system function, user experience, and how people benefit from health care. This Commission believes that the quality of health systems should be primarily measured by these processes and impacts rather than by inputs. Facilities staffed by health workers and equipped with running water, electricity, and medicines are essential for good quality care, but the presence of these inputs is not itself a measure of high-quality care. Empirical work shows that the quantity of such inputs does not predict the care that people receive and whether their health will improve—poor care often happens in the presence of adequate tools.^35^

Table 1 summarises the components of the three framework domains (quality impacts, processes of care, and foundations). The quality impacts begin with better health, including reduced mortality and morbidity, and positive health markers such as quality of life, function and wellbeing, and absence of serious health-related suffering.^36^ These health outcomes should also encompass patient-reported measures. Another impact of high-quality health systems is confidence in the system, including trust in health workers and appropriate care uptake. Confidence goes beyond the more traditional measure of satisfaction with care; it is the extent to which people trust and are willing to use health care. Trust is essential for maximising outcomes because it can motivate active participation in care—ie, adherence to recommendations and uptake of services, including in emergencies.^37^ Trust is also essential for the success of UHC, because financing for UHC will be primarily domestic and people are unlikely to agree to contribute taxes or pay premiums for health services that they do not value. Finally, although good quality of care might require additional investment in many health systems of LMICs, high-quality health systems have the potential to generate economic benefits. First, by reducing premature mortality and improving people’s health, ability to work, and ability to attend school, high-quality health systems can foster economic productivity. Second, high-quality health systems can reduce waste from unnecessary, ineffective, and harmful care and prevent inappropriate hospital admissions and the bypassing of cost-effective options, such as primary care. Additionally, high-quality health systems with appropriate financing mechanisms, particularly mandatory insurance, can reduce the incidence of catastrophic or impoverishing health expenditures. Therefore, financing that provides people with financial protection and promotes high-quality, efficient care is an integral foundation of a high-quality health system.

**Table 1:** High-quality health system framework components

	Components
**Quality impacts**	
Better health	Level and distribution of patient-reported outcomes: function, symptoms, pain, wellbeing, quality of life, and avoiding serious health-related suffering
Confidence in system	Satisfaction, recommendation, trust, and care uptake and retention
Economic benefit	Ability to work or attend school, economic growth, reduction in health system waste, and financial risk protection
**Processes of care**	
Competent care and systems	Evidence-based, effective care: systematic assessment, correct diagnosis, appropriate treatment, counselling, and referral; capable systems: safety, prevention and detection, continuity and integration, timely action, and population health management
Positive user experience	Respect: dignity, privacy, non-discrimination, autonomy, confidentiality, and clear communication; user focus: choice of provider, short wait times, patient voice and values, affordability, and ease of use
**Foundations**	
Population	Individuals, families, and communities as citizens, producers of better health outcomes, and system users: health needs, knowledge, health literacy, preferences, and cultural norms
Governance	Leadership: political commitment, change management; policies: regulations, standards, norms, and policies for the public and private sector, institutions for accountability, supportive behavioural architecture, and public health functions; financing: funding, fund pooling, insurance and purchasing, provider contracting and payment; learning and improvement: institutions for evaluation, measurement, and improvement, learning communities, and trustworthy data; intersectoral: roads, transport, water and sanitation, electric grid, and higher education
Platforms	Assets: number and distribution of facilities, public and private mix, service mix, and geographic access to facilities; care organisation: roles and organisation of community care, primary care, secondary and tertiary care, and engagement of private providers; connective systems: emergency medical services, referral systems, and facility community outreach
Workforce	Health workers, laboratory workers, planners, managers: number and distribution, skills and skill mix, training in ethics and people-centred care, supportive environment, education, team work, and retention
Tools	Hardware: equipment, supplies, medicines, and information systems; software: culture of quality, use of data, supervision, and feedback

The processes of care include competent care and user experience, which we consider to be complementary elements of quality. These elements must be present in both the health system as a whole and in individual care visits. Competent systems provide people and communities with health promotion and prevention when healthy and effective and timely care when sick. People should be able to count on their conditions being detected and managed in an integrated manner. Systems should also be user-focused: easy to navigate, with short wait times and attention to people’s values and preferences—this is the definition of people-centredness. When people visit providers, they should expect to receive evidence-based care, including careful assessment, correct diagnosis, and appropriate treatment and counselling. And providers should treat all people with dignity, communicate clearly, and provide autonomy and confidentiality. Disrespectful and discriminatory behaviours are crucial quality failures, as are work environments that demean or disempower providers.

The foundations of high-quality health systems begin with the populations that they serve: individuals, families, and communities. People are necessary partners in providing health care and improving health outcomes; they are not only the core beneficiaries of the health system, but also the agents who can hold these systems to account. The health needs, knowledge, and preferences of people should shape the health system response. High-quality health systems require strong governance, and financing, to promote the desired outcomes and policies to regulate providers, organise care, and institutionalise accountability to citizens. However, regulation will not be enough; health system leaders will need to inspire and sustain the values of professionalism and excellence that underpin high-quality health care. In most countries, health care is provided by three platforms: community health, primary care, and hospital care. An appropriate facility and provider mix, quality-centred service delivery models, and functioning connections between levels of care (eg, referral, prehospital transport) will be required to ensure that the whole system maximises outcomes and the efficient use of resources. Providers, from health workers to managers, are fundamental for health systems, and require adequate numbers, preparation, professionalism, and motivation. Providers need high-quality, competency-focused clinical education, with training in ethics, and a supportive environment for achieving the desired performance. Finally, health systems require not only physical tools, such as equipment, medicines, and supplies, but also new attitudes, skills, and behaviours, including quality mindsets, supervision and feedback, and the ability and willingness to learn from data. The foundations alone will not create good care, and the system will not be able to adapt to new challenges without built-in mechanisms for learning and improvement, including having timely information on performance, assessment of new ideas, and the means to retire ineffective approaches.

This framework can be used to measure health systems over time on elements that matter to people (through processes and impacts) and to guide opportunities for improvement (through shoring up or rethinking foundations).

## Section 2: What quality of care are people receiving in LMICs today?

In this section, we describe the current state of healthcare quality in LMICs. We compiled data from multiple sources to present the most comprehensive and detailed picture of health system quality. We analysed data from health facility, household, telephone, and internet surveys collected in the past 10 years, and summarised findings from global estimates, systematic reviews, and individual studies (data sources are listed in appendix 1 and a comparison of methods used to collect the data can be found in appendix 2). Within the constraints of the available data, we describe quality across all health conditions addressed by the SDGs (list of conditions in appendix 1) and across health system platforms (community outreach, primary and hospital care, and the linkages between them: referral systems and emergency medical services).

Following the Commission’s framework, we describe the current situation with regard to provision of evidence-based care, competent health systems, and user experience and we present available evidence on the links between quality and health, confidence, and economic benefits. Our focus is on describing the processes of care and their impacts. Foundations—the facilities, people, and tools required for care—are crucial to high-quality health systems, but their availability does not guarantee quality care. Lastly, we explore why some population groups are more vulnerable to poor-quality care. Where multicountry medians are presented throughout the section, country-specific data are included in appendix 2. Key findings are shown in panel 1.

*Panel 1:* Section 2 key findings

1. Poor-quality health systems result in more than 8 million deaths per year in LMICs, leading to economic welfare losses of $6 trillion.
2. Health providers in low-income and middle-income countries (LMICs) often do less than half of recommended evidence-based care actions. For example, only two in five women who delivered in a facility were examined within 1 h after birth.
3. Approximately one third of patients experience disrespectful care, short consultations, poor communication, or long wait times.
4. Inadequate integration across platforms and weak referral systems undermine the ability of health systems to care for complex and emerging conditions.
5. Less than one quarter of people in LMICs believe that their health system works well, compared with half of people in high-income countries.
6. Clinics and providers with good performance can be found in every country and studying them can inform country-wide efforts for improvement.
7. High-quality health care is inequitably distributed in many countries, with poor and vulnerable groups having worse quality care—both in terms of competent care and user experience.
8. People can be especially vulnerable to poor-quality care on the basis of particular settings of care, health conditions, and demographic factors.

### Processes of care

#### Evidence-based care

Evidence-based care includes systematic patient assessments, accurate diagnoses, provision of appropriate treatments, and proper patient counselling. In this section, we assess how these aspects are being followed, across selected SDG conditions.

**Figure 2:**
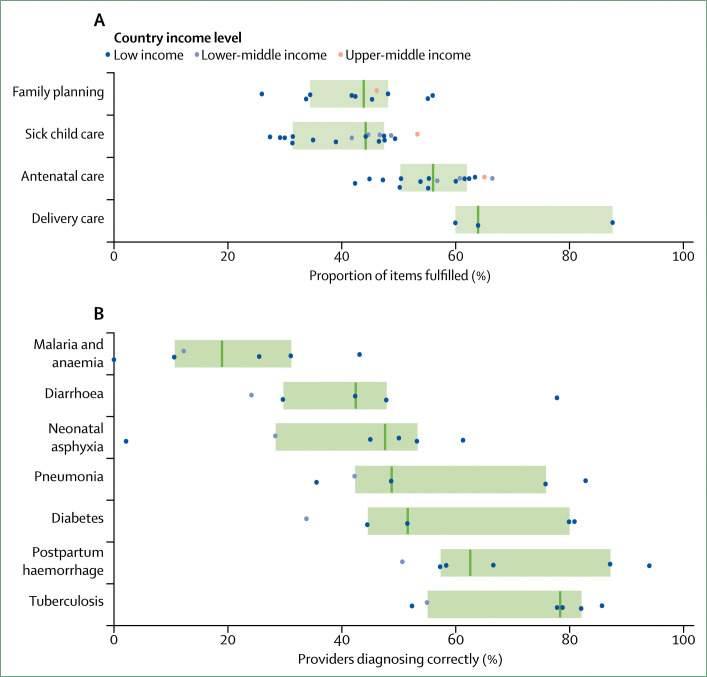
Adherence to evidence-based guidelines and diagnostic accuracy Dots represent country-specific means, vertical bars indicate median performance across countries, and boxes delineate the IQR. Indicator definitions are shown in appendix 1, and country specific means are shown in appendix 2. (A) Data are from Service Provision Assessment (SPA) surveys done in ten countries (Ethiopia 2014, Haiti 2013, Kenya 2010, Malawi 2013, Namibia 2009, Nepal 2015, Rwanda 2007, Senegal 2015–16, Tanzania 2015, and Uganda 2007) and baseline facility surveys of Results-based Financing impact evaluations (RBF) in eight countries (Burkina Faso 2013, Central African Republic 2012, Cameroon 2011, Republic of the Congo 2014, Democratic Republic of the Congo 2015, Kyrgyzstan 2012–13, Nigeria 2013, and Tajikistan 2014–15). (B) Data are from clinical vignettes from the Service Delivery Indicators surveys done by the World Bank, in cooperation with the African Economic Research Consortium and the African Development Bank in Kenya (2012), Nigeria (2013), Tanzania (2014), Togo (2013), and Uganda (2013) and from the Service Provision Assessment survey in Ethiopia (2014).

Data from direct observations of clinical consultations allowed us to measure the quality of reproductive, maternal, and child health services. Using guidelines from WHO, we identified essential elements of reproductive, maternal, and child health care and built quality indices (appendix 1). On the basis of these indices, data from observations of 81856 consultations in 18 countries showed that adherence to evidence-based guidelines is low (figure 2A). On average, providers fulfilled only 47% of recommended care—with median performance ranging from 44% for family planning consultations to 64% for labour and delivery care (appendix 2). However, median figures can mask important variations within countries (appendix 2). These large variations in performance across providers suggest that better quality of care is possible in these countries. Identifying and replicating local best practices might be valuable to inform improvement strategies.^38^

Other studies have also shown that providers often fail to adhere to clinical guidelines. In Uttar Pradesh, India, facility-based birth attendants did only 40% of items on the WHO safe childbirth checklist in a typical birth.^39^ Across 12 countries, only 50% of diarrhoea cases were correctly managed in health-care facilities according to WHO and UNICEF recommendations.^40^ In standardised patient studies in China^41^ and Kenya,^42^ only 13–45% of suspected tuberculosis cases were correctly managed by primary care providers according to the International Standards for Tuberculosis Care guidelines.

A systematic patient assessment involves gathering clinically relevant information by asking appropriate medical history questions and doing recommended examinations and tests. Data from LMICs showed that systematic patient assessments are not always done. For example, after giving birth, women should be assessed for abnormal bleeding, perineal tears, signs of infections, and high blood pressure.^43^ However, in many countries, few women reported receiving any postpartum check-up after giving birth in a health-care facility, including only 27% of women in Swaziland and 44% in Ethiopia, Burundi, and Rwanda (appendix 2). Similarly, during antenatal care, monitoring of blood pressure and urine and blood sample analyses are crucial to detect pre-eclampsia, nutritional deficiencies, infections, and other pregnancy risks.^44^ Across 91 countries, only 73% of women attending antenatal care with a skilled provider reported receiving these elements of care—ranging from an average of 54% in 30 low-income countries to 94% in 27 upper-middle-income countries (appendix 2).^45^ Poor availability of laboratory facilities and diagnostic equipment are also barriers to patient assessment and diagnosis, even when providers are aware of the necessary tests. For example, pathology service coverage in sub-Saharan Africa is approximately one-tenth of that in high-income countries.^46^ Even simple tests are often unavailable: studies showed that blood glucose meters and urine strips were available in only 18–61% of facilities across Mali, Mozambique, and Zambia.^47^ A study of ten countries found that only 2% of health-care facilities had the eight diagnostic tests defined as essential for basic service readiness by WHO.^48^

Incorrect diagnoses have deleterious consequences on health and contribute to treatment delays and antimicrobial resistance. For example, diagnostic uncertainty about undifferentiated fever often leads to overprescription of antimicrobial therapy.^49^ Our analyses of data from clinical vignettes done in LMICs revealed wide variations in diagnostic accuracy. In six sub-Saharan African countries, correct diagnoses ranged from 0 providers in Togo identifying malaria with anaemia to 94% of providers in Kenya diagnosing post-partum haemorrhage (figure 2B, appendix 2). Other work has shown that, across six eastern European and central Asian countries, acute myocardial infarctions were correctly diagnosed by only 33% of providers.^50^ Performance in practice is also likely to be worse than on vignettes: diagnostic accuracy ranging from only 8% to 20% has been reported for childhood pneumonia in Malawi^51^ and for a range of primary care conditions in India.^52^ Poor quality of laboratory testing and a heavy reliance on outdated diagnostic technologies can also contribute to misdiagnoses. For example, an external quality assessment^53^ in the Democratic Republic of the Congo found that only 4% of laboratories correctly identified the parasites that cause malaria and human African trypanosomiasis on all slides analysed. Similarly, studies^54^ in Latin America have reported Pap smear sensitivity as low as 20–25% and lower than expected rates of HER2 (human epidermal growth factor receptor 2) positivity in women with early breast cancer. For tuberculosis, uptake of newer diagnostics has been slow and many countries continue to rely on often inaccurate smear microscopy.^55^ In high-burden countries, nine sputum smears are done for every gold standard test (Xpert MTB/RIF) used.^55^

**Figure 3:**
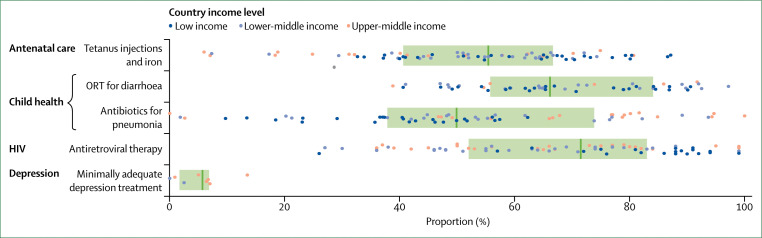
Proportion of individuals receiving appropriate treatments among those who seek care in 112 low-income and middle-income countries Dots represent country-specific means, vertical bars indicate median performance across countries, and boxes delineate the IQR. Data sources for tetanus injections and iron during antenatal care were Demographic and Health surveys (DHS) and Multiple Indicator Cluster surveys in 75 countries; for oral rehydration therapy (ORT) were DHS in 54 countries; for antibiotics for pneumonia were DHS and Multiple Indicator Cluster surveys in 63 countries; for antiretroviral therapy among those aware of their HIV status were UNAIDS estimates in 78 countries; and for minimally adequate depression treatment were World Mental Health Surveys in 8 countries. Indicators are defined in appendix 1; country specific means are shown in appendix 2.

Poor-quality care also includes the underuse^56^ of effective care and the overuse^11^ of unnecessary care. Our analyses of survey data revealed that individuals in LMICs often do not receive appropriate treatments during consultations, including preventive interventions during skilled antenatal care, oral rehydration therapy for children with diarrhoea, or antibiotics for those with symptoms of pneumonia (figure 3, appendix 2). Similarly, another study^57^ in Malawi reported that only 38·7% of patients with non-severe pneumonia confirmed on re-examination were correctly prescribed first-line antibiotics during consultation. Additionally, despite being diagnosed, many patients are untreated or undertreated for conditions such as HIV, tuberculosis, hypertension, diabetes, and depression.^58–63^ In LMICs where data are available, only 68% of people aware of their HIV status are on antiretroviral therapy, and only 5% of people with a diagnosis of major depressive disorder receive minimally adequate treatment (figure 3, appendix 2). Individuals in severe pain are also systematically undertreated in LMICs.^36^ Of the 298·5 metric tonnes of morphine-equivalent opioids distributed in the world every year, only 0·03% of that is distributed in low-income countries, leading to a 98% unmet need for morphine.^36^ A study^64^ showed that, among patients with ST-segment elevation myocardial infarctions admitted to Chinese hospitals, only half of ideal candidates for reperfusion therapy received the treatment. Other treatments that reduce mortality in patients were also underused, with only 58% of eligible patients receiving β blockers and 66% receiving angiotensin-converting-enzyme inhibitors.^64^ All these reports represent major missed opportunities to improve outcomes among people already using the health system.

Overuse of unnecessary or ineffective care has also been documented in LMICs. In the previously mentioned study^64^ in China, almost a third of patients received magnesium sulphate—a treatment that is ineffective—on admission and more than half of patients were given traditional Chinese medicine, despite little evidence of its efficacy and safety.^64^ Other instances of inappropriate care in LMICs include unnecessary use of antibiotics for diarrhoea, inappropriate cardiac interventions, overuse of steroids, and unnecessary hysterectomies.^11,65,66^ Although many women still do not have access to needed caesarean sections, rates of unnecessary caesarean sections have been increasing in LMICs.^11,67^ Inappropriate use and overprescription of antimicrobials, combined with poor sanitation, inadequate access to diagnostic tools, and low diagnostic accuracy, have fuelled antimicrobial resistance throughout LMICs.^68^ A 2018 study^69^ assessed the quality of antimicrobial prescribing for hospital inpatients in 53 countries, including 25 LMICs. Inappropriate antibiotic prescribing practices included prescriptions for unknown diagnoses, prescriptions without stop or review dates (to avoid unnecessarily long antibiotic courses), and prolonged surgical prophylaxis.

Proper counselling and health education are essential elements of evidence-based care. We found that during antenatal care, many skilled providers do not advise women on the signs of pregnancy complications or how to prevent HIV infections, and, when prescribing contraceptives, many providers fail to discuss their potential side-effects (appendix 2). Similarly, providers often do not state their diagnosis during the consultation.^52^ In observations of sick child consultations in 17 countries, only 43% of providers informed caregivers about the diagnosis of their child (appendix 2). Counselling is particularly important for chronic disease management. Tobacco use, excess weight, unhealthy diets, and physical inactivity are the leading risk factors for non-communicable diseases. Data from the WHO STEPS survey in seven LMICs showed that providers did not counsel many patients diagnosed with cardiometabolic diseases: only 16% of patients were counselled on tobacco, 29% on exercise, and 55% on dietary changes (appendix 2). In six Latin American and Caribbean countries, only 56% of patients diagnosed with at least one chronic condition reported receiving advice on diet and exercise from primary care providers (appendix 2).^70^

#### Competent systems

Beyond the content of the health-care visit, competent care requires the whole health system to function for the patient. Here, we describe current evidence on four elements of competent health systems: safety, prevention and detection, continuity and integration, and timely care.

The literature documents a range of safety problems in health care, including adverse drug events, adverse events and injuries due to medical devices, injuries due to surgical and anaesthesia errors (including wrong-site surgery), health-care-associated infections, improper transfusion and injection practices, falls, burns, and pressure ulcers.^71^ Despite lower health-care use rates, LMICs bear the majority of the global burden of adverse events from unsafe care.^72^ Surgical site infections, the most common type of health-care-associated infection, are markedly higher in LMICs than in high-income countries.^73^ Patient safety literature has been largely focused on inpatient care, but adverse events also occur to outpatients, including medication errors, infections resulting from poor hand hygiene, unsafe injections, blood samples, or reusable equipment. LMICs are estimated to have rates of medication-related adverse events similar to those of high-income countries, but they result in twice as many years of healthy life lost because more younger patients are affected in LMICs.^72^ One study found that, across 54 LMICs, 35% of healthcare facilities do not have water and soap for handwashing and 19% do not have improved sanitation.^74^ This absence of services compromises efforts to improve hygiene behaviours and reduce health-care-associated infections. However, although water and sanitation are necessary, handwashing does not necessarily associate with their presence: low adherence to hand hygiene was found even in facilities with available supplies.^75^ Beyond their costs to human lives and disability, adverse events from unsafe care are also costly in terms of loss of trust in the health system.

The prevention and early detection of diseases, including through recommended screenings, is an important function of high-quality health systems. Across six Latin American and Caribbean countries, less than half of adults reported having had their blood pressure checked in the past year and their cholesterol checked in the past 5 years.^76^ Rates of cervical and breast cancer screening also vary widely.^54^ Across six LMICs surveyed by the WHO study on global ageing and adult health (SAGE), mammogram coverage averaged 20% of all women of screening age and was as low as 1% in India and 2% in Ghana (appendix 2).^63^ Across nine countries in the Americas, average Pap smear coverage was 36% of women in need, ranging from 10% in Nicaragua to 97% in Panama.^77^ Even people in the health system might not receive the needed screening or early detection. In countries with HIV prevalence higher than 5%, WHO recommends that all pregnant women be tested for HIV.^78^ In five of nine high-prevalence countries, more than 95% of pregnant women attending antenatal care were tested for HIV. However, despite a HIV prevalence of 27% in Swaziland and 12% in Mozambique, only 56% of women in Swaziland and 69% in Mozambique are tested during antenatal care (appendix 2).

Continuity of care is reflected by the ability of the health system to retain people in care and by the patient’s ability to see a clinician familiar with their medical history. Integration is the extent to which health services are delivered in a complementary and coherent manner. These two dimensions are important for the management of non-communicable diseases and other chronic conditions, such as HIV, that require continuous patient support after diagnosis and a comprehensive treatment approach.^58^ Across services including antenatal care, child vaccination, antiretroviral therapy, and mental health care, retention rates ranged from 87% for diphtheria-tetanus-pertussis (DTP3) vaccination in 83 LMICs to only 55% retention for mental health care in 12 LMICs (appendix 2).^79,80^ Similarly, lapses in the follow-up of test results have also been reported and pose severe challenges for infectious conditions such as HIV and tuberculosis.^59,71^ A systematic review^81^ estimated patient losses to the system between diagnosis and treatment for tuberculosis to be as high as 18% in Africa and 13% in Asia. Regarding integration, all tuberculosis patients should be tested for HIV, because of risk factors shared between the two infections.^78^ In the WHO African Region, where the burden of HIV-associated tuberculosis is highest, 82% of patients with tuberculosis were tested for HIV.^82^

For people with life-threatening emergencies, such as labour complications, trauma, and stroke, treatment delays substantially increase mortality risk. Timeliness is also central for other conditions that can be cured if treated early—including many cancers—and conditions such as tuberculosis or diabetes, in which early treatment prevents transmission or disease progression. Time intervals from admission to surgery for traumatic fractures of the femur were found to be substantially longer in LMIC hospitals than in high-income country hospitals.^83^ Numerous studies have described the delays that occur during labour complications in women deciding to seek care and in reaching health facilities—the so-called first and second delays. However, the third delay—in providing high-quality care once women reach health-care facilities—is emerging as an important contributor to maternal and newborn child mortality.^84^ For example, a study^85^ in India found that attending to women within 10 min of their arrival to the facility could have prevented 37% of recorded stillbirths. Additionally, the absence of immediate postpartum care can lead to serious obstetric complications being missed. Across 41 countries with a demographic and health survey, we found that only 41% of women delivering in a health-care facility reported someone checking on their health within 1 h of delivery (appendix 2).

For infectious diseases, such as tuberculosis, making a timely diagnosis is crucial for interrupting transmission and optimising treatment outcomes. A review^86^ of studies done in LMICs found that an average of 28·4 days passed between the first contact of patients with the health system and the date of tuberculosis diagnosis, ranging from 2 days in China to 87 days in Pakistan. Regarding cancer care, delays caused by both patient and health system contribute to advanced disease at presentation and high cancer mortality rates in LMICs. Studies^54,87,88^ from Brazil, Ghana, Mexico, Peru, and Rwanda reported delays of up to 28 weeks between presentation to a doctor and definitive diagnoses of cervical or breast cancer. Data from the Mexican Institute of Social Security, the largest health system in Mexico, revealed that 51% of women with breast cancer waited more than 30 days between mammography and diagnosis, and 44% of women with cervical cancer waited more than 30 days between Pap smear and diagnosis.^89^ Delays in initiating treatments further affected the prognosis of patients. According to the Mexican Institute of Social Security, as many as 70% of women with breast cancer and 61% of women with cervical cancer waited more than 21 days between receiving the diagnosis and beginning therapy.^89^ Similarly, a study^90^ done in Buenos Aires hospitals, Argentina, found that the median time elapsed between diagnosis of breast cancer and treatment with chemotherapy was 76 days in public hospitals and 60 days in private hospitals. These delays are concerning because waiting more than 5 weeks before starting definitive treatment can worsen survival for cervical cancer, and delays in diagnosis longer than 12 weeks are considered suboptimal for breast cancer.^54,87^

#### User experience

Competent care and competent health systems are necessary for achieving high-quality care, but a positive user experience is also important. In addition to having an intrinsic value, positive user experience can improve retention in care, adherence to treatments, and, ultimately, confidence in health systems.^91^ Additionally, some studies have found that positive user experience is linked to better technical quality.^91,92^

**Figure 4:**
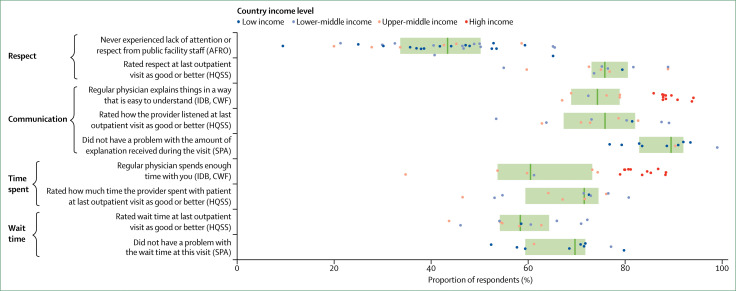
User experience in 49 low-income and middle-income countries (LMICs) and 11 high-income countries Dots represent country-specific means, vertical bars indicate median performance across countries, and boxes delineate the IQR. High-income countries do not contribute to the illustrated medians. Data are from the surveys indicated. AFRO=Afrobarometer survey done in 34 African LMICs (2011–13). HQSS=Commission-led internet survey done in 12 LMICs (2017). IDB=nationally representative phone survey on primary care access, use, and quality done by the Inter-American Development Bank in six Latin-American and Caribbean LMICs (2013). SPA=Service Provision assessment surveys done in ten LMICs (2007–16). CWF=International Health Policy Survey done by the Commonwealth Fund in 11 high-income countries (2013). Indicators are defined in appendix 1; country specific means are shown in appendix 2.

To address insufficient cross-national data on user experience, this Commission did an internet survey on user experience in 12 countries in Africa, Latin America, Asia, and the Middle East. Full results will be presented in forthcoming papers, but some of the key results of this survey are shown in figure 4, along with indicators from four other surveys done in 49 LMICs and 11 high-income countries (appendix 2).^70^ We found that an average of 34% of people in LMICs reported poor user experience, citing a lack of attention or respect from facility staff (41%), long wait times (37%), poor communication (21%), or short time spent with providers (37%). This result on the short time spent with providers was echoed by a 2017 review^93^ that found that primary care consultations lasted fewer than 5 min on average in LMICs.

*Panel 2:* Beyond the numbers—experiences in the health system*

Interviews with patients help to paint a more comprehensive picture of their experiences within the health system. The Word Bank’s landmark publication, Voices of the Poor,^A1^ in 2000 shared the narratives of individuals across the world and described the challenges that the poor face in not only accessing health care but also successfully navigating the health system. Since then, several qualitative studies have further illuminated the ways in which people receive differential treatment while seeking care. We did a rapid review of these studies (methods are described in appendix 1). The stories described in these studies highlight disparities in both competent care and user experience.

Patients across a wide range of low-income and middle-income countries have described the lack of competent care and health systems. In Egypt, a woman said that “at the hospital, they do nothing to people unless they are staff relatives, or rich people that have power or authority.”^A1^ A focus group participant in Tanzania^A2^ stated that “they are very often saying that medicines are available or not available. When someone tells you they aren’t, it’s her siri (secret). She is the only one who knows. She decides when she sees you coming. … This really upsets us…. The obstacles are like these ones of medicines even if there are no medicines what makes me feel bad is the game.” Patients also reported improper examinations and care. A focus group participant in Ethiopia^A3^ described her delivery care: “they left the placenta inside me. Because they are impatient, they did not examine me. After I gave birth, I rested there for 5 h but no one came and asked me whether I was bleeding… After 3 days, my face got swollen… I almost died.”

Studies also highlight poor user experience, including verbal abuse and neglect from health-care workers. According to a patient in Russia, “the hospital is like a prison”.^A1^ A person in Ghana^A4^ recounted that “people always say that the nurses are shouting too much, and saying bad things to them, and maybe they don’t want to treat them. They only care for those big people who have money to give them.” Poor patients, such as this respondent in Timor Leste,^A5^ also frequently report disrespectful, discriminatory treatment from health-care workers: “Health workers yell at us like a slave… they give priority to the important people, rich and intellectual and neglecting the poor, no money, stupid and dirty…That is the reason why people do not want to go to the hospital although they have a letter of referral.”

*Panel references can be found in appendix 1.

Some differences across surveys are worth noting. In Afrobarometer survey countries, 42% of respondents reported never experiencing a lack of attention or respect, whereas in the internet survey, 75% of respondents reported respectful care at their last visit. Differences in countries and income groups (our survey was done in more middle-income countries than those of Afrobarometer), wording (“never experienced” was used in Afrobarometer surveys), time frames (past year *vs* last visit), and survey sampling (internet users have a higher average socioeconomic status than household respondents) might explain these differences. Differing expectations of quality can also influence the perception of user experience.

No benchmarks exist for what constitutes good user experience. However, user ratings of communication and time spent with providers were consistently higher in high-income countries than in LMICs (figure 4), with only 11% of respondents reporting poor communication and 17% reporting insufficient time with providers (compared with 74% and 60% on average in the six Latin American and Caribbean countries surveyed by the Inter-American Development Bank).

Disrespect and abuse of women during childbirth has been widely reported in LMICs,^9^ including documented instances of physical abuse, non-consented clinical care, no confidentiality and dignity, discrimination, abandonment, and detention in facilities. A review^9^ of studies showed a range of 19–98% of women reporting mistreatment during childbirth across LMICs, with 3–36% reporting physical abuse. Beyond being an indicator of poor-quality care, disrespect and abuse should be unacceptable in any health system.

Nonetheless, these numbers can only tell part of the story. The quality of the processes of care, particularly of the user experience, is also reflected in the patient voices in panel 2.

### Quality impacts

High-quality care—both competent care and positive user experience—can have an effect on people’s health, their confidence and trust in health systems, and economic outcomes. In this section, we present available evidence on morbidity and mortality linked to poor quality care. We also synthesise data on people’s confidence in health systems, and we address the potential economic benefits of high-quality care.

#### Health

Although the causes of death are often multifactorial, and are not solely influenced by health care, deaths from some conditions are highly dependent on quality of care and are regarded as sensitive indicators of how well a health system is functioning. For this Commission, we did an analysis of the mortality burden of poor-quality care across health conditions relevant to SDGs.**^94^** We compared mortality for conditions amenable to health care between LMICs and countries with well performing health systems, to estimate the mortality that can be attributed to poor-quality health systems.

We estimated that 8·6 million deaths per year (uncertainty interval [UI] 8·5–8·8 million) in 137 LMICs are due to inadequate access to quality care. Of these, 3·6 million (UI 3·5–3·7 million) are people who did not access the health system, whereas 5·0 million (UI 4·9–5·2 million) are people who sought care but received poor-quality care. Poor-quality care resulted in 82 deaths per 100 000 people in LMICs—an annual mortality rate equivalent to that from cerebrovascular disease globally.^94^

**Figure 5:**
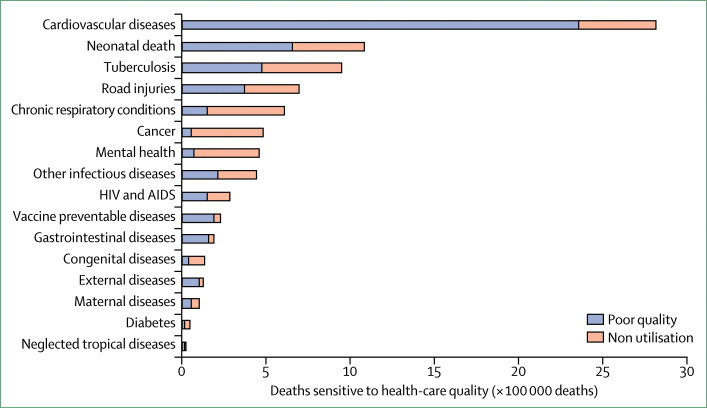
Deaths from Sustainable Development Goal conditions due to poor-quality care and non-utilisation in 137 low-income and middle-income countries External factor deaths are those due to poisonings and adverse medical events. Other infectious diseases deaths are those due to diarrhoeal diseases, intestinal infections, malaria, and upper and lower respiratory infections.

Cardiovascular deaths make up 33% of deaths amenable to health care (figure 5).^94^ Ischaemic heart disease is the largest contributor to amenable cardiovascular disease deaths, with 1·4 million deaths due to poor-quality care and 260 000 due to non-utilisation of health systems. Of the 2 million deaths from neonatal conditions and tuberculosis that are amenable to health care, 56% occurred in people who used the health system, but did not receive good quality care. Across several other health priorities for which coverage is still low, including chronic respiratory disease, cancer, mental health, and diabetes, non-utilisation of health systems plays a larger role than poor-quality care, but this will change as access increases. Our results highlight that health systems could be more effective in saving lives across a spectrum of conditions by improving quality of care along with expanding coverage. An analysis done with similar methods for a shorter list of conditions found that, globally, 8·0 million deaths could be averted with access to high-quality care.^95^

**Figure 6:**
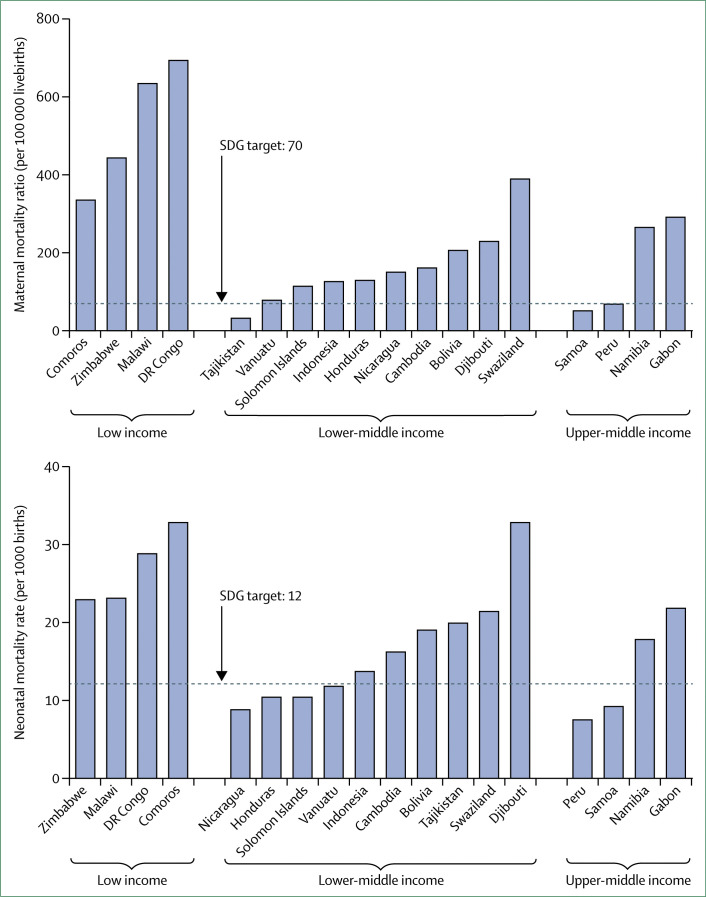
Differences in maternal and neonatal mortality rates across low-income and middle-income countries with 80–90% skilled birth attendance coverage Mortality estimates are from WHO, using 2015 modelled estimates. Skilled birth attendance is from the World Bank World Development Indicators, using the most recent data available in the past 10 years. Horizontal lines indicate Sustainable Development Goal targets. Few deaths in these countries are recorded in complete vital registration systems; global estimates must account for missing and unreliable data. Mortality estimates should be interpreted with caution because of uncertainty from measurement error. References can be found in appendix 1.

Maternal and newborn deaths are a particularly sensitive measure of health system quality, because many deaths stemming from labour complications can be averted with appropriate treatment.^96^ Figure 6 shows the comparison of rates of maternal and newborn deaths in countries with similar, high coverage of skilled attendants during birth (80–90% of births). Countries were grouped by income to reduce the influence of social and economic determinants. Across countries with similar coverage, large disparities in maternal and neonatal mortality are apparent. The ratio of worst to best performing country for maternal mortality was 2·1 in low-income, 12·2 in lower-middle-income, and 5·7 in upper-middle-income countries; for neonatal mortality it was 1·4, 3·7, and 2·9, respectively, suggesting differences in quality of care.

The frequency of stillbirths can also be reduced with high-quality care.^97^ An analysis done for this Commission—with use of the Lives Saved Tool—in 81 countries that are the focus of the Countdown to 2030 collaboration, estimated that 520 000 stillbirths could be prevented and 670 000 neonatal and 86 000 maternal lives could be saved in these countries by 2020 if adequate quality of care is provided at current levels of health system use (appendix 1). Because quality was measured by use of inputs to care rather than by processes of care, these figures might underestimate actual mortality. An older analysis that used different methods found similar effects on stillbirths, but more maternal and newborn lives saved.^98^ In addition to improving the quality of labour and delivery care, improving the quality of antenatal care and family planning is crucial to reducing stillbirths.^97^

Population-based cancer survival is also an indicator of overall health system effectiveness.^99^ Using cancer registries from 71 countries, a 2018 study^99^ found varying rates of cancer survival between countries and for different cancers. For example, most countries reported an increasing trend in 5-year net survival from breast cancer since 1995, but survival did not always increase in countries such as India, Thailand, and several eastern European countries.^99^

More broadly, hospital mortality can be useful for gauging the quality of care in facilities, when adjusted for disease severity and underlying risk, and can provide useful insight on the quality of secondary care in a region or country, when aggregated. Delivering high-quality hospital care requires well functioning facility systems that include appropriate triage in emergency departments, rapid decision making for very sick patients, close inpatient monitoring, and rigorous infection prevention practices, among other elements. Studies in LMICs have revealed high institutional maternal, perioperative, and emergency department mortality rates and high in-hospital mortality rates in patients admitted for acute myocardial infarctions. For example, the WHO multicountry survey^100^ on maternal and newborn health found intrahospital maternal mortality ratios that were 2–3 times higher than expected on the basis of case severity. High rates of perioperative and anaesthetic-related mortality were also found in LMIC hospitals, reflecting gaps in surgical and hospital care quality.^101–104^ The African surgical outcomes study^101^ found that patients in Africa were twice as likely to die after surgery compared with the global average, despite being younger, with a lower surgical risk profile, and undergoing less complex surgeries. Most of the deaths occurred post surgery, suggesting that many lives could be saved by effective surveillance for physiological deterioration in patients who have developed complications. Similarly, although the quality of emergency and trauma care in LMICs is understudied, one study found that mortality recorded in emergency departments in LMICs is many times higher than that generally reported in high-income countries, pointing to gaps in the quality and appropriateness of services being provided in these emergency departments.^105^ In patients admitted with ST-segment elevation myocardial infarction in China, in-hospital mortality did not significantly change between 2001 and 2011, suggesting a need for improvements in quality.^64^

Mortality alone does not capture the full burden of poor-quality care. People accessing poor-quality care can develop morbidities, including physical sequelae, persistent symptoms, reduced function, pain, and poor quality of life. For example, for many people in LMICs, access to health care does not result in control of manageable conditions such as hypertension, diabetes, HIV, tuberculosis, chronic lung diseases, and depression. Poor quality of care during childbirth can also result in morbidities with lifelong consequences.

A study^106^ of 1·7 million adults in China found that only 24% of patients under treatment for hypertension had achieved blood pressure control. A nationally representative study,^107^ also from China, found that among patients receiving treatment for diabetes, only 40% had achieved adequate glycaemic control. Complications of diabetes such as blindness, kidney failure, and lower limb amputation can be largely averted through high-quality primary care. However, in 2016, the Mexican Social Security Institute reported 4518 major lower limb amputations in patients with diabetes, for an incidence of 120 per 100 000 patients. This continues a previously documented trend of increasing incidence of diabetic amputations and is higher than the comparable incidence in most, but not all, OECD countries.^89,108^

According to 2017 UNAIDS estimates,^79^ only 71% of people on antiretroviral therapy in LMICs have achieved viral suppression, and only ten countries have reached the 90% viral suppression target. Tuberculosis treatment success rates are also reflective of the quality of care, and only eight of the 30 countries with high tuberculosis burden have reached 90% first-line treatment success rate.^109^ In countries with high drug-resistant tuberculosis burden, treatment success rates range between 50% and 85%.^109^ These figures show a need for better follow-up, treatment, and counselling of patients with manageable conditions in LMICs.

Obstetric fistula is a highly debilitating condition with severe social and health consequences. Women with fistula have leakage of urine or stool through the vagina and are ostracised because of this in some regions.^110^ Fistulas typically develop in women with prolonged obstructed labour. Although cultural factors, such as child marriage, increase the risk of obstructed labour, the existence of fistulas on a wide scale, as documented in studies, is an indicator of poor quality obstetric care and a broader health system failure.^111^ Using data from demographic and health surveys in 25 countries, we estimated the proportion of women who suffered from symptoms of an obstetric fistula among those whose last birth was attended by a skilled provider. In women whose last delivery was done with a skilled attendant, ten per 1000 women reported symptoms of an obstetric fistula, ranging from 0·54 per 1000 in Burkina Faso to 32 per 1000 in Pakistan (appendix 2). By contrast, obstetric fistulas have been almost eliminated in high-income countries.

Another goal of treatment is remission or reduction of symptoms. In the WHO SAGE, only 50% of patients receiving treatment for chronic lung disease and only 7% receiving treatment for depression reported having no symptoms from the two diseases in the preceding 2 weeks (appendix 2). The *Lancet* Commission^36^ on palliative care and pain relief quantified the global burden of serious health-related suffering and found that more than 80% of the global 61 million patients affected by serious health-related suffering live in LMICs.

#### Confidence in the system

The quality of care that people receive also has important consequences for their confidence and trust in their government and health system, which can affect their decisions of when and where to seek care.

**Figure 7:**
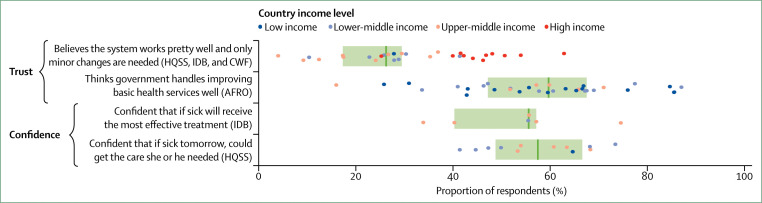
Confidence and trust in health systems in 45 low-income and middle-income countries (LMICs) and 11 high-income countries Dots represent country-specific means, vertical bars indicate median performance across countries, and boxes delineate the IQR. High-income countries do not contribute to the illustrated medians. Data are from the surveys indicated. AFRO=Afrobarometer survey done in 34 African countries (2011–13). HQSS=Commission-led internet survey done in 12 LMICs (2017). IDB=nationally representative phone survey on primary care access, use, and quality done by the Inter-American Development Bank in six Latin-American and Caribbean LMICs (2013). CWF=International Health Policy Survey done by the Commonwealth Fund in 11 high-income countries (2013). Indicators are defined in appendix 1; country specific means are shown in appendix 2.

Figure 7 shows varying degrees of confidence and trust in health systems across 45 LMICs. Only 24% of people stated that they believe that their health system worked “pretty well” and that only minor changes were necessary to make it work better.^112^ In comparison, 47% of respondents agreed with the same statement in 11 high-income countries, ranging from 24% in the USA to 61% in the UK (appendix 2).^113^ Differences in survey sampling and indicator wording might account for some of the variation across surveys. For example, increased confidence in the ability to receive the care needed present in the internet survey led by this Commission might be explained partly by a higher socioeconomic status of internet users. Gallup World polls^114^ also showed large gaps in satisfaction between low-income and high-income countries: in sub-Saharan Africa, northern Africa, and the Middle East, only 42–49% of respondents were satisfied with the availability of high-quality care near them, compared with 86% in northern Europe. Nonetheless, patient satisfaction should be interpreted with caution as a measure of quality (panel 3).

*Panel 3:* Why are people satisfied with poor quality?*

Perhaps paradoxically, because of the prevalence of poor-quality health care, patients in low-income and middle-income countries tend to report high satisfaction with the care received. Across eight low-income countries, 79% of patients and caregivers reported being very satisfied with the care received during consultations in which providers did less than half of essential clinical actions (results in appendix 2). This percentage ranged from 75% for care of sick children to 85% for family planning (appendix 2). High satisfaction with health care is common across low-income and middle-income country surveys, but patient satisfaction as a measure of quality should be carefully interpreted.

Although satisfaction is influenced by the quality of care, it is also influenced by care accessibility, costs, health status, expectations, immediate outcomes of care, and gratitude.^A9^ Additionally, satisfaction measures can be subject to substantial survey bias.^A10^ In the Commission’s internet survey of patient experience, we tested one factor thought to be influential in generating high satisfaction: low expectations for quality of care. Respondents were asked to rate the quality of care on the basis of short vignettes.

A vignette that described a nurse changing the medication of a patient with hypertension without measuring blood pressure or asking about symptoms was rated as good to excellent quality of care by an average of 53% of 17 966 respondents across 12 countries, and as high as 62% of 1292 respondents in Senegal, suggesting a low threshold for what is considered to be good care (appendix 2). Low expectations of what constitutes good quality might be a consequence of the prevailing poor-quality care, low agency, and inadequate functioning mechanisms to hold systems accountable.

Other studies have also shown that patient satisfaction surveys are influenced by acquiescence bias. Surveys framing statements in a positive way and inviting patients to agree or disagree will lead to positive responses much more frequently than surveys with more neutral statements.^A10^ More discussion on the utility of patient satisfaction as a measure of health system quality can be found in Section 4.

*Panel references can be found in appendix 1.

Other research has found that increased technical quality of health services, combined with responsive service delivery, fair treatment, better health outcomes, and financial risk protection, was associated with an increase in the probability of having trust in government.^29^ Similarly, a better user experience (communication and time spent with providers) was associated with better trust in health systems in Latin America and the Caribbean.^112^

Research suggests that quality, particularly that perceived by the patient, might have an effect on healthcare utilisation patterns, retention in care, and people’s decision to bypass facilities.^115,116^ In the internet survey led by this Commission, more than half of patients who decided not to seek care in the preceding year (despite needing medical attention) stated that their decision was made for quality reasons (eg, poor provider knowledge, long wait times, or disrespect), as opposed to cost of care or distance to facilities. The highest proportion of patients was in Mexico, where 73% cited quality reasons for not seeking care. Similarly, a study^117^ in Haiti found that higher quality primary care facilities were associated with higher utilisation.

**Figure 8:**
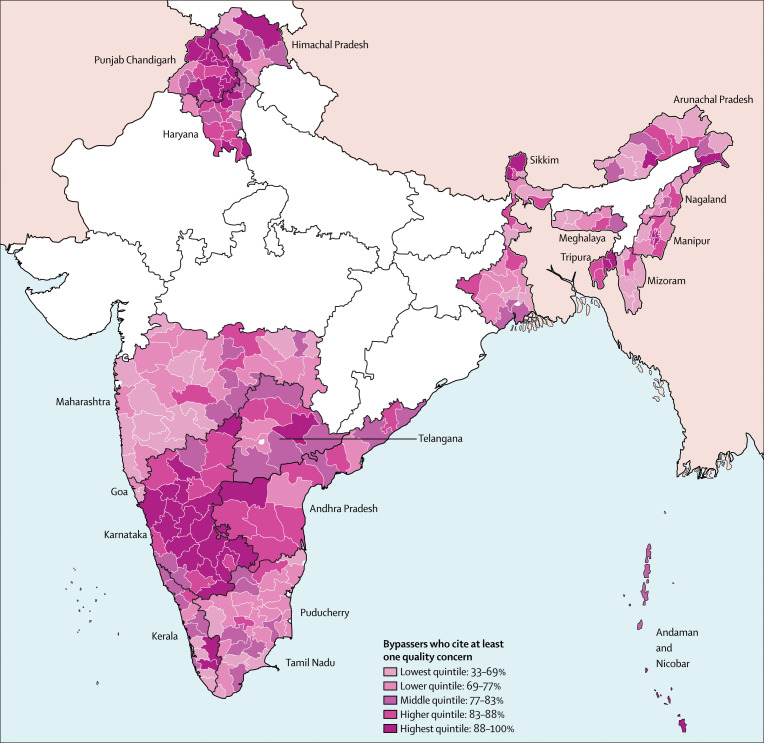
Proportion of households that report quality concerns as reason for bypassing public facilities in districts in India Data are from the fourth cycle of the District Level Household and Facility Survey done by the International Institute of Population Sciences from 2012 to 2014, in 21 states of India. A quality concern was defined as mentioning any of the following as a reason for bypassing government facilities: inadequate infrastructure, doctor not available, absent health workers, poor quality, drugs not available, inconvenient hours, long wait time, or distrust. In darker coloured districts, a higher proportion of households cited quality concerns.

Perceived poor quality of care can also lead people to bypass certain facilities. Households might choose to travel further distances or pay more out of pocket to seek better quality care.^118,119^ In India, many patients choose to seek care from the private sector, which is viewed as more competent than public facilities. India’s District Level Household and Facility Survey found that 51% of households bypassed their nearby public facility for their usual care; of these, 80% cited at least one quality concern as a reason (figure 8, appendix 1). Some people might also choose to bypass primary care facilities and seek care at hospitals or higher-level facilities for conditions that could be treated in primary care.^120^ A survey^121^ in China found that poor quality of care and lack of trust in primary care institutions were among the most common reasons for bypassing primary care and going directly to hospitals. Primary care is the cornerstone of a high-quality health system, serving as the main entry point for most concerns and playing a crucial role in coordinating care and ensuring continuity across health system platforms. Nonetheless, primary care facilities often fail to fulfil their role. Using facility surveys from nine countries, we built a primary care quality score based on three domains of quality—evidence-based care, competent systems, and user experience—and found an average score of only 0·41 out of 1, ranging from 0·32 on average in Ethiopia to 0·46 in Namibia (appendix 2). By contrast, some studies^122^ have not found a relation between utilisation and measures of quality, such as doctors’ competence, probably because of information asymmetry. A crucial area for future research will be to estimate the demand response to higher quality of care, focusing on the role of information and perception of quality in influencing utilisation patterns.

#### Economic benefit

Improving health system quality can be justified on ethical, epidemiological, and economic grounds. Little evidence exists on the link between levels of quality of care and economic outcomes. Here, we describe three types of economic consequences that could be averted by high-quality health systems: macroeconomic effects of premature mortality, health system waste, and catastrophic or impoverishing health expenditures faced by households.

A 2018 analysis^95^ estimated the macroeconomic effect of mortality that could be prevented with access to high-quality care in LMICs. The analysis was done by use of two distinct approaches to quantify economic losses from preventable mortality. The first approach projected gross domestic product (GDP) losses over 15 years due to the consequences of mortality on labour force and physical capital accumulation. In 91 LMICs, amenable deaths due to insufficient good quality care would result in a projected cumulative loss of US$11·2 trillion (UI 8·6–15·2 trillion) between 2015 and 2030. This economic output loss was greatest in low-income countries, costing 2·6% of their GDP compared with 0·9% in upper-middle-income countries.^95^ The second approach estimated the current value of total economic welfare losses on the basis of the concept of a statistical life, which attempts to capture the value placed on good health in and of itself. In 2015 alone, poor access to quality care resulted in an estimated $6·0 trillion of losses in 130 LMICs.^95^ Upper-middle-income regions lost the least, whereas losses in sub-Saharan Africa accounted for more than 15% of GDP. This analysis shows that poor-quality care can result in a great macroeconomic burden that is inequitably distributed across countries.

Beyond the economic losses from premature mortality, poor-quality care can also lead to important waste and inefficiency. Waste in health care has been defined as any “health-care spending that can be eliminated without reducing the quality of care”.^123^ Health-care waste includes the overuse of unnecessary care or ineffective approaches, medical errors, unsafe care, incoordination of care, misuse (including inappropriate hospital admissions and bypassing), fraud, and abuse. There have been few measurements of health-care waste attributable to poor-quality care in LMICs. However, evidence from high-income settings suggests that averting these costs could help LMICs make better use of scarce resources. For example, the annual costs of extra hospital stays and readmissions for treatments of surgical site infections were estimated to range between $3·5 billion and $10 billion in the USA and between €1·47 billion to €19·1 billion in Europe.^73^ Similarly, the global economic effects of antimicrobial resistance remain largely unknown, but in the USA alone, its yearly cost to the health system is estimated to range between $21 billion and $34 billion.^124^ Lastly, the global cost of unnecessary caesarean sections done each year is estimated to be $2·32 billion, which far surpasses the cost of needed caesarean sections.^125^ Because care delivered in hospitals has a greater risk of complications and is more costly, inappropriate hospital admissions also represent a substantial burden to the health system. High-quality primary care can prevent the need for hospital admissions for several health conditions called ambulatory care-sensitive.^11^ In the USA, $31 billion are spent annually on hospital admissions for these conditions.^123^ Better perceived quality and greater trust in health systems can also improve care-seeking patterns and reduce the bypassing of primary care facilities for overcrowded hospitals in LMICs.

Finally, people living in countries with poorly functioning health systems, without appropriate financing mechanisms and insurance, risk suffering from catastrophic or impoverishing expenditures when seeking care. Out-of-pocket payments (ie, health spending made by patients themselves at the point of care) as a share of household consumption have been increasing worldwide.^126^ In 2010, 808 million people (11·7% of the world’s population) incurred catastrophic health expenditures—ie, exceeding 10% of household consumption.^17^ Catastrophic spending increased by 2 percentage points since 2000 and was associated with economic growth and per capita health spending. Nearly 100 million people are pushed into extreme poverty each year because of out-of-pocket expenses.^17^ For poorer households, out-of-pocket payments often mean choosing between paying for health and paying for other necessities, such as food or rent, straining their day-to-day survival capacity and affecting their physical, social, and economic wellbeing.^127^ High-quality health systems with appropriate financing mechanisms can enable facilities and providers to give affordable care to the population. To help reduce impoverishing and catastrophic expenditures, prepaid health expenditures should replace out-of-pocket payments. A study^128^ published in 2018, found that the proportion of the population covered by health insurance schemes or by national or subnational health services was not associated with financial protection. Conversely, increased shares of prepayment in total health expenditure, typically achieved through taxes and mandatory contributions, were important for protecting people against catastrophic spending.^128^

The economic consequences we have described could be attenuated or averted in high-quality health systems. However, improving health system quality will require additional investments in many countries. Analyses have suggested that these will be substantial but affordable in most settings, excepting the poorest countries. In 2017, WHO published^129^ an estimation of the cost of interventions and health-system strengthening strategies required for reaching all SDG-related health goals in 67 LMICs. WHO estimated additional annual costs of $263 billion, which would save 97 million lives from 2016 to 2030. The estimated total costs per person ranged from $112 in low-income countries to $536 in upper-middle-income countries. The Disease Control Priorities Project^14,130^ estimated the costs for reaching 80% effective coverage for 218 interventions, to meet UHC targets, in 83 LMICs and found that an additional $260 billion per year would be required. This represents $76 per person in low-income countries and $110 in lower-middle-income countries; this investment would result in 6·2 million deaths averted by 2030. Further research is needed to measure the costs of specific quality improvement strategies, including those advanced by this Commission.

**Figure 9:**
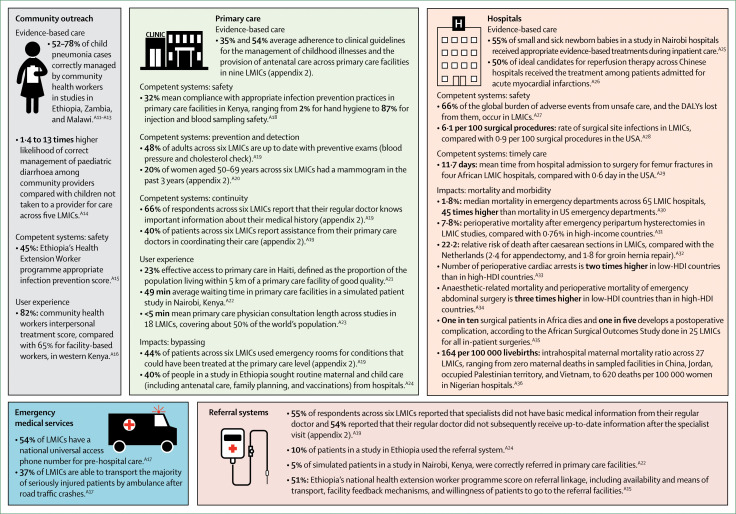
Quality of care across health system platforms in low-income and middle-income countries (LMICs) DALYs=disability-adjusted life-years. HDI=Human Development Index. References can be found in appendix 1.

A health systems view must also be used to understand quality. This section addressed health care that is delivered at different levels of the health system, including through community outreach, primary care, and hospital care, and the linkages between them—referral systems and emergency medical services. Figure 9 summarises evidence on quality across these key health system platforms.

### Equity of high-quality care

We have thus far reviewed the available evidence on quality of care at a national or multinational level. However, these estimates mask important variations within countries. Equitable distribution of high-quality health care is essential to make the gains in health set out by the SDGs and ultimately contribute towards the realisation of the right to health. We now explore why some groups are more vulnerable to poor-quality care than others and who receives worse quality care.

#### Defining equity in the quality of health care

Braveman and Gruskin^131^ defined health equity as “the absence of systematic disparities in health (or in the major social determinants of health) between groups with different levels of underlying social advantage/disadvantage—that is, wealth, power, or prestige”. This definition emphasises equitable health outcomes. The health-care system is one major determinant of health, and equitable access to the system is, therefore, important. But equitable access will not result in more equitable health outcomes unless all people—not just the privileged—are able to access high-quality services. Equity in the quality of health care can be defined as the absence of disparities in the quality of health services between individuals and groups with different levels of underlying social disadvantage.

#### Groups vulnerable to poor quality of care

In 1971, Julian Tudor Hart^132^ stated that “the availability of good medical care tends to vary inversely with the need for it in the population served.” There is evidence of this inverse care law in many health systems—LMICs and high-income countries alike. For instance, tuberculosis has a strong socioeconomic gradient between countries, within countries, and within communities.^133^ Drug resistance arises in areas with poor tuberculosis control programmes and among subpopulations that face barriers to quality treatment. Similarly, a systematic review^134^ focused on diabetes showed that low individual socioeconomic status and deprivation in the residential area are associated with worse process indicators and intermediate outcomes, resulting in higher risks of microvascular and macrovascular complications.

The 2030 agenda for sustainable development is built on principles of universality and aims to ensure that no one is systematically left behind.^135,136^ This commitment is echoed in the World Health Assembly resolution number 69·11,^137^ which calls for “health system strengthening for UHC, with a special emphasis on the poor, vulnerable, and marginalised segments of the population”. Therefore, an effective implementation demands the defining and targeting of those most vulnerable.^136^ WHO’s definition of vulnerability encompasses the effects of “marginalisation, exclusion, and discrimination that contribute to poor health outcomes”.^138^ Vulnerability can vary substantially, change over time, and be multidimensional.^139^ Factors such as gender, ethnicity, displacement, disability, and health status can increase vulnerability of both individuals and communities. These factors are often fluid and have intersecting points, presenting serious obstacles to individuals in accessing high-quality health services.^139^ However, many countries fail to recognise the existence and impact of intersecting discrimination. As a result, the experiences and needs of these populations are not integrated into national health strategies, further entrenching the discrimination and disadvantage that they face.

**Figure 10:**
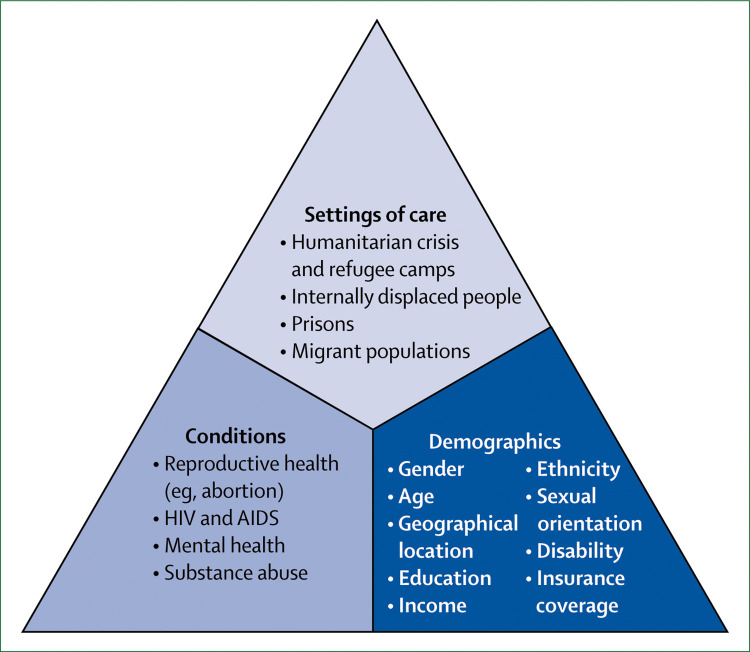
Dimensions of vulnerability to poor-quality care

In this Commission, we highlight three dimensions that might make people especially vulnerable to poor-quality care: settings of care, conditions, and demographic factors (figure 10). Within settings of care, vulnerability is greater for individuals on the margins of mainstream services or displaced from home, such as those who are in a humanitarian crisis or in refugee camps, internally displaced, living in informal settlements, prisoners, and migrant populations. People with stigmatised conditions can face worse treatment in the health system than others; these conditions can include HIV and AIDS, mental health and substance abuse disorders, and some reproductive health services such as abortion. Finally, previously recognised social and demographic factors that indicate asymmetric power, such as gender, age, sexual orientation, ethnic group, disability, and insurance coverage, can predispose people to experiencing poor-quality care.

Reasons for poor-quality care in these three dimensions include the collapse of health services, insufficient financial and human resources, low patient empowerment, barriers to continuity of care, insufficient legislative controls, and breakdown in trust between patient and system. These dimensions of vulnerability, along with an understanding of why these groups could receive poor-quality care and suffer worse health outcomes than others, can inform policies and programmes that target specific vulnerability factors.

*Panel 4:* Why quality of maternal mental health care might suffer for vulnerable groups: perinatal depression care in primary care setting in Nigeria*

Women with perinatal depression can experience stigma associated with mental illness in some low-income and middle-income countries. People with mental disorders are often victims of discrimination and denial of basic rights.^A37^ They can also internalise shame, anticipate rejection and discrimination, and accept diminished expectations from others. These two forms of stigma, enacted and felt, have the effect of exposing individuals with mental disorders to poor and inequitable quality of care. Therefore, in the context of perinatal depression, stigma would increase the likelihood that those suffering are denied access to the basic and often rudimentary services available.

A formative study done as part of the project Scaling up Care for Perinatal Depression for Improving Maternal and Infant Health in Nigeria, assessed the factors that might promote or hinder the delivery of quality services to women with perinatal depression (appendix 1). All 23 facilities sampled had the lowest level of institutional support for continuous care for depression. Of the 218 patients who screened positive for perinatal depression by use of a validated tool, only three were identified by primary health-care workers. The treatment offered to these three patients was non-existent or grossly inadequate. None were provided with structured psychosocial interventions or offered specific follow-up to address their depression. However, 96% of the women in all sampled facilities reported that the quality of care provided in the clinics was good and of sufficient quality, and 98% reported that they were satisfied with the care they had received.

The low capacity of all the sampled facilities to provide quality care for depression, and the extremely low detection rates of depression by primary health-care workers recorded in the study showed important gaps in both the organisational structures and the manpower capacity of the front-line facilities to respond to common perinatal mental health conditions in a fully functional integrated chronic care model. Despite the objectively rated poor quality of service being provided, the women using these facilities still rated them high regarding quality of care and personal satisfaction with the level of service provided. This paradox is an important indicator of the existing inequity in the system: people who have never experienced high-quality services set their expectations low and do not know how to demand higher-quality health care.

Source: Olatunde Ayinde and Oye Gureje. *Panel references can be found in appendix 1.

Panel 4 and panel 5 illustrate how conditions (eg, mental health) and settings of care (eg, humanitarian crisis or refugee camps) can exacerbate poor-quality care and what might be done to address these inequalities.

#### Who receives worse quality care?

The monitoring and tracking of equity in health intervention coverage has been the focus of major international efforts.^140^ Many studies^61,140,141^ have shown that some population groups are systematically less likely to have access to or use health services for several conditions. However, there has been less work done on equity in the quality of care. As described earlier in this section, quality of care varies between and within countries. Quality of care can also vary between certain population groups and across conditions in the same area. For example, a study^142^ in Kenya showed that the quality of labour and delivery care was generally low, but care available to the poor was substantially worse than that for wealthier people. Similarly, it was found that in Madhya Pradesh, India, poor people living in poor communities received especially poor-quality care.^143^ Additionally, poor people throughout the world live and die with little to no palliative care or pain relief.^36^

We disaggregated several indicators of quality in maternal and child health presented earlier in this section by wealth, urban and rural residence, maternal age, gender, and education (appendix 1); we also assessed variation in quality between the public and private sector. We found evidence that quality care is inequitably distributed across these stratifiers.

**Figure 11:**
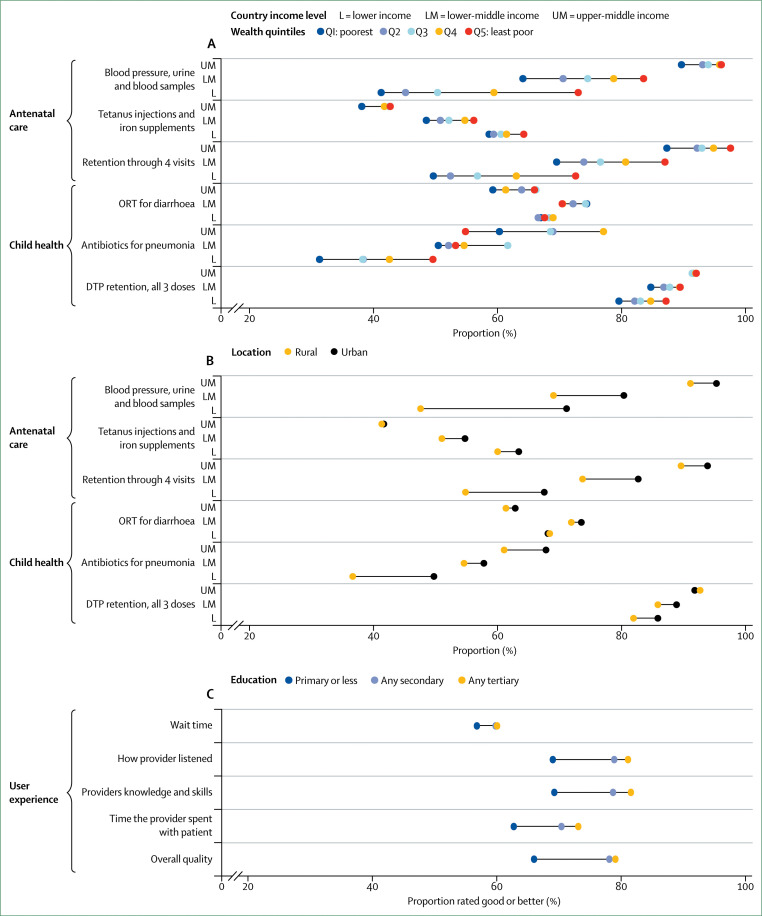
Equity in maternal and child health-care quality and in user experience in low-income and middle-income countries (LMICs) (A) Data are from Demographic and Health Surveys and Multiple Indicator Cluster Surveys done in 90 LMICs (2007–16); wealth quintiles are pooled across countries and sampling weights are adjusted to weigh countries equally. (B) Data are from Demographic and Health Surveys and Multiple Indicator Cluster Surveys done in 91 LMICs (2007–16) and are weighted using individual-level survey weights. (C) Data from Commission-led internet survey in 12 LMICs (2017); proportion of respondents who classified their experience for each indicator as “good”, “very good”, or “excellent” (*vs* “fair” or “poor”) for their last outpatient visit within the prior 12 months; education levels are pooled across country. Indicators are defined in appendix 1. ORT=oral rehydration therapy. DTP=diphtheria tetanus pertussis vaccine.

Regarding evidence-based care, figure 11A shows the proportion of women and caregivers reporting different elements of antenatal and child health care by wealth quintiles. We found evidence of a wealth gradient across most of these indicators. Among women attending antenatal care with a skilled provider, wealthier women were more likely to report receiving antenatal care assessments and appropriate preventive treatments and more likely to be retained in care until the fourth antenatal care visit. For example, among women attending antenatal care, we found that the wealthiest were four times more likely to report blood pressure measurements and urine and blood tests than the poorest women in their country (relative index of inequality 4·0, 95% CI 3·9–4·1).^45^ When seeking care at facilities for pneumonia, children in the wealthiest quintiles in low-income countries were more likely to receive antibiotics than those in the lowest; among all children who received the first diphtheria, tetanus, and pertussis vaccine dose, those from wealthier families were more likely to complete the vaccination series (receiving the third dose by age 1 year) than children from poorer families. These inequities tended to be larger in low-income countries than in lower-middle-income and upper-middle-income countries.

*Panel 5:* Quality of humanitarian health services for populations affected by armed conflict and natural disasters*

During 2016, there were 49 active armed conflicts with about 170 million people affected, including 60 million refugees and internally displaced people throughout the world.^A38–A40^ Additionally, an estimated 200 million people are affected by natural disasters annually.^A41^ These crises cause excess morbidity and mortality through multiple pathways.^A42^ One of these is the disruption of what are often already weak public health systems. In most crises, the health system undergoes substantial degradation and fragmentation, with the void left by reduced government activities often filled by faith-based, private, and informal providers.^A43^

There are logistical, safety, and practical difficulties in undertaking research during times of conflict that have led to insufficient data on the quality of health services being provided in these situations.^A44^ However, methods that have been used to assess the quality of care showed low levels of competent care and user experience, issues with staff motivation, and less complicated conditions receiving better quality care than patients who were seriously ill.^A45^ During the past two decades, humanitarian actors have undertaken various, largely normative, initiatives to promote health-care quality. However, accountability and enforcement remains low, and few humanitarian agencies have implemented health governance systems. Here, we discuss several challenges that need to be tackled to advance the quality agenda in the humanitarian health sector.

First, the pursuit of quality remains weak and needs to be incentivised. For example, donors of humanitarian activities should place greater emphasis and funding on strengthening the use and reporting of quality standards and performance metrics. Failure to collect and report these data should have consequences for agencies, such as removal of permission to operate and loss of funding. Second, quality is impeded by insufficient capacity within the humanitarian health workforce. Efforts to professionalise the humanitarian health workforce need to be scaled up through training and updated technical standards and competency frameworks. Third, existing coordination mechanisms need to evolve into technical leadership arrangements, whereby, in exchange for the benefits of taking part in coordination (eg, access to specific funding pools), actors agree to operate according to a standard package of care and specific service quality standards. Fourth, governments need to explicitly consider crisis areas when implanting health interview and population surveys. The actors in these areas should collect data in a way that matches the quality indicators defined by the public health information systems, including assessment of confidence in the system. Lastly, health governance in the humanitarian systems remains weak. Robust governance arrangements, ideally interagency, need to be established to develop concrete accountability and liability in the humanitarian health sector.

Source: Bayard Roberts and Francesco Checchi. *Panel references can be found in appendix 1.

We also found important urban–rural differences in several of these quality indicators, whereby women and caregivers in urban areas were significantly more likely to report better maternal and child health-care quality than those in rural settings (figure 11B). These urban–rural differences were also largest in low-income countries.

In terms of user experience, this Commission’s 12-country internet survey also showed that people with some primary education consistently rated their user experience as worse than did those with secondary education or higher (figure 11C). The largest gap was found in the rating of the overall quality of the last outpatient visit, for which people with primary education or less reported significantly lower quality than did those with more education. A total of 34% of respondents reported that staff had treated them poorly because of their identity and, of those, 10% attributed this to their poverty (appendix 1). These inequalities could be underestimated because studies have shown that less educated people tend to be more accepting of the care they receive.^144,145^

Additionally, adolescent women seeking maternal and child health care can also face particular stigma and poorer quality care (appendix 2). Among women attending antenatal care and delivering in health-care facilities, young adolescents were less likely to report receiving different elements of care than women aged 20–35 years. Younger mothers were less likely than others to receive post-partum checkups before discharge after giving birth in a health-care facility. The youngest adolescents (15-year-olds) appeared to be substantially less likely to receive all four recommended antenatal care visits, and their children were less likely to complete the diphtheria, tetanus, and pertussis vaccination series.

An analysis of data from the STEPS survey on receipt of lifestyle advice from health-care providers among adults diagnosed with diabetes, hypertension, or hypercholesterolaemia found that women were less likely to receive advice about tobacco use and physical activity than men, and overall, those with no formal schooling were more likely to receive advice about tobacco use and dietary change than those with primary or secondary schooling. Individuals with secondary schooling were more likely to receive advice about physical activity, maintaining a healthy bodyweight, or losing weight than those with primary or no schooling. Additionally, evidence from the Prospective Urban Rural Epidemiology study^146^ found that the use of medication for secondary prevention of coronary heart disease was extremely low, with people in the poorest countries having the lowest rates of use. Within countries, women and rural dwellers had lower use than men and urban dwellers; less educated patients were less likely to use antiplatelet drugs and statins than more educated patients.

Quality can also differ between public and private facilities, but these differences vary across contexts. Such differences also depend on the types of provider included in the definition of private sector. In terms of evidence-based care and competent systems in the Democratic Republic of the Congo, Kenya, Rwanda, and Uganda, adherence to WHO guidelines for sick child care was higher in private facilities than in public ones. Additionally, adherence to checklists was higher among private providers than among public ones in a standardised patient study^52^ in India. However, an analysis^147^ of household surveys in 46 countries found that public and private sectors did similarly in terms of antenatal care quality. By contrast, a systematic review^148^ in LMICs found that private sector providers (including unlicensed and uncertified providers) were less likely to follow medical standards of practice, had poorer patient outcomes, and reported lower efficiency than public sector providers, resulting partly from perverse incentives for unnecessary testing and treatment. For user experience, public providers did worse in terms of timeliness and hospitality to patients than private providers.^148^ Nonetheless, quality can vary considerably within the same sector in a country. Additionally, country differences were found to be more influential than all other subnational factors combined in explaining variation in the quality of primary care services and labour and delivery care.^38^ This finding might point to the importance of structural factors in producing quality.

*Panel 6:* Section 3 key findings

1. Previous right-to-health discussions did not sufficiently elaborate on the quality of health services promised to people
2. Spending scarce resources on expanding access to services without ensuring quality is wasteful and inefficient; as countries embark on universal health coverage, services should be accompanied by a national guarantee of quality
3. Quality improvement efforts should start in areas with the greatest quality deficits, with a focus on care received by disadvantaged populations
4. There are concrete mechanisms available to improve health system accountability; this lies at the core of realising the right to the highest attainable standard of health for all people

### Section 2 conclusion

The epidemic of poor-quality care described in this section casts doubt on the ability of legacy health systems to achieve the SDG health targets. Poor-quality care in LMICs is reflected by inadequate adherence to evidence-based care, negative patient experiences, unequal treatment and access to health services, and by deficiencies in safety, prevention, continuity, and timeliness, leading to poor health, adverse economic outcomes, and loss of trust and confidence in health systems. Additionally, poor and vulnerable groups appear to experience worse quality care. Despite the breadth of the evidence presented in this section, there were still many gaps in the availability of data on quality of care (appendix 2).

Poor-quality care has been attributed to the poor knowledge and competence of providers and to fatigued or unmotivated health workers. However, the scale and range of the problem across countries, settings, and health conditions suggests that it is a manifestation of a broader systems failure. LMIC health facilities are underequipped, overcrowded, and frequently understaffed. Pre-service education and specialty trainings are inadequate. Processes are inefficient or inexistent, including financial incentives and remuneration of providers, referral networks, and triage in emergency departments. These fragmented health-care systems are unable to support health workers in providing high-quality care.

## Section 3: The ethical basis of high-quality health systems

The core principle of this Commission is that health systems are for people. This section asks: are they for all people? We review the right to high-quality care and provide insights into steps that national governments and communities can take to address the issue of equity and build a strong high-quality health system that targets the poor and vulnerable groups. The key findings of this section are shown in panel 6.

### Implementing the right to high-quality care through a national quality guarantee

#### What is the right to quality care in settings with few resources?

The health and human rights agenda has been essential to motivating investments and actions to improve health in LMICs, as well as globally. This agenda historically emphasised inputs and access to care, but did not specify the quality of services provided. In 2000,^13^ the UN Committee on Economic, Social, and Cultural Rights adopted general comment 14, which states that the right to the highest attainable standard of health includes availability, accessibility, acceptability, and quality. In a review for this Commission^149^ of global health policy milestones since 2000, we found that the global discourse has been focused on access to care and foundations of quality, but not enough appears on processes of care or quality-specific impacts, such as trust or satisfaction. However, with the implementation of the 2007 WHO framework for action on strengthening health systems to improve health outcomes and the 2016 WHO framework on integrated, people-centred health services, the trend is moving in the direction of patient-centred care and measures of quality focused on processes of care. Health systems should communicate the right to health through a national health plan, initiatives to ensure that the public knows its entitlements and how to realise them, and data on health system quality.^30^

### Are there ethical trade-offs between improving quality and expanding access?

One reason that quality has lagged behind access in global health discussions is the perceived trade-off between expanding coverage and improving quality. A trade-off is a compromise between two or more desirable, but competing considerations and, thus, involves a sacrifice made in one dimension to obtain benefits or ensure respect for rights in other dimensions.^150^ There was (and still is in many low-income countries) an understandable sense of urgency to expand essential services to the population at any cost—without an explicit focus on quality. This finding can be interpreted as the result of a trade-off made by decision makers: equitable access for all is better than access to high-quality services for some.

Quality is essential to the equity agenda. We recognise that on the high end of care, such as expensive advanced technologies and medicines, provision of cheaper and somewhat less effective treatments can be an appropriate option in low-resource settings. One example is the use of the visual inspection with acetic acid method for cervical cancer screening instead of the more expensive and time consuming Papanicolaou smear and human papillomavirus co-testing.^151^ However, we believe that a concern for equity implies access to a minimally assured level of quality for all. There are two reasons for this: ethical achievement of health outcomes and efficient use of resources. First, increased access will not translate to better health outcomes for disadvantaged people unless all people have access to high-quality services. Second, spending scarce resources to expand access without quality is wasteful and inefficient. Countries can build on their achievements in expanding coverage by improving the quality of services offered to meet the minimum quality level. They can then consider further expansion of quality services.

As countries pursue UHC, approaches such as progressive universalism—a determination to include people who are poor from the beginning—have proven to be effective ways to target poor and vulnerable groups of society.^152,153^ Brazil’s Family Health programme^154^ and Mexico’s Seguro Popular initiative^155^ are two examples of programmes designed to increase coverage first among disadvantaged groups. This Commission endorses this approach.

#### Defining a national quality guarantee

Many countries recognise the need to be accountable for the health care of the population. One clear manifestation of this is patients’ rights charters that outline a country’s approach to patient care and provide an ethical basis for care. Although these charters contain many of the same basic principles, such as legal and human rights guarantees, they vary substantially in length, scope, and detail. Patients’ rights charters are well intentioned, but not operational. South Africa is attempting to make its promises actionable through its National Health Insurance Policy, which underpins the establishment of a unified health system based on the principles of social solidarity, progressive universalism, equity, and health as a public good and a social investment (appendix 2).

This Commission recommends that countries adopt a national quality guarantee—ie, quality sufficient to consistently produce a health benefit. This would be concrete and operational for covered services. What are the elements of such a guarantee? First, clearly poor-quality services, providing more harm or risks than benefits, fall below the thresholds of a guarantee. Second, the quality of services must be sufficient to generate health benefits. For example, a rural clinic should specify to the patient the level of services that it is competent in providing. Third, services must be provided in a respectful people-centred manner. An integral aspect of people-centred health systems is the relationship between provider and patient. Patient–provider relationships are shaped by societal norms and are susceptible to power imbalances. Pre-service and in-service training on respectful care is one way to improve the ethical competence of providers in low-income settings.^156^ However, to end the poor treatment of patients and greatly improve health care, people-centred and patient-driven approaches that shift the power from the health-care system and providers to the patients are needed.^157^

The quality guarantee should accompany any efforts to expand service coverage; in many countries, the movement to UHC is an excellent starting point. National standards for conditions covered by a UHC benefit package might include descriptions of adequate assessment and diagnosis, treatment and care, assurance of continuum of care, and referral. This is a corrective to the current UHC discussion that revolves around the pooling of funds to expand the coverage of populations and services while decreasing the cost. Without building in quality, the increased coverage will not result in health gains for people. Although many countries can do more to provide quality health services with existing funds, others will require additional funds. Data from WHO^4^ show that global government spending on health as a percentage of all government expenditures rose by an average of 10% between 2000 and 2015; however, it was flat in lower-middle-income countries, and fell substantially in low-income countries—the very countries struggling with poor-quality care.

Beyond these general considerations, countries need to undertake analyses and open discussions to specify their national standards. National guarantees should start with the reality of social norms and health system functions and be context-specific.^158^ Guarantees will depend on budget, setting, disease type, intervention, and delivery platform. Current national standards are often defined and implemented through standard operating procedures or clinical practice guidelines. Standards included in the national quality guarantee should be developed by health policymakers and professionals, in collaboration with users and national regulatory agencies, to ensure that upholding the guarantee does not fall solely on providers. The guarantee is not intended to be punitive against individual providers; any redress mechanisms should be targeted to the appropriate level of the health system.

### Improving accountability for quality

Over the past three decades, the concept of accountability in provision of health care has gained increased attention. However, accountability for quality in health care has been less explored. In this subsection, we refer to Brinkerhoff’s definition^159^ of accountability, which encompasses both answerability and enforceability. The three general categories of accountability are financial, performance, and political or democratic. In this section, we use elements of financial and political or democratic accountability to discuss legal and social mechanisms. Performance accountability is discussed in the subsequent sections. For accountability to function, there must be actors responsible for activities, standards to define what actors should deliver, agents to hold actors to account, and tools or methods to do so.

A review done for this Commission on the accountability ecosystem and its relation to the delivery of quality care (methods in appendix 1) supported the notion that accountability mechanisms can serve as a catalyst to initiate and sustain improvements in quality and advance the progressive realisation of the human right to health and quality health care. The review found that multiple accountability tools have been used, and documented in the peer-reviewed literature, to improve access to essential and effective health care (appendix 1). A key finding of the review was that single interventions do not have the power to induce large-scale change. Additionally, governance and coordination must be strengthened, resources must be planned and budgeted, and a performance monitoring system must make the information collected available. Therefore, to improve quality, countries need to devise accountability strategies that encompass elements of legal and social accountability.

#### Legal accountability

National governments are the primary agents for accountability. Human rights conventions can provide the basis for legislation that recognises the right to health and health care, and can be an essential and minimum foundation for approaches to improve access and quality of care. Meaningful legislation should not only recognise the right to health and health care, but also cater for the right to meaningful public participation, freedom of civil society, and freedom of information. Where such legislation exists, it can be used for accelerating action. Quasijudicial mechanisms exist in many LMICs, such as the ombudsman in South Africa tasked with addressing the system failures that led to the deaths of 94 mental health-care users.^160^ Also in South Africa, the Treatment Action Campaign defeated the Government in the constitutional court to increase access to HIV treatment to mothers and newborn babies. A high court in Kenya awarded a woman 2·5 million Kenyan shillings for mistreatment and abuse during childbirth, which was caught on film.^161^ Additionally, in Malawi and Mozambique, human rights concerns and entitlements were used by civil society organisations to expand national policy for maternal, newborn, and child health.^162^

#### Social accountability

Social accountability refers to approaches that involve communities, citizens, and service users directly; these approaches include attempts to increase community involvement, awareness, and demand generation for high-quality care.^163^ A 2004 World Development Report^164^ suggested that social accountability tools could be used to increase transparency and accountability, shortening the long route of democratic accountability between citizens and politicians. Multiple tools are available to foster social accountability. They include citizen report cards, community monitoring, social audits, participatory budgeting, citizen charters, and health committees. Mechanisms for creating and acting on such tools exist in LMICs today. Institutions tasked with reporting on quality-related indicators include the Health Data Advisory and Coordinating Committee in South Africa and the General Directorate for Quality Healthcare and Education in Mexico.^165^ There are licensing and assessment activities with internal and occasionally public reporting, such as the Ideal Clinic in South Africa, Big Results Now project in Tanzania, and the Kenya Patient Safety Impact Evaluation.^166–168^ Finally, direct public reporting of local progress can be effective, such as Imihigo,^169^ the televised reporting of progress on commitments by local leaders in Rwanda, including maternal health outcomes. These social accountability mechanisms should be seen as complementary rather than substitutes to the legal approaches previously discussed.

*Panel 7:* Actions to support legal and social accountability

A literature review done for this Commission aimed to present findings on the accountability–quality relationship and explore how accountability mechanisms contribute to improvements in quality of care. The review focused on legal and social accountability mechanisms pertaining to reproductive, maternal, and child health. The key findings were synthesised and the following actions were identified as important for effective and transparent accountability:

- Adopt and enact legislation that recognises the right to health and quality health care
- Invest in rights awareness and education at all levels, including among policy makers, parliamentarians, programme managers, service providers, and the public
- Share information on health system performance with the public and promote transparency of quality measurements
- Institutionalise mechanisms for remedy and redress, such as ombudsperson or tribunals
- Develop multipronged strategies for accountability for quality of care that combine legal, performance, and social accountability tools

Methods are described in appendix 1. Source: David Clarke, Rajat Khosla, Blerta Maliqi, Marcus Stahlhofer, and Bernadette Daelmans.

Panel 7 synthesises the key findings from the review on legal and social accountability and proposes actions to support effective and transparent accountability at the national level.

### Section 3 conclusion

Health systems should give priority to poor and vulnerable groups of society to reduce inequities and expand the right to quality health care through progressive universalism. A movement towards UHC offers countries the opportunity to start on this path by expanding coverage tied to a national quality guarantee. Legal and social accountability mechanisms can assist in upholding these quality standards. Enacting accountability is predicated on insight into current health system quality. In the next section, we assess the purpose, status, and promise of health system quality measurement.

## Section 4: Measuring health system quality

The key findings of this section are shown in panel 8.

### Why measure health system quality?

Valid and reliable information is a necessary input to a high-quality health system.^170,171^ Multiple national, international, and global efforts are underway to identify measures to improve care delivery and amplify patient voices. These efforts include the National Quality Forum in the USA, the Health Data Collaborative, and initiatives undertaken by OECD, the Inter-American Development Bank, and the China Joint Study Partnership.^70,76,172–175^ These efforts show that the measurement of health-care quality is a concern of populations and governments around the world; high-income settings, in particular, have invested in institutions to strengthen health system performance through measurement. Although some efforts, such as the Health Metrics Network, have included LMICs, country ownership of this agenda has been inconsistent, and progress on health system measurement remains incomplete.

*Panel 8:* Section 4 key findings

1. Accountability and action are the guiding purposes of quality measurement; measurement not used for these purposes can burden the health system.
2. Current quality measurement is fragmented by disease, focused on inputs rather than outcomes, and poorly aligned to population health needs. Decision makers do not have timely information that provides a picture of the health system as a whole.
3. National and global actors should seize three opportunities to improve measurement of health system quality: (1) measure effective coverage—use quality-corrected coverage metrics to track progress towards UHC; (2) adopt fewer, but better measures by shedding inefficient indicators and prioritising measures of system competence, user experience, and outcomes, including clinical and patient-reported health, confidence in the system, and economic benefit; (3) invest in country-led quality measurement, including strengthening national capacity for data use and policy translation, releasing an annual health system quality dashboard, and disaggregating results for vulnerable populations.

Indeed, the findings described in Section 2 on healthcare quality in LMICs reveal crucial measurement gaps. Existing data on quality of care have largely been generated within vertical programmes, resulting in measures that have not been combined in ways that could illustrate quality of the health system as a whole, whether at local or national levels.^176^ Systematic data on the performance of health system platforms (such as primary care) or on user experience, population confidence, and patient-reported health outcomes are scarce. Moreover, research on health system quality—including the policy and implementation research urgently needed to bring effective interventions to scale—has not kept pace with the magnitude of the challenge, reflecting inadequacies in measurement approaches and data use. A bibliometric search for quality-related research between 2000, and 2016, revealed that, although this type of research is increasing in LMICs, it remains overwhelmingly located in high-income countries (appendix 2).

The demands made of health systems are growing: the burden of disease is shifting towards non-communicable diseases and injuries,^6^ health emergencies are rising,^177^ countries are actively moving towards UHC,^17^ and people are demanding better services and outcomes.^119^ The health priorities of the SDG^178^ era—with ambitious targets of improved survival and quality of life for all—demand new approaches that promote accountability and action to drive broad health system improvements. To meet these challenges, measurement approaches need to be responsive to new health system demands, relevant to people, and efficient. At the heart of this reframing is the question: why measure and for whom? This Commission proposes two main purposes for the measurement of health system quality: accountability and action.

Accountability requires the provision of information when questioned, whether for routine monitoring or detailed justification, paired with a mechanism for oversight.^159^ This section focuses on measurement for performance accountability—how the health system delivers on its intentions—and social accountability—whether it is responsive to society.^159^ The measurement of performance accountability should show results against benchmarks, support crossnational or subnational comparisons, disaggregate evidence for vulnerable subpopulations, and do this in or near real time. For both performance and social accountability, data will typically need to be representative of the target population, comparable, and systematic. Measurement should further include elements that are of high value to people; for example, they should include not only health outcomes such as survival, but also function, pain, and processes such as respectful treatment (panel 9).

*Panel 9:* What different measures tell us about health system quality*

Measures of health system quality have usually been organised into inputs (eg, workforce, tools, facilities), processes of care (eg, adherence to guidelines, communication), and outcomes (eg, morbidity, mortality).^A46^

In low-income and middle-income countries, many quality measurement and improvement efforts have emphasised inputs to health services. Inputs are foundational to health-care provision and are easily measured, but they provide narrow insight into quality of care. Studies have found weak associations between input measures and care competence,^A47^ particularly when facility size is considered.^A25^ The relation between input availability and the quality of care received can differ over the course of care delivery,^A48^ underscoring the need for motivated and competent providers and supportive systems for good care delivery. Similarly, multiple studies^A49,A50^ attest to the know–do gap: the deficit between provider knowledge and the clinical care provided. These issues do not mean that input measures are unimportant; indeed, timely and specific information on inputs, such as stock levels and equipment functionality, is crucial for health service planning and operation and should be collected by health systems. However, these measures should not be used as indicators that health systems are providing high-quality care.

Process measures can play an important role in illuminating the quality of care provided. These measures are immediate and relevant at the point of care, and they provide direct insight on care provision without risk adjustment, which makes them particularly valuable in assessing gaps or disparities in care for vulnerable subpopulations.^A51^ A judicious selection of process measures is essential, emphasising measures validated against the outcomes that matter to patients,^A52,A53^ whereas overmeasurement can divert provider time and weaken the quality and usefulness of data. The proliferation of process measures in high-income countries, for example, has increased the burden of measurement and resulted in unintended consequences, including fixation on the measure rather than the intent, reallocation of efforts towards meeting measurement targets and away from other essential tasks, and gaming (manipulation of the quality assessment system).^A54,A55^

Health outcome measures attest to the central goal of a health system—maintaining or improving health and wellbeing.^A56,A57^ However, these measures can be challenging to attribute directly to health system performance because of the involvement of multiple factors. Baseline risk information is required for valid comparisons of health outcomes over time or between facilities.^A58^ Despite this complexity, there is global recognition of the crucial need for health-system-sensitive and patient-focused outcome measurements, even in very low-income settings.^A56^ Health-system-sensitive outcomes include perioperative mortality, inpatient suicide, 5-year cancer survival, obstetric fistula, caesarean section, unsuppressed HIV viral load, uncontrolled blood pressure, lower extremity amputation in patients with diabetes, and hospitalisation due to ambulatory care-sensitive conditions. High-income settings are increasingly turning to patient-reported outcomes (PRO) as a means of realigning health care with patient values.^A59^ PRO measures have been used to improve monitoring, decision making, and patient–provider communication,^A60^ with evidence suggesting that the use of these measures improved patient perceptions of care^A61^ and led to better health outcomes for some conditions,^A62^ although their usefulness in aggregate has yet to be fully demonstrated.^A63^ Routine measurement and the use of health-system-sensitive outcome data and PRO are integral to achieving patient-centred health systems.

*Panel references can be found in appendix 1.

Measurement for action is at the heart of learning health systems. These measures should provide decision makers with answers to specific questions about the functioning of the health system and the quality of care delivered, help identify the targets and interventions for improvement, and monitor the results of the changes implemented. Quantitative data should be complemented by so-called soft intelligence, the insight on the context and processes of care delivery, to help inform action.^179^ The focus of measurement for action is likely to differ in a complex adaptive system: acting directly at the level of the process indicator (eg, attempting to address poor adherence to guidelines with printed reminders) might not yield expected effects if the indicator merely signals a deeper quality deficit at the health-system foundation level.^158,179^ Measurement for action is discussed further in Section 5.

Fulfilling either purpose of measurement—ie, for accountability or action—requires valid and reliable measures, transparency in information exchange, and an entity with the power to demand a response. Panel 10 outlines conditions required for measurement to induce change.

To meet the SDG targets and improve health system quality by 2030, countries will need to embark on a measurement agenda that will take time and investment to fulfil. This agenda starts by knowing what is currently being measured.

### What is—and is not—measured in LMIC health systems today

Multiple strategies have been used to capture the range of information needed to assess health system quality, including measuring population health needs, health outcomes, and health system performance. Table 2 describes the platforms in use and their best application; given the multiplicity of tools, central organisation and triangulation are needed to gain insight and act on these data. We, and others, have found that health system data collection is often costly, uncoordinated, and disconnected from decision making.^173,180^ Tools and indicators are fragmented by disease and funding source, with inadequate harmonisation and few national plans for coordination and data use.^180,181^ For example, 26 different bilateral, multilateral, governmental, and non-governmental organisations fund health information systems in Kenya, resulting in duplication of efforts and uneven distribution of resources within the country.^182^ 120 distinct digital health-related information systems operate in Tanzania.^173^ More than 1000 indicators are collected at the national level across the three major public health systems in Mexico, but only 27 overlap at least two health systems, preventing comparison and standardisation.

**Table 2:** Platforms for health system measurement

	Frequency	Level of collection	Relevant quality subdomains (Commission framework)	Best uses in measuring high-quality health systems
Administrative data (eg, HMIS)	Routine	Individual level data aggregated by condition within facilities and then by geographical unit	Population (care seeking), competent care and systems, and health outcomes	Monitor facility and clinician performance; monitor health status at the community and district level
Electronic health records	Routine	Individual patient	Population (care seeking), competent care and systems, and health outcomes	Inform clinical care; monitor facility and clinician performance; monitor health status at the community and district level
Population surveys	Periodic or continuous^*^	Population	Population (care seeking), user experience, selected health outcomes, confidence, and economic benefit	Represent both users and non-users of the health system; permit analysis of equity for subpopulations; have potential to be adapted for innovations in measurement, such as patient experience and patient-reported outcomes
Facility assessmentst	Periodic or continuous^*^	Health system	Workforce, tools; with observation or exit interviews: competent care, user experience, and confidence	Generate a representative assessment of health systems for subnational and national benchmarking; allow for assessment of user perspectives
Patient registries	Routine	Individual	Health outcomes, user experience, confidence	Monitor patient-reported experience and outcomes measurement over time
Vital and civic registries	Routine	Population	Health outcomes	Monitor population health status; form basis for policy guidance, projections, and planning

HMIS=health management information system. Commission framework is depicted in figure 1.

* Continuous household and facility survey methods that permit regular data synthesis, review, and health programme decision making have been proposed as an alternative to one-off surveys,^A83^ tested subnationally,^A84^ and adopted, for example by Peru since 2004, and by Senegal in 2012. tFacility assessments can include audits of structural inputs, interviews with health-care workers, direct observation of care, and exit interviews. References can be found in appendix 1.

*Panel 10:* From measurement to action*

Measurement alone will not ensure health system quality. Actionable information must reach agents capable and empowered to use it to effect change in the health system. Freedom of information—the right to access information held by public bodies—was enshrined in the 1948 Universal Declaration of Human Rights and has been adopted into law by more than 90 countries.^A64,A65^ Applied to health systems, freedom of information demands transparency of data within the system and to the public.^A66,A67^ High-quality health systems are not automatically produced by governments. A regulatory system that engages an array of actors should hold the system to account for high-quality care. This system includes formal mechanisms such as audits, ombudsmen, and courts and informal actors such as patients, the press, professional organisations, and civil society.^A67,A68^

A range of barriers can inhibit the flow of information about health systems. Power differentials can stymie communication, restricting the transmission of and responsiveness to local knowledge;^A69–A71^ hierarchical norms and fear of reprisal can inhibit incident reporting about health-care failures;^A72^ and, ironically, a surfeit of indicators in routine measurement systems can prevent the ready understanding and use of locally relevant information.^A69,A72–A74^ Although governments often claim to want to reach users through open government initiatives, scant attention to how people understand and use information has led to an abundance of information but a minimal effect on care seeking and other outcomes.^A75–A77^

Countries have the opportunity to take better advantage of increasing health system data by building trust in data, promoting learning cultures within the health system, and ensuring freedom of information. Obligatory reporting with data audit trails or data quality assurance institutions can bolster confidence in the indicators generated.^A74,A79^ A culture of information and learning within and across health facilities can lead to greater transparency and action.^A69,A80^ For instance, facility audits and licensing exercises should include clear criteria for improvement and result in non-punitive responses, such as support for addressing deficiencies.^A81,A82^ To ensure freedom of information, formal protection for whistle-blowers is an important guarantee, although a culture of secrecy and professional protectionism should also be addressed.^A72^ The free operation of traditional and social media can provide external accountability levers.^A67,A69^ Open government initiatives are an initial step, but their success should be judged on the basis of information use, not on quantity of data released. One path to fulfilling these opportunities is the development of a national body for monitoring health system quality, informing the public, and identifying and responding to failures, to serve as a locus for measurement, accountability, and action.

*Panel references can be found in appendix 1.

The proliferation of indicators burdens health-care workers and systems. In sub-Saharan Africa, an estimated one-third of health-care providers’ time is spent on recording and reporting.^173^ Health facility assessments cost a minimum of $100 000 per national survey and typically many times that amount,^183^ but are rarely used for national planning. Furthermore, fragmentation of these and other data sources prevents the coherent assessment of health system performance, to say nothing of actions in response to the data.

To understand how well this plethora of tools measures health system quality, we analysed multicountry health system indicator sets or surveys and sample national indicator sets from LMICs against this Commission’s quality framework (figure 1; appendix 1). Quality frameworks do not imply a need for equal measurement of each subdomain for all health services and conditions, but they do make apparent the multiple aspects of quality and highlight duplication and gaps.

**Figure 12:**
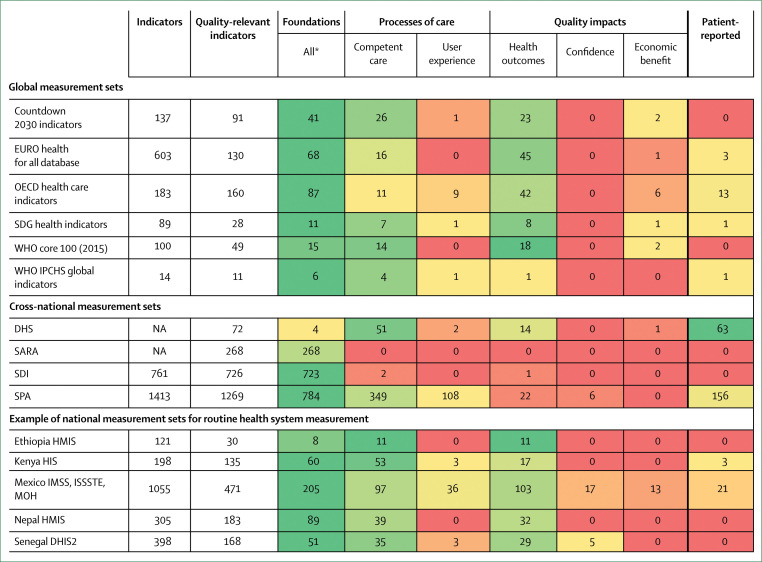
Representation of quality subdomains in global, crossnational, and national measurement sets We mapped indicators against domains of the high-quality health systems framework (figure 1), identifying the single domain most relevant for each indicator. We additionally classified indicators as patient-reported if the data were collected with individual self-reports. Full methods are detailed in appendix 1. Cells are coloured by greatest number of indicators per row (source), with red indicating 0, orange and yellow the midrange, and green the maximum number observed for that measurement set. DHIS2=District Health Information System 2. DHS=Demographic and Health Surveys. HIS=Health Information System. HMIS=Health Management Information System. IMSS=Instituto Mexicano del Seguro Social. IPCHS=Integrated People-Centred Health System. ISSSTE=Instituto de Seguridad y Servicios Sociales de los Trabajadores del Estado. OECD=Organisation for Economic Co-operation and Development. SARA=Service Availability and Readiness Assessment. SDG=Sustainable Development Goals. SDI=Service Delivery Indicator Survey—health. SPA=Service Provision Assessment. * Population, governance, platforms, workforce, and tools.

Measurement sets focused on the foundations of care, with global sets devoting 47% of indicators to this domain, crossnational sets devoting 70%, and national sets devoting 44% (figure 12). Inputs, such as tools and workforce, were the most commonly assessed subdomains and formed the entirety or bulk of the Service Availability and Readiness Assessment (SARA), Service Delivery Indicators, and Service Provision Assessments; our findings were consistent with existing research on the predominance of input measures in health system survey tools.^184^ All assessed sets, except SARA, addressed competent care processes, particularly care delivered, such as oral rehydration solution for children with diarrhoea. Although global and national measurement sets included population health outcomes such as neonatal mortality rate, user experience and non-health effects were sparsely measured across all sets.

The extensive collection of input measures is problematic. When collected through surveys, input data are quickly out of date and thus lose usefulness for supply planning. Moreover, our analysis found that readiness metrics are only weakly connected to the content of care delivered.^35^ Although the outcome indicators identified in this analysis are valuable for monitoring population health, we found few health-system-sensitive outcomes and almost no patient-reported outcomes.

The remaining measures in global sets pertained to competent care and, to a lesser extent, systems. Much of this measurement is focused on a subset of conditions, mainly maternal and child health and infectious diseases. Even in these areas, the validity of indicators collected raised concerns: for example, household surveys are not well suited for identifying children who truly have pneumonia to estimate appropriate treatment, and maternal morbidity and mortality in hospitals greatly exceeded the estimated rates based on documented administration of essential interventions.^100,185^ The validity of tools measuring clinical care is discussed in appendix 2.

In summary, the available measures do not promote accountability for high-quality health systems. Globally funded facility surveys overmeasure inputs that provide inadequate value for accountability. At the national and global levels, health system measurement is insufficient to assess performance of the health system as a whole and inadequate for holding the system accountable to people for the user experience provided or the effect on impacts—health and non-health—that matter to patients.

### Data quality

Data must be of adequate quality to be used for accountability or action.^186^ Efforts in the past few years have identified dimensions of data quality such as completeness and timeliness, internal consistency, external consistency, and external comparisons, although assessment tools focus mainly on completeness and accuracy.^186^

Routine health information systems, whether individual-level electronic health records or aggregate reporting such as the District Health Information System (DHIS) 2, provide information on the use and content of care that, if the data are of adequate quality, should form a crucial element of health system measurement for accountability.^187^ 34 LMICs—chiefly upper-middle-income, but including 13 low-income and lower-middle-income countries—had adopted national electronic health records systems by 2015·^188^ 41 LMICs, including 23 low-income countries, use DHIS2 at a national scale for aggregate reporting from electronic or paper registers in facilities.^189^ Notably, private sector facilities can be included under national health management information systems, although their participation and data completion are often low.^148^

Barriers to robust implementation and use of electronic health records and DHIS include restricted ownership by end users, scarce training on data skills, lack of motivation and engagement by overburdened health workers, large numbers of indicators required, and inadequate functionality of electronic platforms.^181,190^ As a result, data quality in routine health information systems is poor, with vertical programme assessments often identifying high prevalence of missing or inaccurate data.^181,191^ New evidence from Kenya, Nigeria, and Mexico suggests that such deficiencies in data quality also pertain to indicators of health system quality (appendix 2).

### Moving forward: three opportunities to measure better

#### Opportunity 1: Measure effective coverage

**Figure 13:**
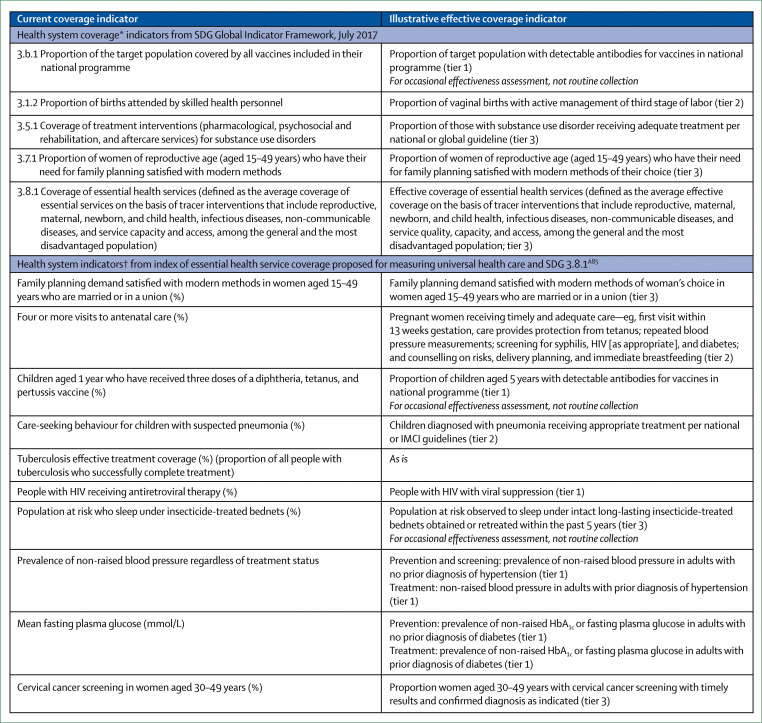
Illustrative indicators for advancing Sustainable Development Goal (SDG) monitoring from coverage towards effective coverage Tier 1=priority action is implementation (routine or targeted, as for immunisation). Tier 2=priority action is determining efficiency in indicators and data collection. Tier 3=priority action is development of valid indicators for use at scale. IMCI=Integrated Management of Childhood Illness. *Excludes health indicators focusing on population outcomes alone. †Six indicators not shown: two primarily measuring determinants outside the health system (tobacco use and access to basic household sanitation) and four service capacity and access indicators. References can be found in appendix 1.

Countries should incorporate measures of quality within a broader health system assessment to appropriately track the value of the health system. The geographic availability of facilities overstates health system performance: reduced mortality due to acute abdominal conditions was associated with proximity to well resourced hospitals in India, but not with access to lower-quality hospitals.^192^ New analysis suggests that this relation also occurs for obstetric conditions, acute surgical conditions, and time-critical adult infections in India, but less certainly for myocardial infarction (appendix 2). Even basic process indicators provide greater insight into hospital capacity than the availability of a facility or equipment. Service coverage monitoring that does not explicitly include quality will similarly overestimate health system performance and will do so substantially in many cases because of quality deficits. Achieving UHC requires effective coverage, such that “people who need health services obtain them in a timely manner and at a level of quality necessary to obtain the desired effect and potential health gains.”^193^ The current monitoring of UHC does not reflect this. Figure 13 lists the current coverage indicators for monitoring UHC specifically and the health-related SDGs more broadly. Only one of these indicators (effective treatment coverage for tuberculosis) captures the health system effect on population outcomes. Calculating effective coverage requires defining the population in need, access to care, and receipt of quality care.^194^ In figure 13 we also provide illustrative effective coverage indicators to suggest directions for future monitoring, and indicators for additional conditions are in appendix 2. Research is ongoing to identify standard indicators for many SDG conditions. Some indicators are available but need to be better implemented (eg, HIV), others need to be refined by selecting the best indicators and determining efficient methods of collection (eg, maternal health), and others still need to be developed de novo or validated for use at scale in low-resource contexts (eg, substance use).

Care cascades are an extension of the concept of effective coverage: instead of a single number, cascades break performance along the continuum of care to allow analysis of health system function.^195^ Cascade steps typically follow a patient population from health need through diagnosis, timely treatment, disease control, wellbeing, and survival. With each step conditional on the previous one, cascades illustrate health system failures in functions such as diagnosis, retention, and evidence-based care, while linking system performance to patient outcomes. Although specific indicators can vary across conditions (for example, disease control could be measured by viral load for HIV, blood pressure for hypertension, symptom-free days for major depressive disorder, and years without recurrence for breast cancer), the drop-offs in a disease-specific cascade can illustrate system-wide deficits: low rates of screening suggest failures in primary care as a first contact service, whereas poor outcomes among those on treatment implicate inadequate coordinated and continuous care. We provide examples and discussion in appendix 2.

#### Opportunity 2: Fewer, better metrics

For effective measurement of accountability and action, health system assessments must be reoriented away from measures that are poorly fit for purpose and towards people. A people-centred measurement means thinking about individuals across the life course and the total sum of their health system experiences rather than discrete services.^196^

*Panel 11:* Innovation in patient experience and outcome measurement*

The examples in this panel describe proof-of-principle testing of patient-reported indicators in low-income and middle-income countries. Shared investment, innovation, and learning will be needed to validate and define the use at scale of patient-reported measures for action and accountability.

**Measuring maternal care experience: companion of choice**

The Quality of Care Network for maternal and newborn health is leading efforts to standardise measures of childbirth care experience. Labour companion of choice is one of the quality measures for emotional support and is recommended in four WHO guidelines to date.^A86,A87^ Evidence shows that women who received continuous labour support might be more likely to give birth vaginally, be satisfied with their birth experience, and be less likely to have caesarean birth or use pain medication.^A88^ Labour companions can also play a role in the prevention of mistreatment of the woman during childbirth by serving as an advocate, witness, and safeguard. A process indicator would be the proportion of women who wanted and had a companion supporting them during labour, childbirth, and immediate post-partum period in a health facility, based on observation or facility or population survey. Currently, nine countries in the network are in the process of including and testing different mechanisms for three common experience of care indicators (including labour companion) as part of large-scale quality improvement efforts for maternal and newborn health.^A89^

**Measuring patient-reported outcome measures (PROMs) for pregnancy and childbirth in Nairobi, Kenya**

The objective was to understand the application of value-based health-care principles in a low-resource setting; specifically, to test a model for collecting PROMs in pregnancy and childbirth in a low-resource setting, to determine feasibility and scalability of using mobile platforms to measure PROMs, and to identify how to engage patients in collecting PROMs and motivate health-care providers to measure outcomes.

Outcome variables to pilot were selected from the pregnancy and childbirth standard set of the International Consortium for Health Outcomes Measurement, on the basis of importance, feasibility, acceptability (cultural and social), and literacy. Patient-reported outcomes included health status (incontinence, pain with intercourse), breastfeeding (success with breastfeeding), mental health (ante-partum or postpartum depression), and satisfaction with care during pregnancy, labour, and after birth.

Five facilities providing antenatal, delivery, and postnatal care services were involved and patient liaison officers were trained to support patient enrolment, maintain engagement, and oversee follow-up. Real-time collection of medical and financial data was done with M-TIBA, a mobile health wallet that tracks patients through the health system. PROM items were administered using text messages through mSurvey. 173 of 200 women enrolled, with survey completion rates near 90% through 6 weeks post delivery. See appendix 1 for full methods.

Sources: Özge Tunçalp and Meghan Bohren; Ishtar Al-Shammari and David Ljungman. *Panel references can be found in appendix 1.

Processes of care and quality impacts must be better measured to have health systems that are truly for people, with three areas for improved measurement: positive user experience, patient-reported outcomes, and non-health effects of care. OECD countries are moving towards standard crosscutting measures of patient experience, particularly communication and patient voice.^175^ Wide adaptation and validation of these measures would enable global comparisons. Other areas of user focus and respect pose more challenges for measurement, such as dignity, privacy, and non-discrimination. Vertical programmes with long experience in measuring such domains, including family planning, maternal care, and HIV care, can offer insight.^9,197,198^

Similar efforts to make patient-reported outcome measures more broadly useful are underway, including a focus of the OECD on population outcome measures such as quality of life.^175^ The International Consortium for Health Outcomes Measurement released a standard set of outcomes (including patient-reported outcomes) for hypertension, with an explicit focus on LMICs.^199^ Standard sets of patient-reported outcome measures for general adult and paediatric health are in development. The enhanced use of these measures will require clarity about minimum supporting data, such as risk factors.^200^ Panel 11 highlights efforts to adapt and apply patient-reported measures in LMICs.

Available measurements of confidence or trust in health systems fall short of the importance of this domain in shaping population behaviour and health outcomes. Satisfaction with health care or the health system is a commonly used measure and, from a legal and rights perspective, it reflects the ultimate judgment of the consumer.^201^ Satisfaction is associated with objective measures of process quality (eg, clinician competence) and with health outcomes (eg, mortality).^92,202^ However, satisfaction is also strongly influenced by a host of other factors, including user demographics and health, past care experiences, expectations, and potentially courtesy bias.^202^ This might explain some of the counterintuitive findings on user satisfaction. For example, satisfaction is often high for demonstrably poor-quality services, particularly for users with lower education or less experience with high-quality health care (panel 3). Conversely, people might express dissatisfaction when they expect, but do not receive services that are not indicated, such as antibiotics for the common cold. Improved health literacy can reduce this mismatch. Although user satisfaction gives an important perspective, other measures should be considered that might capture people’s confidence more directly. These could include trust in the health system, confidence that people can get the care they need, endorsement of the system as is (*vs* requiring major reform), and metrics that reveal preference, such as bypassing and loss to follow-up.^37^ The development and validation of measures for trust in the health system relevant for LMICs should be part of the global research agenda.

The links between health system quality and economic gains were detailed previously. The effects of health system quality on economic gains are largely mediated by health status (eg, incidence of surgical site infection or antibiotic-resistant disease and ability to function for work or school) and confidence in the health system. Measurement should focus on health and confidence themselves, while research quantifying links between these outcomes and economic impact is undertaken. Direct pathways include affordability that shapes individual costs of care and low system competence generating wasteful, unnecessary procedures. Measurement of cost has advanced notably in the SDG era: catastrophic out-of-pocket spending on health-care costs is the indicator for SDG 3.8.2, financial protection within UHC,^126,128^ and medical impoverishment provides an indication of how well financial protection for health services has been linked with poverty alleviation.^126,128^ Indicators of health system waste, such as excess caesarean sections, might signal poor system quality, although few measures have been defined for this with adequate benchmarks for national assessment in LMICs to date.

Measures of system competence are a key area for innovation, both in identification of essential indicators and in use of these to produce a coherent view of system function. Elements of system competence include safety, prevention and detection, continuity and integration, timely action, and population health management. Platforms within the health system—community outreach, primary care, hospital care, emergency medical services, and referral systems—can similarly be assessed for overall functionality. Work published in 2016 from the Institute for Healthcare Improvement^203^ proposed system measures for consideration in high-income settings. These measures include childhood immunisations, timely ambulatory care, preventable hospitalisations, hospital-acquired conditions, and serious reportable events (serious harm or death of a patient due to a healthcare error). In lower-income countries, consistent and accurate measurement of hospital mortality for selected services would be an important advance.^204^

One approach for system competence measurement is to consider conditions or procedures that require functional integration within a health-care platform and identify process or outcome measures therein as tracer indicators. For example, indicators such as blood transfusion delay, surgical site infection, and perioperative mortality rate provide insight into hospital care quality as a whole.^73,102,205^ Although perioperative mortality rates are collectable in countries of all income levels, virtually no LMICs have outcome surveillance in place. A focus on bellwether procedures and definition of standard methods in collection and reporting of both perioperative mortality rates and surgical site infections would reduce heterogeneity in measurements and facilitate their uptake into existing health system measurement.^73,104^ Similarly, timely trauma care is an indicator of prehospital care, such as emergency medical services and hospital functioning. Multiple studies have assessed time from injury to admission or admission to surgery, but measurement remains heterogeneous.^83^ Efforts to improve measures of system competence should include their potential use for accountability and triggering action.

#### Opportunity 3: Invest for country-led quality measurement

The current fragmented approach to health system measurement results in substantial efforts and investments expended for little data use.^176,206,207^ Progress on the measurement challenges and proposals described will require a shift to country-led quality measurement.^208^ This Commission calls on global, regional, and national donors to invest in national institutions for health system quality measurement. Such national bodies should be tasked with assessing available measurement against national priorities for health system quality, refining the measurement toolkit to better address the full high-quality health system needs, creating an annual public dashboard of health system quality performance, and assisting with policy translation of the results.

Building such an institution or arrangement requires enriching human capacity at all levels of the health system and concentrating advanced capacity in data science at the national level. Without improved numeracy at local levels and data management capacity at district or subnational levels, data quality will not be sufficient to support the activities of the national institution. Building more advanced measurement capacity—including more masters-level and doctoral-level researchers—within such a national institution will be necessary to address the current challenges of health system measurement and future ones, as population health and health systems evolve. Investing in a central institution with the authority to translate a national policy on health system quality into priorities for measurement and to both centralise data and disseminate findings is crucial to make measurements responsive, relevant, and efficient, particularly for countries with increasingly decentralised health systems. Having a single source of knowledge of quality deficits can also provide a clear basis for accountability of system failures and patient safety lapses.^209^

A truly national view of health system quality requires measurement from the private sector. The exclusion of private providers restricts health system assessments, particularly in countries with substantial private sectors, such as India. For example, an analysis of population coverage of first-level hospitals in Karnataka state, India, found that 45% of the population had access to at least one public hospital within a 25 km catchment area, whereas 91% had access when private hospitals^210^ were included in the analysis (two-step floating catchment area method; appendix 2). Nonetheless, information on the capacity and quality of private facilities—or even their number and location—is scarce.^204^ Some health system assessments, such as the District Level Household Survey 4 in India, are restricted to public facilities, and routine health information systems can be compulsory for public providers only. A review done for this Commission identified multiple mechanisms for measuring private sector quality, including regulation, national information systems and surveys; purchaser-driven, consumer-driven, or network-driven measurement; and voluntary external assessments. Private-sector providers sometimes express a willingness to share data, but without strong mechanisms and incentives, little sharing occurs in practice.^211^ Future research on models for integrating data across the public and private sectors to enhance efficiency, transparency, and accountability is warranted.

The development of a national policy and strategy for health system quality is a prerequisite to country-led measurement and is discussed further in the next section.^212^ Assessing measurement approaches against the standards defined in the national strategy will provide insight on gaps and inefficiencies in measuring quality. Another responsibility of a national institution for health system measurement is the development of the quality measurement toolkit. The toolkit can differ by context and resource availability, but should include three tiers: foundational systems, routine data, and targeted studies. The first tier consists of vital registries to track population births and deaths, supply chain management, and human resources information systems, including provider payment tracking. These elements are fundamental for a sound understanding of the population and the capacity of the health system. The second tier is routine data collection through electronic health records or health information systems; many measures for effective coverage and system competence can be derived from routine health information systems. Accuracy and parsimony are essential to these measurements, because of not only their importance, but also their high potential burden. The third tier consists of targeted health system studies, which include health facility and population surveys and patient registries to probe more deeply into health needs and system performance. Facility assessments must be more agile and responsive to national priorities, with increased emphasis on measures that might be hard to capture in a routine system, including timeliness and accuracy of care delivery and patient experience. Patient registries can be developed as a subset of facility assessments to provide information on health outcomes and patient perspectives over time for priority groups or conditions.^213^ Population surveys, ideally linked to health facility assessments or routine health system data, can be broadened to address the range of conditions reflected in the SDG agenda and to provide the voice of users and non-users on their needs and outcomes. These surveys will continue to be instrumental in providing data for equity assessment, particularly in lower-income countries. When optimised, the combination of these data sources has powerful potential to advance the quality of health systems. The matrix of tools will differ by context, because one of the aims of the SDG era is for all countries to own their data systems and to define their data needs within a common framework. National ownership of tools at all tiers is important for the results to be integrated and used.^208^ Regional and global partners can facilitate and catalyse this work by providing public goods of centralised evidence and tools. These can include repositories for available indicators, evidence and guidance on the role of measurement platforms and methods for triangulating across them (eg, in effective coverage estimation), and tools for synthesising insight for dissemination. Regional collaborations might prove beneficial for sharing learning and avoiding duplication of efforts, particularly for small countries. Initiatives such as the Quality of Care Network and the Health Data Collaborative are important steps in this direction.

**Figure 14:**
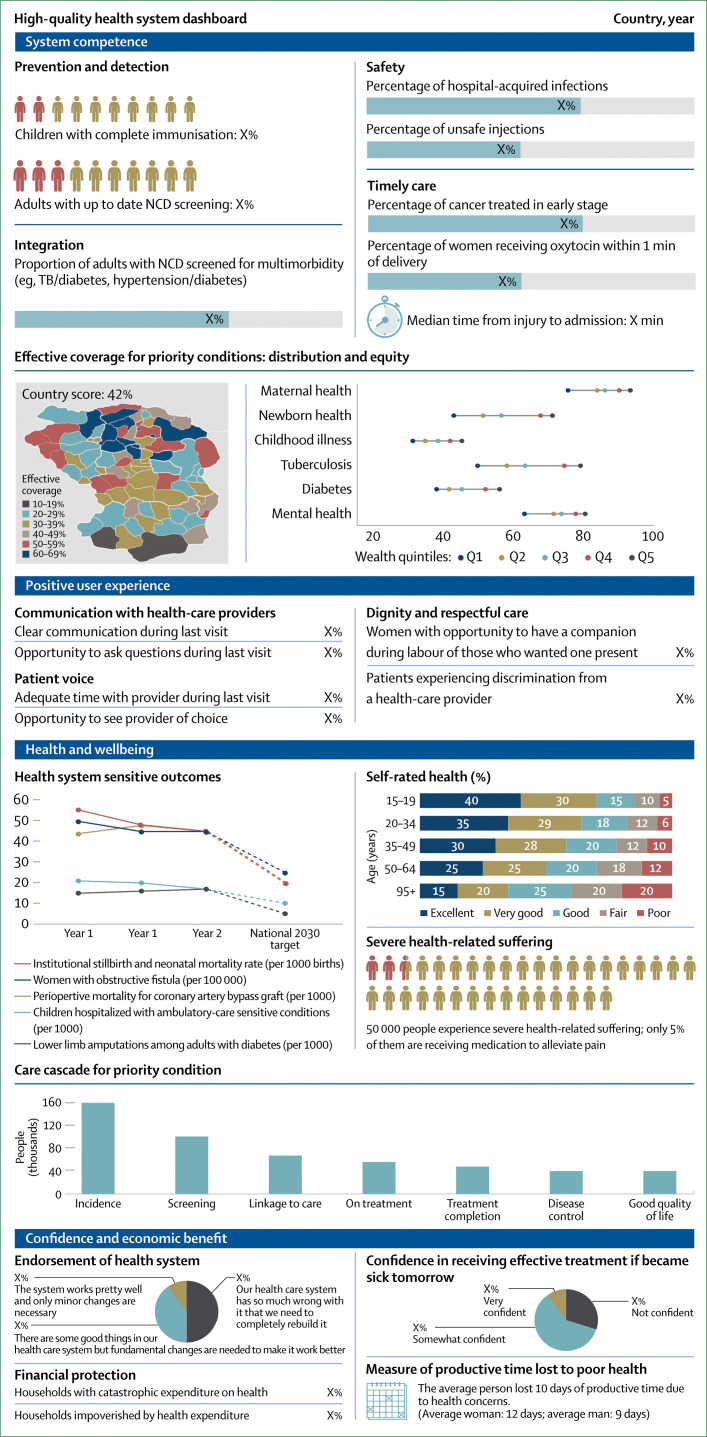
Sample high-quality health system dashboard with illustrative indicators

Finally, this Commission recommends that countries compile an open-access health system dashboard for monitoring progress towards a high-quality health system. The dashboard would track health system quality with use of data from multiple sources. The dashboard would be people-facing and should reflect what matters most to people: health and wellbeing, user experience, system competence, confidence, and economic benefit. An example dashboard is shown in figure 14, featuring these recommended areas and illustrative indicators of each domain to show how such information might be presented. Effective coverage indicators can signal areas of underperformance by geography, condition, or vulnerability, whereas care cascades for conditions that illustrate overall system functioning can be used to identify strengths and failure points. Indicators should be selected and adapted to each country as described previously. The dashboard should evolve to reflect changing health and health system priorities. Efforts are already under way to contribute elements to such a dashboard, from real-time views of staff absenteeism in facilities in India^214^ to open data platforms in Kenya.^215^ Providing information is not in and of itself sufficient; information must be accompanied by appropriate context for public consumption and clear mechanisms for engagement and response by all people, whether they are members of the public, the press, or health system actors, such as medical associations.^216^

Countries should present overall dashboard results and results disaggregated by subnational regions and dimensions of vulnerability (settings of care, disease type, or demographics, as discussed in Section 3), as well as results for public and private sectors. This Commission recommends that the dashboard be released from 2021 onwards. It could reflect gaps in data availability and quality particularly early in its usage, before measurement platforms are realigned to provide a full system perspective. Missing information should not prevent the public release of what is available as input into mechanisms of social accountability. Public release of health system quality information is an important way of building trust in health system transparency, in addition to providing means for self-scrutiny by health system agents.^217^ A high-quality health system dashboard is an essential step in a cycle of accountability and a trigger towards universal action for improvement.

## Section 5: Improving health systems at scale

The key findings of this section are shown in panel 12.

### Expanding the solution space

Despite some impressive health gains in LMICs in the past several decades, this Commission’s analysis showed that health systems are beset by poor-quality care. The pervasiveness of poor quality suggests that the cause is not a few weak providers or clinics, but rather that whole health systems are underperforming. To successfully address the endemic nature of poor-quality care and to give providers the right support to deliver the competent and respectful care that people deserve, this Commission calls for an ambitious improvement agenda that moves beyond targeting the manifestations of poor quality and aims to transform health systems.

**Figure 15:**
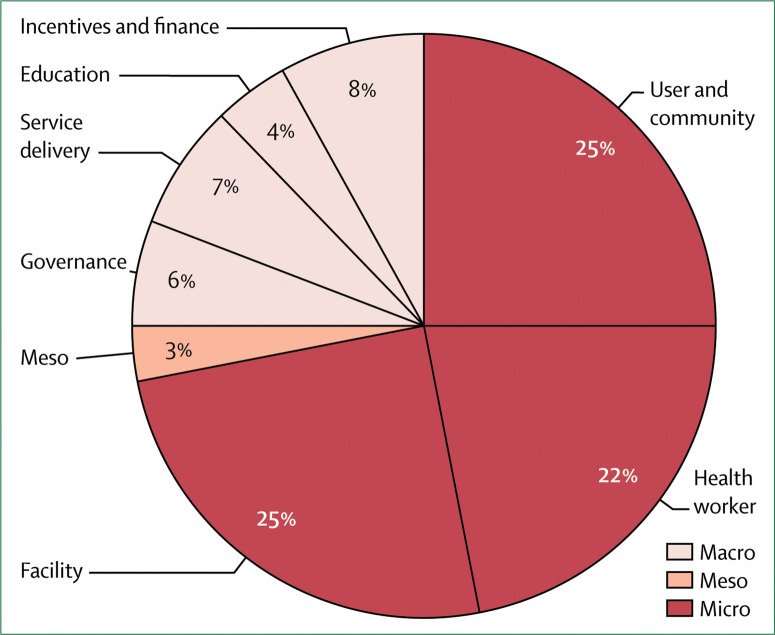
Types of interventions and levels targeted to improve quality of primary health care according to published literature from 2008 to 2017

However, strategies for quality improvement in LMICs have generally focused on a narrow set of solutions, such as increasing health system inputs and changing people’s behaviours and routines at the point of care—ie, the lowest (micro) level of the health system. A 2018 review of primary care quality found that, globally, 72% of strategies targeted the micro level (figure 15; appendix 1). Although interventions aimed directly at facilities and staff can be motivational and promote local commitment to quality,^218^ people tend to revert to entrenched ways of doing things, especially when surrounding systems do not support transformation.^23^ The application of multiple micro-level interventions might lead to deleterious effects, with interventions clashing at the point of care because implementing them consumes a large amount of attention from managers, potentially detracting from other priorities.^219,220^ This raises the challenge of how to situate micro-level efforts as part of broader reforms that will improve health systems.

*Panel 12:* Section 5 key findings

1. Addressing the quality deficits in many countries today will require expanding the solution space— the feasible set of solutions that satisfy the constraints of the problems—for improvement to include macro-level, meso-level, and micro-level interventions.
2. Countries should invest in the foundations of high-quality health systems by considering four universal actions: governing for quality, redesigning service delivery to maximise quality; transforming the health workforce to provide high-quality, respectful care; and igniting people’s demand for high-quality care.
3. Several commonly used approaches, such as accreditation and performance-based financing, have not been consistently effective in improving quality.
4. District-led collaborative learning has the potential to foster improved quality through better system functioning and communication, but more research on most effective models is needed.
5. Research on strategies to directly improve health worker and facility performance found that most micro-level solutions have modest effect sizes. Studies tend to be small and brief, limiting conclusions about sustainability and effects at scale. This Commission recommends that selected meso-level and micro-level interventions be implemented alongside efforts to improve the foundations of health systems.
6. Development partners should support health system reforms that improve the foundations of high-quality care.
7. Monitoring and evaluation of the impact of all improvement efforts at national and subnational level is needed to drive learning and improvement.

A transformative quality improvement agenda is based on the recognition that health systems are complex adaptive systems, defined as systems in which many component parts interact in unexpected ways and often produce unanticipated results.^221,222^ Complex adaptive systems are resistant to change, and diffuse and isolated interventions, especially at the micro-level, are unlikely to result in large-scale improvements.^221,223^ An example of this is the proliferation of point-of-care technologies for health, few of which have been taken to scale or shown to have had an effect on health in LMICs. At the same time, evidence^23^ from health and other sectors shows that complex adaptive systems can thrive if actors within the system have a shared vision, clear rules, and space to allow evolution and learning. Research^224^ in behavioural economics noted that successful systems create a choice architecture that supports intended goals and reduces harmful variation. Choice architecture comprises the elements of a system that influence choices and behaviour, including information flow, incentives, presentation of choices, and decision-making contexts.^224^ Nudging, or steering people in a particular direction while preserving their choice, is a common behavioural economics strategy, but the broader notion is to align motivations, incentives, oversight, and management across levels to promote the best actions.

We propose a new improvement approach that addresses the scope of the quality challenge and recognises the complex adaptive nature of health systems. This approach emphasises macro-level reforms—what we call universal actions—that can not only establish and cascade systemic change across all levels of the health system, but also include a role for targeted meso-level and micro-level strategies. Macro-level strategies are best able to directly tackle the social, political, economic, and organisational structures that shape a health system. Meso-level (subnational) interventions address quality of care through the coordination and management of a network of facilities and communities. Interventions at this level are also well positioned to improve communication and learning between facilities and across levels of the health system. Micro-level interventions aim to directly influence the performance of the staff or the operations of a facility. Appendix 2 includes examples of interventions at the three levels of the health system.

System-wide improvements in quality of care will require effort from providers, health system administrators, and communities, but they begin with a political commitment from heads of state and ministers. Global development partners can and should assist, but they should not drive this agenda. Contributions from across the health system, including the private sector, and from sectors outside of health will be crucial. Early gains in quality are likely to be visible within a few years, though meaningful improvement might take longer. People everywhere have a right to receive effective and respectful care—the time to get started is now.

### Universal actions for improving quality

**Figure 16:**
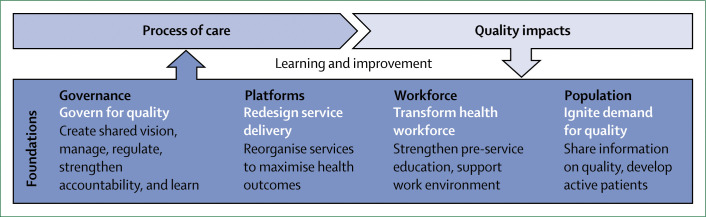
Universal actions for improving quality of care

This Commission recommends four universal actions to improve health system quality: governing for quality, redesigning service delivery to optimise quality, transforming the health workforce, and igniting people’s demand for quality (figure 16). These actions are based on successes and failures from all countries, best practices from high-performing health systems, research and evaluation, and the experience and deliberation of the Commissioners. This Commission sees these universal actions as the start of a paradigm shift towards a more ambitious health system improvement agenda. Beyond the universal actions, countries can select additional targeted opportunities that fit their needs and context. All universal and targeted actions are predicated on having adequate health system inputs, such as staff, medication, and equipment. The optimal composition, design, and implementation of the improvement agenda will vary by country, because approaches that work in one setting might not work in another. Countries need to monitor the implementation of this agenda to permit adaptation and assess the effects on health and other valued outcomes.

#### Universal action 1: Govern for quality

Health-system-wide change demands that the improvement and maintenance of quality be woven into the fabric of a health system. Governing for quality means reframing the pursuit of quality health care from a peripheral activity to the mandate of a health system, and making sure that a commitment to quality is actually translated from paper to actions that improve the health of people.^225,226^ Governing for quality includes several elements: adopting a national quality policy and strategy, improving capacity for management at all levels of the health system, strengthening regulation and accountability, and collecting and learning from health system data.

Governing for quality requires high-level political commitment to a shared vision for improving quality of care and translating this commitment into action across the health system (panel 13). Well aligned policies and strategies should be based on this vision, locally accepted definitions of quality, and national goals for improved outcomes.^212^ In response to requests from countries for guidance on how to design and implement these healthcare policies and strategies, WHO produced the National Quality Policy and Strategy Handbook.^212^ The handbook outlines eight elements of the strategy and argues that quality must be elevated nationally and become a priority across sectors. These policies, and the strategy linked to them, should ideally outline the roles and responsibilities of the organisational bodies and actors that participate in sustaining and improving quality of care. A plan for coordinating these elements is also needed, so that quality improvement programmes are harmonised to maximise learning and results at the system level.^227^ For example, an analysis^228^ of surveys from 310 health system leaders in Mexico identified insufficient coordination of quality improvement agendas and an unclear system of roles and responsibilities as key barriers to the translation of federal policies into improved quality of care. The successful development of shared vision, policies, strategy, coordination, and implementation are needed to design a choice architecture for health systems that directs patients and providers towards decisions that produce quality care and good health outcomes.

Improving the quality of the health system requires action from multiple sectors and stakeholders. Governing for quality includes managing these relationships and convening stakeholders under the shared vision of making large-scale sustainable improvements in quality and health outcomes.^229^ Inclusive processes that bring a diversity of voices together to solve problems are complex and difficult to manage, but they help to make action on quality possible, they foster innovation, and they lead to more comprehensive solutions.^230^ Building partnerships means aligning all stakeholders, including international donors, with national needs and priorities, which is a challenging goal. For example, in 2016, only 16% of development assistance for health went to the strengthening of health systems, despite evidence showing that condition-specific funding can compromise overall quality of health care and crowd out existing health services.^231–233^

Adopting a national quality policy and strategy, and engaging stakeholders around it, requires not only strong leadership skills, but also good management at all levels of the health system to effectively use available resources to realise the vision of high-quality health care.^234^ Middle management at the district or regional level could play an important role at the intersection of policy and implementation, although management capacity interventions at all levels have been linked to better health sector performance.^235,236^ Although the literature consistently points towards the importance of good management across health system levels, insufficient attention has been paid to creating the capacity for health-care management in LMICs.^235,236^ Data from multiple LMIC settings showed that management is a key factor that differentiates between high-performing and low-performing facilities.^237,238^ Bradley and colleagues^235^ outlined eight key management competencies and recommended designing training programmes for management professionals to achieve them. These key management competencies are: strategic thinking and problem solving, human resource management, financial management, operations management, performance management and accountability, governance and leadership, political analysis and dialogue, and community and user assessment and engagement. Examples of effective training programmes^239,240^ exist in various settings, including Ethiopia,^239^ where hospital performance improved under the management of graduates of the Masters in hospital and health care administration programme.

*Panel 13:* Governing for quality: lessons from Nepal and Argentina*

**Absence of multistakeholder commitment leads to minimal quality improvement in Nepal**

In 2007, Nepal endorsed the Policy on Quality Assurance in Health-care Services, with the objective of ensuring “quality of services provided by governmental, non-governmental, and private sector according to set standards” and to establish an “autonomous body to ensure impartial decision regarding health services.” 11 years later, the success of this policy remains mixed.

Why did the policy have low impact? The policy was created without a shared vision and buy-in from stakeholders, including the Ministry of Health. Important partners, such as the Ministry of Education, did not provide critical inputs. The policy designers also did not create consensus on a definition of quality or agree on indicators against which to measure progress. A centrepiece of the policy—to establish an autonomous body for quality of care— never materialised. A quality assurance section was established in the Department of Health Services, but it has little leverage over other units in the Ministry.

The absence of a political commitment and involvement of all stakeholders has meant that the objectives of the Policy on Quality Assurance in Health-care Services have been largely unrealised, and health institutions continue to deliver subpar care quality.^A90^

**Governing for quality through strong accountability in Argentina**

In 2005, Argentina implemented a public supplementary insurance program, SUMAR, designed to increase access to quality health care for uninsured children and pregnant women and to address large disparities in infant and maternal mortality rates.^A91,A92^ The programme is credited with decreasing the probability of low birthweight among beneficiaries by 19%.^A91^

In the setting of Argentina’s national decentralised health system, SUMAR’s success was dependent on high-level political commitments, buy-in from provincial governments, and well designed reporting pathways to ensure accountability. A presidential decree established the programme and provincial governments confirmed it under a collaborative agreement with their respective providers. The agreement is renewed yearly with review of procedures for expenditures and goals to be achieved. Federal commitments and provincial implementations were aligned through clear standards, and multidisciplinary oversight bodies monitored performance. A provincial level programmatic office regularly reported to the federal level. Local accountability was increased through the centralised monitoring of transferred funds to the provinces. The provinces were then responsible for enrolling beneficiaries, organising the provision of services, and paying providers.

Source: Amit Aryal and Franziska Fuerst.

Source: Programme SUMAR, Argentina. *Panel references can be found in appendix 1.

To improve and guarantee quality care, good leadership and management competences must be buttressed by regulatory structures that create accountability. Strong regulatory mechanisms, ie, so-called regulation with teeth—and transparency through good monitoring, measurement, and reporting practices—support accountability both internally within the health sector and externally with civil society and citizens.^225,241^ The accountability mechanisms, in turn, should be operated by leadership and management that can pull together a complex array of regulatory domains (eg, workforce, facilities, products, and service delivery) that might be administered by multiple institutions. Lessons from the regulation of medicines suggest that multipronged collaborative approaches that include a suite of regulations, mechanisms for legal redress, and training of inspectors in the public and private sector are most likely to be effective in mixed health systems.^242^ These accountability mechanisms should also include monitoring of the flow of providers between private and public practice.^243^ Two first steps that are yet to be taken in many LMICs are gathering accurate descriptive data about private health care (see Section 4) and maintaining the capacity for ongoing monitoring. Local regulations that apply to private health care vary considerably and need to be explored in detail. Finally, regulatory bodies that can enforce compliance across public and private sector institutions are often severely under-resourced, do not have basic capacity, and will need to be strengthened.^244^

Governing for quality also means recognising the importance of, and making space for, civil society in regulating the quality of care. Professional organisations that regulate their members have an important role to play in health system quality by promoting high-quality performance of their members and by sanctioning them when they fail to meet minimum standards. Self-regulation is underused in LMICs, where professional organisations mainly advocate for their membership. Experience in high-income health systems has shown that the privilege and responsibility of self-regulation promotes professionalism, the sense of accountability among professionals to people, and reduces transaction costs for governments. For example, in Canada,^245^ physicians successfully self-govern all aspects of the profession, from setting nationally uniform entrance exams to monitoring and remediating substandard clinical practice among practising physicians. However, self-regulation is not without its challenges, as exemplified by the UK,^246^ which has moved towards joint government–professional oversight because of a series of widely publicised physician scandals. When professional groups have primary fiduciary responsibility, care should be taken to involve both practising clinicians and citizens in governance and to avoid unnecessary fragmentation of regulatory responsibilities.^247^ Professional organisations can also promote quality through continuing medical education and engaging directly with governments to address quality concerns. For example, the Philippine Medical Association has more than a century of experience in agitating for improvements in medical education, health facility infrastructure, and the regulation of pharmaceuticals.^248^

Social participation in health care, especially for the most marginalised, has intrinsic value as a human right and instrumental value in improving health care and keeping systems accountable.^249^ People and communities are experts in their local experience and, with skilled support, can wield this knowledge to help create highly valued solutions to health-care problems.^250,251^ Social participation can also increase the uptake and sustainability of services.^252,253^ Although the composition of civil society varies by country, it is their diversity of perspectives, the opportunities for participation and action, and the availability of accurate and understandable information that will make this sector effective in holding governments accountable for high-quality health care.^243,249,252,253^ Civil society can be particularly powerful when adopting a human rights framework for advocacy.^253^ For example, in Uganda,^254^ the Center for Health, Human Rights, and Development regularly uses legal avenues to challenge policy makers on issues such as essential medicines, safe and respectful maternity care, and fair treatment of patients with disabilities.

Institutional accreditation uses external evaluators to assess facility performance against health-care standards. Although frequently cited as a quality accountability mechanism, a scoping review of reviews done by this Commission found that the direct effect of institutional accreditation on quality of care is uncertain (appendix 1). In a systematic review of improvement strategies, median effect sizes for institutional accreditation were modest: 7·1 percentage point improvements in quality outcomes were reported (appendix 1).^255^ However, accreditation can indirectly affect quality through improved management, professional development, and capacity of facilities to promote change.^256^

Improvement entails the continuous production of relevant data, which measures performance and outcomes, and the translation of those data into action—a learning system.^226,257^ This learning system facilitates the development of programmes and reforms based on the best available evidence (whether global, regional, or local data) and best practices. New initiatives should embed measurement, evaluation, and plans for how the results could be disseminated effectively to the people responsible for ongoing data use to inform adaptation of services. Learning systems should also identify best performers, as discussed in Section 2, and determine the basis for their success.

This set of intentional processes for actively learning and improving the health system is a goal that should be articulated and demonstrated first by the actions of senior leadership and subsequently echoed by middle management and the front-line staff. This system goal should become the primary guiding principle that creates the motivation for system improvement over time and for which health system actors hold themselves accountable.^258^ Planners should design better systems on the basis of lessons learned and then link back to system managers, supervisors, and front-line staff to support improvement. Developing well functioning learning systems is especially important because of the imperfect evidence base for quality improvement interventions and the large variation in effect sizes found between studies and contexts. Learning systems ensure that planners can make course corrections based on context-specific data. A meso-level strategy that illustrates this approach is the quality improvement collaborative, which we describe in the following subsection.

An analysis done by this Commission regarding five country experiences on governing for quality revealed practical lessons for operationalising the described principles (methods are described in appendix 1). District and facility-level health workers might be unaware of national quality policies and strategies or might not understand the implications of those on their daily work. The dissemination and translation of policies and strategies needs to be formally assigned, built into the job descriptions of public sector administrators, and included in performance reviews of these individuals. Additionally, the workforce might experience distracting and overwhelming policy crowding, with poorly coordinated and sometimes conflicting mandates. Countries are encouraged to review all policies affecting front-line workers; overlapping or conflicting policies can then be pruned, leaving a policy set that is coherent from the perspective of the service provider. For example, a nurse in primary care seeing a patient with diabetes and latent tuberculosis would benefit from having a single quality policy, not separate documents on diabetes and tuberculosis.

Informants from all levels of the health system discussed the challenges of good system-wide data use in the Commission analysis. Data generation and translation must start at the local level, but for system-wide improvements to occur, these data need to be coordinated centrally. We suggest the creation of planned spaces for information exchange, such as district-led meetings to learn from the evidence generated. Success stories of improvements made possible by accurate data collection and skilled data translation can be shared with front-line health workers to motivate continued quality care and improvement.

#### Universal action 2: Redesign service delivery to optimise quality

Most LMIC health systems were originally designed to provide basic episodic care, especially for infectious diseases. Many systems have not adapted to the changing landscape and challenges of caring for people with chronic diseases, mental health conditions, and more complex injuries and illnesses.^20^ Hospitals and healthcare facilities with advanced diagnostic and treatment capabilities are overcrowded with stable patients who could be treated in primary care facilities, whereas many first-level health clinics are expected to handle cases that are beyond their scope, with slow or non-functioning referral for emergencies.^259,260^ Poorly organised health systems lose lives, waste scarce resources, and squander the good will of populations.

To address this, this Commission calls for a quality-focused service delivery redesign: a reorganisation of services within the health system to efficiently maximise health outcomes and user confidence, rather than only geographic access to clinics. Service delivery redesign capitalises on existing health system assets to provide services at the appropriate level and achieve the highest quality of care possible.

First, some services should be shifted to primary care. Reflecting the core principles of continuity, coordination, comprehensiveness, and first contact, competent primary care is ideal for treatment of chronic and stable conditions that require sustained engagement with the health system (eg, non-communicable diseases and stable HIV or tuberculosis infection), preventive care (eg, immunisation, antenatal or routine child care, and growth monitoring), and low acuity and algorithmic services (eg, care of minor child and adult illnesses and injuries).^20,36^ Palliative care can also be expertly delivered close to home by primary care and in partnership with families, community caregivers, and spiritual supporters.^36^

Examples of the partial implementation of quality-focused service delivery in LMICs reveal the benefits of shifting these services to primary levels. In HIV care, stable patients are managed in primary care clinics with impressive results, and new patients can initiate treatment in their own communities.^261^ As a result, centralised specialty centres are less crowded, allowing higher-skilled providers to focus on more complicated cases, such as HIV treatment failures.^260^ A multicountry meta-analysis^262^ of 39 090 patients with HIV showed that patients in primary care were half as likely to be lost to follow-up than patients treated at a centralised HIV clinic. In tuberculosis care, community-based models are also substantially less costly to implement.^263^ Uncomplicated non-communicable diseases are especially well suited for care at the primary level, where providers can more effectively monitor chronic disease over time and build relationships that form the foundation for effective communication and counselling regarding crucial lifestyle modifications.^20^ An important caveat is that current primary care models in many LMICs are outdated and ill-suited for these new tasks. New thinking is needed on primary care functions, capacities, and connections with specialised services, especially in urban settings.^20,264^ For example, experience from high-income settings suggests that non-visit care, in the form of virtual or phone visits, has the potential to extend the reach of primary care for low-acuity conditions.^265^

Acute or chronic conditions with higher risk of mortality or severe morbidity are best assessed at a hospital with emergency capacity. The correct health system level for some surgeries should be determined on the basis of availability of specific technical skills, laboratory, imaging, and intensive care infrastructure, acuity of the condition and projected procedure volume. Complex or rare conditions are ideally managed in tertiary, highly specialised, care centres.

Childbirth is one situation that benefits from care at hospitals with surgical and specialised newborn care services, because complications can arise without warning and require rapid, highly skilled care.^266^ However, in low-income countries, a substantial proportion of obstetric and newborn care is provided in primary care facilities without adequate expertise or surgical capacity.^267^ For women and newborn babies who develop complications in primary care clinics, poorly functioning referral and transport to a higher level facility mean a much greater risk of morbidity and mortality.^267,268^ Guided by this logic, many high-income and middle-income countries mandate that all women deliver in, or next to, hospitals with surgical and advanced newborn care services.^269^ The structural deficits in highly skilled health workers and surgery at primary care levels might explain why the Better Birth trial,^39,270^ a large randomised controlled study, found that implementing a safe childbirth checklist and coaching for nurses and midwives at primary care centres in India did not reduce maternal and newborn morbidity or mortality.

We examined the practical implications of shifting delivery care to hospitals in a geographic modelling that linked facilities with pregnant women in six LMICs (Malawi, Haiti, Tanzania, Kenya, Namibia, and Nepal; methods are described in appendix 1). We found that delivery care redesign would result in substantial gains in technical quality for care of pregnant women without reducing interpersonal quality and with minimal reductions in 2 h access to care. For example, in Tanzania, hospitals score twice as high as primary care facilities on a basic measure of childbirth quality and, therefore, quality of care would improve by moving all deliveries to hospitals. Although this would increase the average distance from a delivery facility for rural dwellers, only 27% of pregnant women would live more than 2 h away from a delivery facility in Tanzania, compared with a current 17%. In the remaining countries, 1% to 7% of women lost 2 h access to care. This redesign can also produce efficiency gains because resources could be redirected from providing obstetric care in thousands of facilities to improving quality in fewer hospitals, promoting care integration across facilities, working with communities, and enabling transport to hospitals.

Strong interfacility communication and referral networks are crucial to the success of quality-focused redesign, along with investments and participation from non-health-care sectors. Tools to facilitate redesign that warrant consideration include improved transportation (eg, community taxi services and ambulances),^271^ communication (district-led learning, discussed in the following subsection), measures to reduce access barriers to high-quality facilities (eg, vouchers and maternity waiting homes),^272,273^ and public education to enhance population understanding of the right place for care.^274^ Local context, with a focus on facilitating access to high-quality care for the most marginalised subpopulations, should drive the mix of interventions and incentives.

Planning for quality-focused service delivery redesign in any country would require analyses of patient volumes, bed and surgical capacity, provider competence in existing hospital facilities, and potential upgrades to existing health-care centres to permit high-quality care, as well as attention to transport, costs, and building community demand.^275^

#### Universal action 3: Transform the health workforce

The data in Section 2 showed that providers often do less than half of recommended evidence-based care measures and that rates of diagnostic accuracy are low across health conditions and countries. A Commission analysis showed that this is also true of providers in their first 3 years of practice, suggesting a probable role of poor preservice education in provider performance (appendix 1).^276^ Low knowledge and competence of the health workforce is at risk of worsening over the coming years because of the rapid expansion of health workforce training institutions, resulting in dilution of already insufficient faculty and curricular resources.^277,278^ Despite this threat to health-care quality in LMICs, improving the education of health-care professionals has not been a central part of the improvement discourse.^279^ In the previously mentioned review of primary care quality improvement, only 16 of 379 articles addressed the preservice education of health professionals (figure 15). Fixing these gaps through in-service training is not an effective antidote,^280^ and reforms in professional education are required to adequately equip these professionals to provide high-quality care.

*The Lancet* commissions^277,281^ on health professionals for a new century and on the future of health in sub-Saharan Africa highlighted key steps to address the quality gap of the health-care workforce. First, the education of health professionals should focus on achieving competence through active learning, early clinical exposure, and problem-based learning. Competency should be defined by the gaps and needs of each individual country and include domains beyond the technical skills of providers. Ethical, respectful, and compassionate care, and the fundamentals of systems thinking and quality improvement should be additional core competencies. Dysfunctional systems will continue unless the workforce is prepared to improve them.

Second, the chronic understaffing of many health-care professional schools in LMICs must be addressed, along with support of high-quality teaching, for the quality of clinical education to improve.^278^ Possible solutions include increasing salaries, expanding professional development opportunities, using state policy levers to require practising clinicians to teach trainees, and providing small incentives, such as free housing or telecommunications.^278^ Finally, health education institutions should establish student recruitment and retention policies to increase the representativeness of the student population.^252,282,283^ Evidence has shown that care interactions between providers and patients who are racially, culturally, ethnically, or linguistically similar are associated with higher perceived quality of care, satisfaction, and improved medical communication.^284,285^ These changes within institutions of higher learning must be supported by good governance and quality-informed policy making. Intersectoral coordination between ministries of health and education would create a more direct link between the production of a health workforce and the needs of the health system.^281^

Third, health-care providers also need a work environment in which they can succeed beyond graduation. Many health-care providers face challenging conditions, including inadequate and delayed salaries, heavy workloads, ambiguous responsibilities, no opportunities for growth, and poor treatment by colleagues and patients.^276,286,287^ Not only do these conditions result in burnout, mental distress, and poor retention for providers, but they also result in poorer quality care.^287–289^ Motivated providers are less likely to make poor decisions or medical errors and are more likely to be empathic towards patients.^290^ Good working conditions, regular pay, clinical support, and opportunities to learn and grow are essential to maintain a workforce that is motivated and committed to providing high-quality care.^286,291,292^

WHO recommended a set^293^ of decent employment policies to support providers, including ensuring occupational health and safety, fair terms for workers, merit-based career development, and a positive practice environment. In addition to broader policies, a review^294^ published in 2017 recommended a set of steps for facilities to foster joy and engagement in their own workforce. These include an initial process of inquiry to understand workforce priorities, followed by identifying and removing the primary annoyances, initiating simple fixes, and using improvement science methods to spur larger-scale change to create a fundamentally more satisfying and happier work environment. Although early reports suggest that sense of purpose can be strengthened through these approaches, much of this work has started in the past few years and the effectiveness of these interventions on improving quality of care in LMICs remains to be determined.

#### Universal action 4: Ignite population demand for high-quality care

High-quality health systems respond to people’s expectations, but if those expectations have been dampened by a history of disempowerment and poor-quality care, that response will not translate into better health care.^295^ Section 2 shows that when expectations are low, quality ratings of objectively poor care are high. This discrepancy lets health systems disregard issues of quality. Beyond putting pressure on systems to improve, generating demand for quality through information sharing would increase health system accountability (see universal action 1) and has an ethical foundation: for patients to be autonomous decision makers, they must have access to usable information about the quality of their care.^296^ This is imperative because of the information and power asymmetry that exists between patients and providers. Finally, this Commission’s recommendation is based on evidence that people who already demand higher quality in LMICs and actively make decisions can extract higher quality care from their health systems.^118,119,297,298^ National quality improvement strategists are encouraged to explore demand-side approaches that raise people’s expectations of quality.

Very few improvement programmes are explicitly designed to raise demand for quality care. We used those few programmes to draw lessons on this understudied improvement opportunity. Participatory women’s groups are a well documented^299^ example, and improved outcomes for women and children in communities with these groups are believed to be partly due to participants demanding better care, such as safe hygienic practices during childbirth. Community monitoring programmes can generate demand for quality, although few high-quality studies exploring this outcome exist (see Section 3).^300^ A programme^301,302^ in rural Uganda, for example, combined information sharing about quality care at local facilities with community participation and found reductions in neonatal deaths and improvements in measures of facility process quality 4 years after implementation. A study^303^ in Uttar Pradesh, India, showed that quality during prenatal visits was improved by sharing information about health and social service entitlements with pregnant women. A preliminary body of qualitative research^304^ also suggested that demand generation for quality might be especially well suited to improving user experience. Panel 14 includes examples of the use of advocacy to generate demand for high-quality care from the White Ribbon Alliance.

These interventions are based on sharing information with people and treating them as active agents in the health system. They are unlikely to work without system-level support that encourages patient-centredness, power-sharing, communication, and inclusion.^300^ Importantly, this supporting of people to be active agents should be done with careful attention to marginalised populations. The intersection of multiple sources of vulnerability is likely to make some groups less able and prepared to act on quality information than others. To prevent the exacerbation of existing disparities, particular attention must be paid to rural, less educated, and impoverished populations (see Section 3).

Interventions that might raise expectations and demand for quality often include social interaction through groups, committees, or meetings; this component is supported by social network science and evidence showing that people learn about quality from each other.^305,306^ This insight from social network science also suggests that demand generation interventions might take advantage of the increasing presence of interactive social media platforms in LMICs. Figure 14 gives an example of a people-facing dashboard that can be used to share information with populations. More country examples of improvement through the four universal actions can be found in appendix 2.

### Targeted opportunities

In conjunction with system-wide reform through the universal actions just described, countries will likely require additional context-specific interventions, which we call targeted opportunities. Beyond increasing health system inputs, this subsection reviews the most commonly used quality improvement interventions, but does not aim to present a comprehensive list. As mentioned in Section 2, the cost of interventions is not addressed in this report. We used several sources, including the Health Care Provider Performance Review (HCPPR)—a systematic review of health worker performance improvement strategies in LMICs (appendix 1).^255^ HCPPR was designed to develop evidence-based guidance on strategies to improve health worker performance and includes published and unpublished studies in any language from the 1960s to early 2016. Although the HCPPR is the most comprehensive and up-to-date review on the subject, it has limitations common to all reviews, such as implementation strength that varies across studies, and the unknown degree to which results from controlled study settings can be generalised to real-world programmes. The full review reported effect sizes for combinations of strategies, which are not discussed in detail here.

#### Macro level improvement: financing for quality

Health financing and provider payment can be used to leverage greater quality from the health system. Of the four core financing functions (revenue mobilisation, pooling, purchasing, and benefit design), purchasing—or the allocation of funds to providers—has the greatest direct influence on quality of care and we focus on it here.^252^

Strategic purchasing refers to funding providers on the basis of information about populations and providers to achieve performance goals. Examples include provider payment strategies and selective contracting of facilities on the basis of quality.^307^ Although most doctors and nurses are assumed to be motivated by altruism, they also seek a competitive wage. Input-based (eg, salary and capitation) and output-based (eg, fees-for-service, per case, or pay-for-performance) payments tend to exert opposite effects on providers’ intensity of effort, with input-based payments disincentivising and output-based payments promoting the number and intensity of services, leading to the duelling challenges of under-treatment and overtreatment described in Section 2. A mix of input and output financing might therefore be the best strategy to prevent undue attention to incentivised elements.

*Panel 14:* Lessons in generating community demand for quality care from the White Ribbon Alliance (WRA)

**Uganda**

In 2011, WRA Uganda mobilised local advocacy teams to bring attention to the poor quality of obstetric services in the country in three underserved districts. The teams, comprising district leaders, health officers, community members, and midwives, assessed the status of facilities and found that none of the districts met the minimum requirements for treating complications: they had insufficient lifesaving commodities, skilled health workers, and infrastructure. On the basis of similar findings, WRA launched the Act Now to Save Mothers campaign to educate citizens on their rights and responsibilities related to quality health care. Community members participated in district planning and budget hearings and town halls. In one town hall, more than 2000 community members signed and presented a petition to district representatives and parliament demanding improvement. Community members served as citizen journalists, reporting on progress and budget allocations. The campaign resulted in increased procurement of essential medicines and equipment, increases in salary for—and recruitment of—additional health workers, and the reconstruction of dilapidated facilities.

**Tanzania**

After a woman died in childbirth in 2013, in Rukwa region, Tanzania, because of no available blood supply, communities demonstrated in protest of the poor-quality care. WRA Tanzania brought together citizens and decision makers to ensure that these concerns were heard. They worked with religious leaders and village health teams to raise awareness among community members, and they supported district and regional policy makers to respond and act on citizen demands. The Parliamentarian Group for Safe Motherhood was mobilised to add their support to the citizens’ voices. In 2015, Rukwa leaders expanded emergency maternal health services from only 10% to 50% of health centres. On the basis of this success, WRA Tanzania expanded their efforts nationally, resulting in the government approving an historic 50% increase in funding for maternal, newborn, and child health to support expansion of facilities, blood banks, and the recruitment of health workers throughout the country.

**Lessons**

- Mobilise around existing political commitments for improvement
- Educate citizens about rights, responsibilities, and how to advocate
- Use data to create pressure for accountability
- Identify champions to amplify the voices of people
- Support decision makers to respond to citizen demand and collaborate with them to make change

Source: Kristy Kade, Betsy McCallon, Rose Mlay, Robina Biteyi.

Aligning financing and provision arrangements is crucial to the success of strategic purchasing. For example, facilities subject to selective contracting should be able to make the necessary purchasing and hiring decisions for improvement. In some countries, facilities might not have sufficient managing authority and legal changes will be required. Output-based financing is also data intensive and can be a substantial burden for providers. To align different payment methods and incentives, a strong data system to capture information on provider payments is crucial.^308^ Such systems produce information with multiple uses beyond strategic purchasing. For example, in high-income countries, insurance claims data offer information on services rendered, fees received, and diagnoses made that can be used by payers, insurers, and researchers.

One prominent form of strategic purchasing that has been widely implemented in LMICs is performance-based financing. Performance-based financing describes a set of approaches designed to improve health care by paying providers and facilities for the quantity and quality of care, though many programmes complement the financial incentives with direct improvement elements, such as training or supervision.^184,309–311^ Most performance-based financing programmes in LMICs incentivise primarily the quantity of services and, although they appear to increase utilisation of care and service volume, the effect of performance-based financing on quality is less clear.^184,310^ Several impact evaluations are forthcoming from the World Bank’s Health Results Innovation Trust Fund, a large funder of performance-based financing.^312,313^

Overall, evidence to date suggests that performance-based financing has insufficient potential as a standalone strategy for system-wide quality improvement. Performance-based financing might modestly improve quality compared with no intervention in some contexts, but does not always outperform unconditional financing; the effect appears to be driven not by the financing mechanism as much as the equipment and workforce interventions.^314–317^ Compared with alternative interventions, performance-based financing incurs unique costs for performance verification that can account for 10% to 15% of operating costs, including the cost of staff time.^318^ There can also be unintended consequences of performance-based financing programmes, including reports that providers have threatened patients to report positive outcomes, but these programmes can also improve unrewarded dimensions of quality, including patient satisfaction.^314,316,319^ Overall, performance-based financing does not appear to be highly cost-effective, especially vis-à-vis unconditional additional financing.^316,317^ The HCPPR showed that interventions that include financial incentives have a median increase on quality of 7·6 percentage points.

*Panel 15:* Case studies of learning at the district level*

**Midwifery Coordination Alliance Teams (MCAT) in Cambodia**

The MCAT programme is an area-based approach that started in select provinces in Cambodia, in 2009, and has since spread to all midwives, health centres and hospitals in all 98 districts of the country. The programme aims to strengthen collaboration between primary health care and hospital providers. All primary care midwives in a meso-level area meet doctors and midwives from the local hospital every 3 months for data review, updates, problem solving, and refresher trainings. The sessions are duplicated over 2 consecutive days to accommodate all health centre midwives without closing any services. Providers view and discuss data, such as maternal and perinatal deaths or near-misses, contraceptive uptake and mix, case-fatality of common conditions, and supervision results. The meetings include participatory learning sessions with simulations and discussions of current clinical procedures and guidelines. Supply chain issues, for example, surface and are solved with feedback and joint problem solving.

The teams also foster local innovations. For example, midwives in one province instituted a clinical hotline, which enabled health centre midwives to call a hospital midwife for advice on emergency referrals and follow-up. This idea has spread to other districts. Another MCAT team suggested and instituted an extension of the livebirth incentive to also include appropriate emergency referrals. Although a formal evaluation has not been done, the MCAT programme provides a team approach to gradually improving care of maternal and newborn complications in the district, and it is believed to be a factor in Cambodia’s large health gains for women and children.^A93,A94^

**Area-based planning and vertical integration in meso-America**

The Salud Mesoamerica Initiative (SMI) aims to reduce maternal and child health inequities through a results-based funding model to improve quality and effective coverage in seven Central American countries and in Chiapas state, Mexico. The programme features area-based plans within each health district to translate national plans to local teams. These plans include locally-tailored targets, activities, and timelines. Local implementers review their progress with national stakeholders every 3 months, fostering an experience-based learning environment.^A95^

SMI provides technical assistance to countries to create quality improvement strategies and standards through problem identification, prioritisation of areas for improvement, and development of improvement plans. Countries developed tools for data collection and analysis to support learning. Each country has adapted implementation to fit their priorities and systems. In Belize, the process started at the comprehensive emergency obstetric and newborn care level, then gradually added basic and ambulatory levels of care to allow teams to have a more holistic view of the health network. Teams collect data and review their own progress each month, and every quarter, a quality improvement officer reviews the performance to allocate a small incentive to teams through a Quality Innovation Fund. The quality improvement officer also offers supportive supervision, shares challenges and best practices, and helps teams to develop and test new ideas. Independent evaluation results showed that all indicators across levels of care have improved relative to baseline, with gains ranging from 30 to 85 percentage points.

Source: Som Hun and Jerker Liljestrand.

Source: Emma Margarita Iriarte and Jennifer Nelson. *Panel references can be found in appendix 1.

For financial incentives, including performance-based financing programmes, to be successful, economic theory and research suggest that rewards will have smaller effects than penalties because of the tendency to avoid losses, and incentives are most effective when closely linked to processes under the direct control of providers.^320,321^ Extrinsic incentives might crowd out intrinsic motivation, underlining the importance of aligning incentives with professional expectations and work norms.^320^ Finally, aligning provider incentives with specific goals of care coordination or effective treatment, as opposed to inputs, offers potential for quality improvement.^322^

#### Meso-level interventions: district-led learning

District administrations and networks of facilities can be harnessed into learning systems that accelerate improvements in health-care performance with the potential for scale. This level of the health system is well positioned to facilitate systematic group learning among facilities of similar types and across tiers of the health system. District-led, area-based learning and planning brings together providers and administrators responsible for a catchment area to solve clinical and system problems, harmonise approaches, maximise often scarce resources, and create better communication and referral between facilities (panel 15).^323^

Formal quality improvement collaboratives involve the use of teams from multiple health-care sites that work to improve performance on a specific topic by collecting and using data to test ideas with so-called plan-do-study-act cycles supported by coaching and learning sessions. Systematic reviews^324,325^ of quality improvement collaboratives in predominantly high-income countries showed modest improvements, particularly when addressing a clear gap between evidence and practice on straightforward aspects of care. Evidence from LMICs is more scarce, leading this Commission to undertake a subanalysis of quality improvement collaboratives based on the HCPPR systematic review.

Overall, the quality of evidence on quality improvement collaboratives from LMICs is low. Effect sizes for these collaboratives combined with clinical training were very large (mean range 52·4 to 111·7 percentage points) although how generalisable they are is uncertain, as three of the four reports targeted the same clinical outcome (postpartum haemorrhage), which was amenable to simple changes. The effectiveness of quality improvement collaboratives was more variable when implemented without training and when addressing other areas of care. Results on improving health worker practices ranged from modestly to highly effective (4·3 percentage points for continuous outcomes and 30·2 percentage points for percentage outcomes). For patient health outcomes, quality improvement collaboratives had no effect (1·4 percentage points for continuous outcomes and 0·3 percentage points for percentage outcomes).^255^ Quality improvement collaboratives are not static structures, and they have been implemented and adapted in several ways to achieve their stated aims. Some common adaptations include their use for the generation of new ideas and for empowerment of health-care workers. In addition to understanding the effect of district-led learning on clinical practice and patient outcomes, the effects of this approach on communication, health worker motivation, and team dynamics are currently being explored.^326^

#### Micro-level interventions: directly improving provider and facility performance

Strategies that target the micro-level are presented here as opportunities to complement and extend broader systems-level reforms. For example, the improved education of health professionals can be reinforced through facility-level refresher training. This Commission recommends that, where possible, micro-level interventions should not be implemented in isolation or instead of strategies that assess and improve the foundations of health systems.

We present in table 3 results of approaches to improve health worker performance, focusing on the six strategies tested by the largest number of studies and that included at least four low or moderate risk-of-bias studies.^255^ All six strategies target the health system at the micro-level. Moderate effect sizes were found for training (9·7 percentage points) and supervision (11·2 percentage points). The combination of training and supervision had larger improvement effects, at 17·8 percentage points. Providing only printed information or job aids to health workers and only implementing mHealth (a mobile wireless technology) strategies tended to be largely ineffective.

**Table 3:** Selected results of strategies to improve health worker performance from the Health Care Provider Performance Review^255^

	Training only	Training plus supervision^*^	Supervision^*^ only	Printed information or job aid for health workers only	Information communication technology (mHealth) only	Training plus supervision plus strengthening infrastructure^*^
Median effect size for percentage outcomes, percentage points (IQR)	9·7 (5·5–21·3)	17·8 (5·5–25·9)	11·2 (5·8–25·6)	1·5 (–4·5 to 6·1)	1·0 (–2·8 to 10·3)	9·4 (–0·1 to 40·5)
Study comparisons for percentage outcomes (comparisons with low or moderate risk of bias)	76 (32)	26 (11)	16 (8)	8 (5)	4 (4)	4 (1)
Median effect size for continuous outcomes, percentage points (IQR)	17·5 (0·1–23·7)	11·1 (7·3–60·4)	–3·0 (no IQR)	–3·4 (no IQR)	–38·9 (no IQR)	64·3 (31·9–88·7)
Study comparisons for continuous outcomes (comparisons with low or moderate risk of bias)	16 (8)	8 (3)	3 (1)	3 (1)	1 (1)	4 (4)
Countries in which studies were done for both outcomes types (number of WHO regions)	2 (6)	17 (5)	13 (5)	7 (3)	4 (1)	6 (3)
Three most common categories of study outcomes for both continuous and percentage measures	Treatment, counselling, assessment	Treatment, counselling, assessment	Treatment, counselling, universal precautions	Treatment, documentation, case management†	Counselling, case management,† treatment	Treatment, diagnosis, referral
Median baseline outcome value for percentage outcomes, % (IQR)	43·0 (19·0–70·0)	25·2 (9·2–52·5)	53·8 (36·0–63·5)	32·8 (24·8–58·6)	36·5 (5·4–60·6)	31·2 (7·7–55·6)
Median number of health facilities in intervention group for percentage outcomes (IQR)	6 (1–20)	7 (2–32)	7 (5–24)	8 (5–10)	38 (35–47)	11 (2–21)
Median duration of study follow-up‡ for percentage outcomes, in months (IQR)	4·0 (1·3–6·0)	4·5 (2·0–6·0)	5·0 (2·0–9·0)	1·9 (1·0–4·5)	7·3 (4·1–9·4)	1·6 (1·0–2·4)

Strategies for health facility-based health workers were tested by at least four studies with low or moderate risk of bias, from at least one outcome group (percentage outcomes or continuous outcomes). Percentage outcomes are expressed as a percentage (eg, percentage of patients treated correctly) and continuous outcomes are outcomes that could not be expressed as a percentage (eg, average number of medicines prescribed per patient); for details, see appendix 1.

* Supervision is either strengthened routine supervision visits (in terms of frequency or supervision quality) or other supervision-like strategies, such as audit with feedback; strengthening infrastructure is the provision of medicines or equipment, or otherwise improvement of conditions in health facilities.

† The case management group of outcomes reflect multiple steps of the case-management process (eg, percentage of patients correctly diagnosed and treated).

‡ Study follow-up time was defined as the time from when the strategy was initially implemented to the last eligible follow-up measure; for most study comparisons, the follow-up time was the same for all study outcomes; when follow-up time varied among outcomes for a given study comparison, the longest follow-up time was used in the analysis.

Most strategies that focused on improving the practices of lay or community health workers were tested by a single study and the quality of evidence was generally low. Again, training alone tended to have modest effect sizes (median of 7·3 percentage points). Strategies that included mHealth had a median effect size of 8·7 percentage points. Strategies that included training and supervision had a median effect size of 9·6 percentage points, and strategies that included training and community support approaches, such as patient education, had a median effect size of 22·7 percentage points.

Despite the scope and range of the studies, it is difficult to draw conclusions about how generalisable these strategies are. Studies tended to only include small numbers of health facilities in the intervention group (often less than ten) and short post-intervention follow-up times (median of 7 months or less). Effect sizes for strategies tested by multiple studies included in the HCPPR also varied considerably, which might be due to study biases, random variation, and considerable heterogeneity of study methods and context. For example, the effectiveness of complex, multifaceted interventions with at least four strategy components varied substantially (from nearly 0 to 61 percentage points), which suggests that complex strategies are sometimes, but not always, more effective than simpler ones, and clearly more work is needed in designing and testing these approaches. The variability of effect sizes for a given strategy also shows the difficulty in predicting how effective a strategy will be in a given context. Therefore, it is important for programmes to monitor the effectiveness of any strategy implemented in the field, not just in the context of research.

Additionally, the duration of strategy effectiveness is uncertain. Using HCPPR data, we modelled the effect of follow-up time on strategy effectiveness, using random-effects models (or fixed-effect models, if dealing with less than ten studies or outcomes) adjusted for baseline performance (appendix 1). We found that the effect of supervision appeared to increase over time, but we found no evidence of a time trend for group problem solving. Results for training were inconclusive. Our ability to examine time trends was limited by the small number of studies per strategy with repeated post-intervention measures.

### Section 5 conclusion

All reforms for quality will need to be country-led, starting with a vision for quality health care shared and actively supported by heads of state and their ministers. Many of these political leaders have already made commitments to UHC, but without improving quality, that promise is an empty one. Sequencing improvement efforts to first target populations who have the worst quality of care and health outcomes will also be important to realising high-quality UHC.^135,327^ Global partners are encouraged to support these efforts by aligning with each country’s priorities, not funding flotillas of small-scale interventions over short project-cycles, and instead selectively investing in fewer health system reforms over a longer time period. Reorienting research priorities to support country-led efforts for health system quality improvements is also sorely needed.

Every country will decide how to implement the systemic changes needed for high-quality care. This Commission recommends a careful assessment of the foundations of the national health system, consideration of the four universal actions we have presented, and a tailored strategy that addresses quality gaps and maximises existing assets. Finally, countries should not expect that every improvement initiative will succeed, but leaders should not be discouraged. The process of iteratively adapting and developing effective solutions for a given context takes time. The key is to get started, monitor progress, and learn from both successes and failures.

## Section 6: Recommendations

In this Section, we identify opportunities for national governments, civil society, global partners, and researchers to contribute to a global effort towards high-quality health systems.

### National governments

(1) Invest in health systems and make them more accountable to people. National governments need to invest in high-quality health systems for their own people, and they must also be accountable to people for their performance. This requires legislating for people’s right to quality health care, educating the population and health system stakeholders about these rights, enacting strong regulation and standard setting, sharing actionable information on health system performance, and creating mechanisms for remedy and redress. These actions should be complemented by social accountability mechanisms that promote the participation of the population in health system decisions. Countries will know they are on the way towards a high-quality, accountable health system when policy makers choose to receive their health care in their own public institutions.

(2) Look beyond the government health sector. Building high-quality health systems requires strong primary, secondary, and professional education, solid road and transport networks, and reliable communication infrastructure. Partnering with other sectors will be essential to create the conditions for health system reform. Involvement of private health-care providers and institutions can expand people’s choices and might spur the system to improve user focus; these private providers will need to be effectively regulated and incentivised to produce desired impacts.

(3) Embed quality of care in UHC. Quality should be at the core of UHC initiatives, alongside coverage and financial protection. For this, countries should begin by establishing a national quality guarantee for services provided through UHC that specifies the level of competence and user experience that people can expect in the health system. To ensure that poor people benefit from improved services, expansion should start with them. Progress on UHC should be measured through effective (quality-corrected) coverage.

(4) Measure better. Quality measurement should be parsimonious, timely, and transparent. Health systems should report their performance to the public annually using dashboards on health and wellbeing, user experience, system competence, and population confidence. Data should further be disaggregated across regions and vulnerable groups. Countries need to update their health system data toolkits, and they can begin by shedding uninformative indicators and instruments and improving data quality in existing systems. The high-quality health systems toolkit should include vital registries and real-time health system intelligence systems on supply chain and human resources, reliable routine information systems, and targeted studies, such as rapid facility surveys and updated population surveys. Investing in national institutions and expertise for measurement and translation of evidence to policy is crucial for making use of the data. Health and data literacy are also crucial for health-care users.

(5) Improve quality by starting with four universal actions. This Commission recommends that countries consider four universal actions to shift the trajectory toward high-quality health systems. Additional targeted opportunities in areas such as health financing, district-level learning, and others can complement these efforts. All strategies need careful monitoring and evaluation to measure their effect and allow local adaptations.

The first action is to govern for quality; this means creating a shared vision for a high-quality and learning health system with a national quality policy strategy and mechanisms for implementation and accountability. These should be developed in partnership with the private sector, civil society, and in collaboration with non-health sectors. A learning system needs accurate and timely data and a health system leadership committed to improvement. Improving data literacy for health workers and consumers will be needed to make use of the data.

The second action is quality-focused service delivery redesign; this requires reorganising health services to maximise health outcomes rather than solely geographic access to clinics. Primary care clinics should not tackle serious or rare health needs with elevated risk of mortality, such as deliveries, but should instead expand on their core competencies: integrated and continuous care for stable patients and community outreach and prevention. Governments and civil society need to work together to ensure that people can reach the care they need, when they need it, and that they receive respectful care; a range of strategies within and beyond the health sector are available.

The third action is transforming the workforce, starting with a move to competency-based clinical education that includes active learning, early clinical exposure, and problem-based learning. The curriculum should include ethics, respectful care, and core quality concepts. Classroom instruction needs to be buttressed with role-modelling and supervision in practice settings. The workforce should be supported with good working conditions, regular pay, and clinical mentorship and be provided with opportunities to learn and grow. Health workers and their professional associations must redouble efforts to maintain and enforce high standards of practice to earn and keep the public’s trust.

The fourth action is igniting demand for quality, which requires educating people about their health entitlements according to national resources and use targeted quality reporting, social networks, and mobile technologies to empower people to become active patients who seek and motivate health workers to provide good quality care.

### Civil society and non-governmental organisations

(1) Demand more from providers and health systems. People need to inform themselves on their rights and entitlements in the health system, including the right to competent care, respect, information, privacy, consent, and confidentiality. People enrolled in UHC programmes need to understand their benefit packages, care options, and communicate their needs and preferences to their providers. They should make use of redress options when care falls below the quality standard.

(2) Agitate for change and hold systems to account. Civil society should insist on transparent sharing of health system capacity and performance. They should press for greater social accountability through citizen report cards, community monitoring, social audits, participatory budgeting, citizen charters, and health committees. However, social accountability is not a replacement for government-led accountability; they are most successful in improving health system performance when combined.

### Global bilateral, multilateral, and foundation partners

(1) Invest in national institutions to produce evidence on health system quality. Many LMICs do not have effective institutions to do the functions in metrics, research, and evidence-based planning required for a learning health system. Global partners should support the international and national training of data scientists and help build institutional capability through mentoring and sharing of organisational best practices. Policy uptake of analytical findings from local institutions can in turn build confidence in and demand for locally generated evidence.

(2) Support development of health system quality measures. LMICs do not have measures that capture the elements of health system performance that matter to people and can inform improvement. Continued support of vital registries and health information systems to measure health and impacts is crucial. Agile facility surveys and real-time measures that capture quality of care and people’s voice and that can be linked to population health data are needed. Global repositories of validated comparable measures, instruments, and best practices in analysis can be a valuable resource.

*Panel 16:* High-quality health systems research agenda

**Measuring and analysing quality**

- Develop and validate quality measures suitable for resource-constrained settings for: health outcomes that can be attributed to health systems and patient-reported outcomes; competent care for mothers and newborn babies, cardiovascular diseases, chronic respiratory diseases, diabetes, cancer, mental health, and injuries; user experience, including respect, dignity, and autonomy; system and platform competence (eg, timeliness, safety, and integration), including quality of community outreach, primary care, and hospital care
- Understand the extent and causes of variations in quality: identify best performing countries, regions, and facilities and determine the factors contributing to their higher-quality care; explore causes of poor quality across different contexts
- Assess equity of quality care across dimensions of vulnerability, including setting of care, demographics, and disease type
- Analyse the effect of quality care on health, confidence, and economic outcomes, including patient-reported outcomes, demand for health care and bypassing, health system waste, and catastrophic and impoverishing expenditures

**Improving quality**

- Test the effect of innovations in the preservice education of health professionals on delivery of competent and respectful care
- Evaluate effects of quality-centred health service design on health, user experience, equity of care, and health system function
- Explore individual and combinations of interventions to generate community demand for quality, including dissemination of locally relevant information and innovations that use new technologies
- Refine the best design for district-level learning strategies (eg, quality improvement collaboratives and other approaches)
- Analyse the effects of legal, performance, and social mechanisms to promote accountability in low-income and middle-income countries
- Test management innovations and intrinsic and extrinsic approaches to motivate providers
- Measure the costs and cost-effectiveness of improvement approaches and their sustainability

**Methods and tools**

- Develop an agile facility survey for rapid measurement of health system quality that focus on measures that matter: competent care and systems and user experience
- Update population surveys to measure a broader range of health conditions
- Explore new technologies to improve accuracy and reduce the burden of process and outcome measurements (eg, wearable trackers, big data analytics)
- Expand and validate methods for measuring effective coverage
- Develop new methods to test system competence over time, such as tracer patients
- Incorporate implementation science in assessments of health system improvement strategies to understand what works, why, and in what contexts
- Expand the use of qualitative methods and approaches from social sciences, such as political and management science, in describing and diagnosing quality failures and successes
- Expand sample sizes and extend length of time in studies of all improvement strategies, to characterise the generalisability and sustainability of these approaches
- Include patient experience and patient-reported outcomes in improvement research

(3) Include quality in tracking progress of global initiatives. Progress on UHC and the SDGs should include both coverage and quality, and effective (quality-corrected) coverage brings these concepts together. Several appropriate indicators are available while others require validation; more work is needed to build these indicators into global measure sets.

(4) Channel donor funding to universal actions for improvement. Large-scale improvements such as health education reform or service delivery redesign are costly, and some low-income countries will require external financing to undertake it. More generally, funders should align their support with country strategies that promote the evolution toward a higher-quality health system and avoid funding a multitude of small scale or vertical initiatives, which contributes to policy and programming confusion and reduces resources for large-scale action.

(5) Fund research on system-wide improvement strategies. Rigorous evaluation of improvement reforms is needed to gauge the effect of investing in reforms on health. Research can inform future national investment, help develop local capacity, and benefit other countries with similar contexts. Creating platforms for regional learning through networks and meetings can promote dissemination of success and avoid the replication of failed ideas.

### Researchers

(1) Measure quality and evaluate quality improvement. Research is not a luxury: mismeasurement, reliance in assumptions rather than evidence, and the replication of failed ideas costs lives, squanders trust, and wastes resources. Data on health-care quality in LMICs do not reflect the current disease burden; for example, we know little about quality of care for diabetes and cardiovascular diseases and almost nothing about respiratory disease, cancer, mental health, injuries, and surgery. Adolescents and older adults are less visible in the available data than other age groups. Available data give a better picture of episodic, routine care than of longitudinal services or treatment of acute events, such as maternal or newborn complications or medical and surgical emergencies. Filling in these gaps will require better routine data collection and new research. In assessing quality improvement, we found that the evidence base for many popular approaches is surprisingly weak. Rigorous assessments of all improvement strategies, ideally with implementation science methods, will be essential to justify their scale-up. This Commission’s research priorities are shown in panel 16.

### Conclusion

Although health systems will look different in different settings, all people should be able to count on receiving high-quality care that will improve their health and earn their trust. It is time to rethink our past approaches: to ask more from and invest more in this crucial determinant of health.

## References

[1] Institute for Health Metrics and Evaluation Financing global health 2012: the end of the golden age? Seattle, WA: IHME, 2012.

[2] Dieleman J, Campbell M, Chapin A, et al, and the Global Burden of Disease Health Financing Collaborator Network Evolution and patterns of global health financing 1995–2014: development assistance for health, and government, prepaid private, and out-of-pocket health spending in 184 countries. Lancet 2017; 389: 1981–2004. 28433256 10.1016/S0140-6736(17)30874-7 PMC5440770

[3] Victora CG, Requejo JH, Barros AJ, et al Countdown to 2015: a decade of tracking progress for maternal, newborn, and child survival. Lancet 2016; 387: 2049–59. 26477328 10.1016/S0140-6736(15)00519-X PMC7613171

[4] Xu K, Soucat A, Kutzin J, et al New perspectives on global health spending for universal health coverage. Geneva: World Health Organization, 2017.

[5] Murray CJ, Ortblad KF, Guinovart C, et al Global, regional, and national incidence and mortality for HIV, tuberculosis, and malaria during 1990–2013: a systematic analysis for the Global Burden of Disease Study 2013. Lancet 2014; 384: 1005–70. 25059949 10.1016/S0140-6736(14)60844-8 PMC4202387

[6] Lozano R, Naghavi M, Foreman K, et al Global and regional mortality from 235 causes of death for 20 age groups in 1990 and 2010: a systematic analysis for the Global Burden of Disease Study 2010. Lancet 2012; 380: 2095–128. 23245604 10.1016/S0140-6736(12)61728-0 PMC10790329

[7] Tessema GA, Laurence CO, Melaku YA, et al Trends and causes of maternal mortality in Ethiopia during 1990–2013: findings from the Global Burden of Diseases study 2013. BMC Public Health 2017; 17: 160. 28152987 10.1186/s12889-017-4071-8 PMC5290608

[8] Ng M, Misra A, Diwan V, Agnani M, Levin-Rector A, De Costa A. An assessment of the impact of the JSY cash transfer program on maternal mortality reduction in Madhya Pradesh, India. Glob Health Action 2014; 7: 24939. 25476929 10.3402/gha.v7.24939 PMC4256523

[9] Bohren MA, Vogel JP, Hunter EC, et al The mistreatment of women during childbirth in health facilities globally: a mixed-methods systematic review. PLoS Med 2015; 12: e1001847. 26126110 10.1371/journal.pmed.1001847 PMC4488322

[10] Valentine N, Darby C, Bonsel GJ. Which aspects of non-clinical quality of care are most important? Results from WHO’s general population surveys of “health systems responsiveness” in 41 countries. Soc Sci Med 2008; 66: 1939–50. 18313822 10.1016/j.socscimed.2007.12.002

[11] Brownlee S, Chalkidou K, Doust J, et al Evidence for overuse of medical services around the world. Lancet 2017; 390: 156–68. 28077234 10.1016/S0140-6736(16)32585-5 PMC5708862

[12] Marmot M, and the Commission on Social Determinants of Health Achieving health equity: from root causes to fair outcomes. Lancet 2007; 370: 1153–63. 17905168 10.1016/S0140-6736(07)61385-3

[13] UN Committee on Economic, Social, and Cultural Rights General Comment No. 14: the right to the highest attainable standard of health (art. 12 of the Covenant); E/C.12/2000/4. 2000 http://www.refworld.org/docid/4538838d0.html (accessed April 25, 2018).

[14] Jamison DT, Alwan A, Mock CN, et al Universal health coverage and intersectoral action for health: key messages from Disease Control Priorities, 3rd edn. Lancet 2017; 391: 1108–20. 29179954 10.1016/S0140-6736(17)32906-9 PMC5996988

[15] Nolte E, McKee CM. Measuring the health of nations: updating an earlier analysis. Health Aff (Millwood) 2008; 27: 58–71. 18180480 10.1377/hlthaff.27.1.58

[16] WHO Everybody’s business–strengthening health systems to improve health outcomes: WHO’s framework for action. Geneva: World Health Organization, 2007.

[17] WHO, World Bank Tracking universal health coverage: first global monitoring report: World Health Organization, World Bank, 2015. 2015 http://www.who.int/healthinfo/universal_health_coverage/report/2015/en/ (accessed Aug 14, 2018).

[18] Kruk ME, Pate M, Mullan Z. Introducing The Lancet Global Health Commission on high-quality health systems in the SDG era. Lancet Glob Health 2017; 5: e480–81. 28302563 10.1016/S2214-109X(17)30101-8

[19] Kieny M-P, Evans TG, Scarpetta S, et al Delivering quality health services: a global imperative for universal health coverage. Washington, DC: World Bank Group, 2018.

[20] Kruk ME, Nigenda G, Knaul FM. Redesigning primary care to tackle the global epidemic of noncommunicable disease. Am J Public Health 2015; 105: 431–37. 25602898 10.2105/AJPH.2014.302392 PMC4330840

[21] PHCPI Primary Health Care Performance Initiative. 2017 https://phcperformanceinitiative.org (accessed Dec 22, 2017).

[22] Donabedian A. Evaluating the quality of medical care. Milbank Mem Fund Q 1966; 44: 166–206. 5338568

[23] Institute of Medicine Committee on Quality of Health Care in America Crossing the quality chasm: a new health system for the 21st century. Washington, DC: National Academies Press, 2001.

[24] Institute of Medicine Committee on Quality of Health Care in America To err is human: building a safer health system. Washington, DC: National Academies Press, 2000. 25077248

[25] Porter ME. What is value in health care? N Engl J Med 2010; 363: 2477–81. 21142528 10.1056/NEJMp1011024

[26] WHO WHO global strategy on people-centred and integrated health services. 2015 http://www.who.int/servicedeliverysafety/areas/people-centred-care/global-strategy/en/ (accessed Aug 14, 2018).

[27] Tunçalp Ö, Were WM, MacLennan C, et al Quality of care for pregnant women and newborns-the WHO vision. BJOG 2015; 122: 1045–49. 25929823 10.1111/1471-0528.13451 PMC5029576

[28] Bruce J. Fundamental elements of the quality of care: a simple framework. Stud Fam Plann 1990; 21: 61–91. 2191476

[29] Rockers PC, Kruk ME, Laugesen MJ. Perceptions of the health system and public trust in government in low- and middle-income countries: evidence from the World Health Surveys. J Health Polit Policy Law 2012; 37: 405–37. 22323234 10.1215/03616878-1573076

[30] Backman G, Hunt P, Khosla R, et al Health systems and the right to health: an assessment of 194 countries. Lancet 2008; 372: 2047–85. 19097280 10.1016/S0140-6736(08)61781-X

[31] Kruk ME, Myers M, Varpilah ST, Dahn BT. What is a resilient health system? Lessons from Ebola. Lancet 2015; 385: 1910–12. 25987159 10.1016/S0140-6736(15)60755-3

[32] Gilson L, Barasa E, Nxumalo N, et al Everyday resilience in district health systems: emerging insights from the front lines in Kenya and South Africa. BMJ Glob Health 2017; 2: e000224. 10.1136/bmjgh-2016-000224 PMC5656138 29081995

[33] Roberts M, Hsiao W, Berman P, Reich M. Getting health reform right: a guide to improving performance and equity. Oxford: Oxford University Press, 2003.

[34] Taylor MJ, McNicholas C, Nicolay C, Darzi A, Bell D, Reed JE. Systematic review of the application of the plan-do-study-act method to improve quality in healthcare. BMJ Qual Saf 2014; 23: 290–98. 10.1136/bmjqs-2013-001862 PMC3963536 24025320

[35] Leslie HH, Sun Z, Kruk ME. Association between infrastructure and observed quality of care in 4 healthcare services: a cross-sectional study of 4,300 facilities in 8 countries. PLoS Med 2017; 14: e1002464. 29232377 10.1371/journal.pmed.1002464 PMC5726617

[36] Knaul FM, Farmer PE, Krakauer EL, et al Alleviating the access abyss in palliative care and pain relief—an imperative of universal health coverage: the *Lancet* Commission report. Lancet 2018; 391: 1391–454. 29032993 10.1016/S0140-6736(17)32513-8

[37] Ozawa S, Sripad P. How do you measure trust in the health system? A systematic review of the literature. Soc Sci Med 2013; 91: 10–14. 23849233 10.1016/j.socscimed.2013.05.005

[38] Kruk ME, Chukwuma A, Mbaruku G, Leslie HH. Variation in quality of primary-care services in Kenya, Malawi, Namibia, Rwanda, Senegal, Uganda and the United Republic of Tanzania. Bull World Health Organ 2017; 95: 408–18. 28603307 10.2471/BLT.16.175869 PMC5463807

[39] Semrau KEA, Hirschhorn LR, Marx Delaney M, et al, and the BetterBirth Trial Group Outcomes of a coaching-based WHO safe childbirth checklist program in India. N Engl J Med 2017; 377: 2313–24. 29236628 10.1056/NEJMoa1701075 PMC5672590

[40] Carvajal-Vélez L, Amouzou A, Perin J, et al Diarrhea management in children under five in sub-Saharan Africa: does the source of care matter? A Countdown analysis. BMC Public Health 2016; 16: 830. 27538438 10.1186/s12889-016-3475-1 PMC4991040

[41] Sylvia S, Xue H, Zhou C, et al Tuberculosis detection and the challenges of integrated care in rural China: a cross-sectional standardized patient study. PLoS Med 2017; 14: e1002405. 29040263 10.1371/journal.pmed.1002405 PMC5644979

[42] Daniels B, Dolinger A, Bedoya G, et al Use of standardised patients to assess quality of healthcare in Nairobi, Kenya: a pilot, cross-sectional study with international comparisons. BMJ Glob Health 2017; 2: e000333. 10.1136/bmjgh-2017-000333 PMC5717935 29225937

[43] WHO WHO Safe Childbirth Checklist. 2015 http://www.who.int/patientsafety/implementation/checklists/childbirth/en/ (accessed March 24, 2017).

[44] WHO WHO recommendations on antenatal care for a positive pregnancy experience. 2016 http://www.who.int/reproductivehealth/publications/maternal_perinatal_health/anc-positive-pregnancy-experience/en/ (accessed March 24, 2017). 28079998

[45] Arsenault C, Jordan K, Lee D, et al Equity in antenatal care quality: an analysis of 91 national household surveys. Lancet Glob Health (in press). 10.1016/S2214-109X(18)30389-9 PMC6187112 30322649

[46] Adesina A, Chumba D, Nelson AM, et al Improvement of pathology in sub-Saharan Africa. Lancet Oncol 2013; 14: e152–57. 23561746 10.1016/S1470-2045(12)70598-3

[47] Atun R, Davies JI, Gale EAM, et al Diabetes in sub-Saharan Africa: from clinical care to health policy. Lancet Diabetes Endocrinol 2017; 5: 622–67. 28688818 10.1016/S2213-8587(17)30181-X

[48] Leslie HH, Spiegelman D, Zhou X, Kruk ME. Service readiness of health facilities in Bangladesh, Haiti, Kenya, Malawi, Namibia, Nepal, Rwanda, Senegal, Uganda and the United Republic of Tanzania. Bull World Health Organ 2017; 95: 738–48. 29147054 10.2471/BLT.17.191916 PMC5677617

[49] Mendelson M, Matsoso MP. The World Health Organization global action plan for antimicrobial resistance. S Afr Med J 2015; 105: 325. 26242647 10.7196/samj.9644

[50] Peabody JW, DeMaria L, Smith O, Hoth A, Dragoti E, Luck J. Large-scale evaluation of quality of care in 6 countries of eastern Europe and central Asia using clinical performance and value vignettes. Glob Health Sci Pract 2017; 5: 412–29. 28963174 10.9745/GHSP-D-17-00044 PMC5620338

[51] Uwemedimo OT, Lewis TP, Essien EA, et al Distribution and determinants of pneumonia diagnosis using Integrated Management of Childhood Illness guidelines: a nationally representative study in Malawi. BMJ Glob Health 2018; 3: e000506. 10.1136/bmjgh-2017-000506 PMC5898357 29662688

[52] Das J, Holla A, Das V, Mohanan M, Tabak D, Chan B. In urban and rural India, a standardized patient study showed low levels of provider training and huge quality gaps. Health Aff (Millwood) 2012; 31: 2774–84. 23213162 10.1377/hlthaff.2011.1356 PMC3730274

[53] Mukadi P, Lejon V, Barbé B, et al Performance of microscopy for the diagnosis of malaria and human African trypanosomiasis by diagnostic laboratories in the Democratic Republic of the Congo: results of a nation-wide external quality assessment. PLoS One 2016; 11: e0146450. 26788725 10.1371/journal.pone.0146450 PMC4720473

[54] Goss PE, Lee BL, Badovinac-Crnjevic T, et al Planning cancer control in Latin America and the Caribbean. Lancet Oncol 2013; 14: 391–436. 23628188 10.1016/S1470-2045(13)70048-2

[55] Cazabon D, Suresh A, Oghor C, et al Implementation of Xpert MTB/RIF in 22 high tuberculosis burden countries: are we making progress? Eur Respir J 2017; 50: 1700918. 28860268 10.1183/13993003.00918-2017

[56] Glasziou P, Straus S, Brownlee S, et al Evidence for underuse of effective medical services around the world. Lancet 2017; 390: 169–77. 28077232 10.1016/S0140-6736(16)30946-1

[57] Johansson EW, Nsona H, Carvajal-Aguirre L, Amouzou A, Hildenwall H. Determinants of Integrated Management of Childhood Illness (IMCI) non-severe pneumonia classification and care in Malawi health facilities: analysis of a national facility census. J Glob Health 2017; 7: 020408. 29163934 10.7189/jogh.07.020408 PMC5680530

[58] Levi J, Raymond A, Pozniak A, Vernazza P, Kohler P, Hill A. Can the UNAIDS 90-90-90 target be achieved? A systematic analysis of national HIV treatment cascades. BMJ Glob Health 2016; 1: e000010. 10.1136/bmjgh-2015-000010 PMC5321333 28588933

[59] Subbaraman R, Nathavitharana RR, Satyanarayana S, et al The tuberculosis cascade of care in India’s public sector: a systematic review and meta-analysis. PLoS Med 2016; 13: e1002149. 27780217 10.1371/journal.pmed.1002149 PMC5079571

[60] Naidoo P, Theron G, Rangaka MX, et al The South African tuberculosis care cascade: estimated losses and methodological challenges. J Infect Dis 2017; 216 (suppl 7): S702–13. 29117342 10.1093/infdis/jix335 PMC5853316

[61] Manne-Goehler J, Atun R, Stokes A, et al Diabetes diagnosis and care in sub-Saharan Africa: pooled analysis of individual data from 12 countries. Lancet Diabetes Endocrinol 2016; 4: 903–12. 27727123 10.1016/S2213-8587(16)30181-4

[62] Thornicroft G, Chatterji S, Evans-Lacko S, et al Undertreatment of people with major depressive disorder in 21 countries. Br J Psychiatry 2017; 210: 119–24. 27908899 10.1192/bjp.bp.116.188078 PMC5288082

[63] Alshamsan R, Lee JT, Rana S, Areabi H, Millett C. Comparative health system performance in six middle-income countries: cross-sectional analysis using World Health Organization study of global ageing and health. J R Soc Med 2017; 110: 365–75. 28895493 10.1177/0141076817724599 PMC5987910

[64] Li J, Li X, Wang Q, et al, and the China PEACE Collaborative Group ST-segment elevation myocardial infarction in China from 2001 to 2011 (the China PEACE-Retrospective Acute Myocardial Infarction Study): a retrospective analysis of hospital data. Lancet 2015; 385: 441–51. 24969506 10.1016/S0140-6736(14)60921-1 PMC4415374

[65] Satyanarayana S, Kwan A, Daniels B, et al Use of standardised patients to assess antibiotic dispensing for tuberculosis by pharmacies in urban India: a cross-sectional study. Lancet Infect Dis 2016; 16: 1261–68. 27568359 10.1016/S1473-3099(16)30215-8 PMC5067371

[66] Li Y, Xu J, Wang F, et al Overprescribing in China, driven by financial incentives, results in very high use of antibiotics, injections, and corticosteroids. Health Aff (Millwood) 2012; 31: 1075–82. 22566449 10.1377/hlthaff.2010.0965

[67] Molina G, Weiser TG, Lipsitz SR, et al Relationship between cesarean delivery rate and maternal and neonatal mortality. JAMA 2015; 314: 2263–70. 26624825 10.1001/jama.2015.15553

[68] Reardon S. Antibiotic resistance sweeping developing world. Nature 2014; 509: 141–42. 24805322 10.1038/509141a

[69] Versporten A, Zarb P, Caniaux I, et al, and the Global-PPS network Antimicrobial consumption and resistance in adult hospital inpatients in 53 countries: results of an internet-based global point prevalence survey. Lancet Glob Health 2018; 6: e619–29. 29681513 10.1016/S2214-109X(18)30186-4

[70] Guanais F, Regalía F, Pérez-Cuevas R, Anaya M. Desde el paciente. Experiencias con la atención primaria de salud en América Latina y el Caribe. Washington, DC: Interamerican Development Bank, 2018.

[71] The Research Priority Setting Working Group of the World Alliance for Patient Safety Summary of the evidence on patient safety: implications for research. 2007 http://apps.who.int/iris/bitstream/10665/43874/1/9789241596541_eng.pdf (accessed Aug 14, 2018).

[72] Jha AK, Larizgoitia I, Audera-Lopez C, Prasopa-Plaizier N, Waters H, Bates DW. The global burden of unsafe medical care: analytic modelling of observational studies. BMJ Qual Saf 2013; 22: 809–15. 10.1136/bmjqs-2012-001748 24048616

[73] WHO Global guidelines for the prevention of surgical site infection. 2016 http://apps.who.int/iris/bitstream/10665/250680/1/9789241549882-eng.pdf?ua=1 (accessed Aug 14, 2018). 30689333

[74] WHO. UNICEF Water, sanitation and hygiene in health care facilities: status in low and middle income countries and way forward. 2015 http://apps.who.int/iris/bitstream/10665/154588/1/9789241508476_eng.pdf (accessed February 13, 2018).

[75] Bedoya G, Dolinger A, Rogo K, et al Observations of infection prevention and control practices in primary health care, Kenya. Bull World Health Organ 2017; 95: 503–16. 28670015 10.2471/BLT.16.179499 PMC5487970

[76] Macinko J, Guanais FC, Mullachery P, Jimenez G. Gaps in primary care and health system performance in six Latin American and Caribbean countries. Health Aff (Millwood) 2016; 35: 1513–21. 27503978 10.1377/hlthaff.2015.1366

[77] Pan AHO. Cervical Cancer Prevention and Control Programs: a rapid assessment in 12 countries of Latin America. 2010 http://www.paho.org/hq/index.php?option=com_docman&task=doc_view&gid=16119&Itemid=270&lang=en (accessed Aug 14, 2018).

[78] WHO Consolidated guidelines on HIV Testing Services. 2015 http://who.int/hiv/pub/guidelines/hiv-testing-services/en/ (accessed Aug 14, 2018).

[79] UNAIDS AIDSinfo. 2017 http://aidsinfo.unaids.org/ (accessed Aug 14, 2018).

[80] Wells JE, Browne MO, Aguilar-Gaxiola S, et al Drop out from out-patient mental healthcare in the World Health Organization’s World Mental Health Survey initiative. Br J Psychiatry 2013; 202: 42–49. 23174514 10.1192/bjp.bp.112.113134

[81] MacPherson P, Houben RM, Glynn JR, Corbett EL, Kranzer K. Pre-treatment loss to follow-up in tuberculosis patients in low- and lower-middle-income countries and high-burden countries: a systematic review and meta-analysis. Bull World Health Organ 2014; 92: 126–38. 24623906 10.2471/BLT.13.124800 PMC3949536

[82] WHO Global Tuberculosis Report 2017. 2017 http://www.who.int/tb/publications/global_report/en/ (accessed Aug 14, 2018).

[83] Matityahu A, Elliott I, Marmor M, Caldwell A, Coughlin R, Gosselin RA. Time intervals in the treatment of fractured femurs as indicators of the quality of trauma systems. Bull World Health Organ 2014; 92: 40–50. 24391299 10.2471/BLT.13.120436 PMC3865547

[84] Kruk ME, Kujawski S, Moyer CA, et al Next generation maternal health: external shocks and health-system innovations. Lancet 2016; 388: 2296–306. 27642020 10.1016/S0140-6736(16)31395-2 PMC5167371

[85] Neogi SB, Sharma J, Negandhi P, Chauhan M, Reddy S, Sethy G. Risk factors for stillbirths: how much can a responsive health system prevent? BMC Pregnancy Childbirth 2018; 18: 33. 29347930 10.1186/s12884-018-1660-1 PMC5774063

[86] Sreeramareddy CT, Panduru KV, Menten J, Van den Ende J. Time delays in diagnosis of pulmonary tuberculosis: a systematic review of literature. BMC Infect Dis 2009; 9: 91. 19519917 10.1186/1471-2334-9-91 PMC2702369

[87] E C, Dahrouge S, Samant R, Mirzaei A, Price J. Radical radiotherapy for cervix cancer: the effect of waiting time on outcome. Int J Radiat Oncol Biol Phys 2005; 61: 1071–77. 15752886 10.1016/j.ijrobp.2004.09.030

[88] Brinton L, Figueroa J, Adjei E, et al, and the Ghana Breast Health Study team Factors contributing to delays in diagnosis of breast cancers in Ghana, West Africa. Breast Cancer Res Treat 2017; 162: 105–14. 28025716 10.1007/s10549-016-4088-1 PMC5290196

[89] Instituto Mexicano del Seguro Social Indicadores médicos 2016: Procesos de Salud—Enfermedad en Población Derechohabiente. 2017 http://intranet/datos/infosalud/Paginas/Indicadores_Medicos.aspx (accessed Aug 14, 2018).

[90] Recondo G, Cosacow C, Cutuli HJ, et al Access to oncological care in patients with breast and lung cancer treated at public and private hospitals in Buenos Aires, Argentina. J Clin Oncol 2018; 36: e18640.

[91] Larson E, Leslie HH, Kruk ME. The determinants and outcomes of good provider communication: a cross-sectional study in seven African countries. BMJ Open 2017; 7: e014888. 10.1136/bmjopen-2016-014888 PMC5734554 28674138

[92] Doyle C, Lennox L, Bell D. A systematic review of evidence on the links between patient experience and clinical safety and effectiveness. BMJ Open 2013; 3: e001570. 10.1136/bmjopen-2012-001570 PMC3549241 23293244

[93] Irving G, Neves AL, Dambha-Miller H, et al International variations in primary care physician consultation time: a systematic review of 67 countries. BMJ Open 2017; 7: e017902. 10.1136/bmjopen-2017-017902 PMC5695512 29118053

[94] Kruk ME, Gage AD, Joseph N, Danei G, Garcia-Saiso S, Salomon JA. Mortality due to low-quality health systems in the universal health coverage era: a systematic analysis of amenable deaths in 137 countries. Lancet (in press). 10.1016/S0140-6736(18)31668-4 PMC6238021 30195398

[95] Alkire BC, Peters AW, Shrime MG, Meara JG. The economic consequences of mortality amenable to high-quality health care in low- and middle-income countries. Health Aff (Millwood) 2018; 37: 988–96. 29863936 10.1377/hlthaff.2017.1233

[96] Ronsmans C, Graham WJ, and the *Lancet* Maternal Survival Series steering group Maternal mortality: who, when, where, and why. Lancet 2006; 368: 1189–200. 17011946 10.1016/S0140-6736(06)69380-X

[97] Lawn JE, Blencowe H, Waiswa P, et al, the *Lancet* Ending Preventable Stillbirths Series study group, and the *Lancet* Stillbirth Epidemiology investigator group Stillbirths: rates, risk factors, and acceleration towards 2030. Lancet 2016; 387: 587–603. 26794078 10.1016/S0140-6736(15)00837-5

[98] Bhutta ZA, Das JK, Bahl R, et al, the *Lancet* Newborn Interventions Review Group, and the *Lancet* Every Newborn Study Group Can available interventions end preventable deaths in mothers, newborn babies, and stillbirths, and at what cost? Lancet 2014; 384: 347–70. 24853604 10.1016/S0140-6736(14)60792-3

[99] Allemani C, Matsuda T, Di Carlo V, et al, and the CONCORD Working Group Global surveillance of trends in cancer survival 2000–14 (CONCORD-3): analysis of individual records for 37513025 patients diagnosed with one of 18 cancers from 322 population-based registries in 71 countries. Lancet 2018; 391: 1023–75. 29395269 10.1016/S0140-6736(17)33326-3 PMC5879496

[100] Souza JP, Gülmezoglu AM, Vogel J, et al Moving beyond essential interventions for reduction of maternal mortality (the WHO Multicountry Survey on Maternal and Newborn Health): a cross-sectional study. Lancet 2013; 381: 1747–55. 23683641 10.1016/S0140-6736(13)60686-8

[101] Biccard BM, Madiba TE, Kluyts H-L, et al, and the African Surgical Outcomes Study (ASOS) investigators Perioperative patient outcomes in the African Surgical Outcomes Study: a 7-day prospective observational cohort study. Lancet 2018; 391: 1589–98. 29306587 10.1016/S0140-6736(18)30001-1

[102] Meara JG, Leather AJ, Hagander L, et al Global Surgery 2030: evidence and solutions for achieving health, welfare, and economic development. Lancet 2015; 386: 569–624. 25924834 10.1016/S0140-6736(15)60160-X

[103] Uribe-Leitz T, Jaramillo J, Maurer L, et al Variability in mortality following caesarean delivery, appendectomy, and groin hernia repair in low-income and middle-income countries: a systematic review and analysis of published data. Lancet Glob Health 2016; 4: e165–74. 26916818 10.1016/S2214-109X(15)00320-4

[104] Ng-Kamstra JS, Arya S, Greenberg SLM, et al Perioperative mortality rates in low-income and middle-income countries: a systematic review and meta-analysis. BMJ Glob Health 2018; 3: e000810. 10.1136/bmjgh-2018-000810 PMC6035511 29989045

[105] Obermeyer Z, Abujaber S, Makar M, et al, and the Acute Care Development Consortium Emergency care in 59 low- and middle-income countries: a systematic review. Bull World Health Organ 2015; 93: 577–586G. 26478615 10.2471/BLT.14.148338 PMC4581659

[106] Lu J, Lu Y, Wang X, et al Prevalence, awareness, treatment, and control of hypertension in China: data from 1·7 million adults in a population-based screening study (China PEACE Million Persons Project). Lancet 2017; 390: 2549–58. 29102084 10.1016/S0140-6736(17)32478-9

[107] Xu Y, Wang L, He J, et al Prevalence and control of diabetes in Chinese adults. JAMA 2013; 310: 948–59. 24002281 10.1001/jama.2013.168118

[108] Cisneros-González N, Ascencio-Montiel IJ, Libreros-Bango VN, et al Índice de amputaciones de extremidades inferiores en pacientes con diabetes. Rev Med Inst Mex Seguro Soc 2016; 54: 472–79. 27197105

[109] Stop TB. Partnership U. 90 (90) 90 The Tuberculosis Report for Heads of State and Governments. Geneva, Switzerland, 2017 http://www.stoptb.org/assets/documents/resources/publications/acsm/909090_PDF_LR.pdf (accessed Aug 14, 2018).

[110] Maheu-Giroux M, Filippi V, Samadoulougou S, et al Prevalence of symptoms of vaginal fistula in 19 sub-Saharan Africa countries: a meta-analysis of national household survey data. Lancet Glob Health 2015; 3: e271–78. 25889469 10.1016/S2214-109X(14)70348-1

[111] Maheu-Giroux M, Filippi V, Maulet N, et al Risk factors for vaginal fistula symptoms in Sub-Saharan Africa: a pooled analysis of national household survey data. BMC Pregnancy Childbirth 2016; 16: 82. 27098261 10.1186/s12884-016-0871-6 PMC4839076

[112] Guanais F, Regalia F, Perez-Cuevas R, Anaya M. Desde el paciente. Experiencias de la atención primaria de salud en América Latina y el Caribe. [From the patient. Experiences of primary health care in Latin America and the Caribbean.]. Washington, DC Inter-American Development Bank 2018.

[113] Penm J, MacKinnon NJ, Strakowski SM, Ying J, Doty MM. Minding the gap: factors associated with primary care coordination of adults in 11 countries. Ann Fam Med 2017; 15: 113–19. 28289109 10.1370/afm.2028 PMC5348227

[114] Deaton AS, Tortora R. People in sub-Saharan Africa rate their health and health care among the lowest in the world. Health Aff (Millwood) 2015; 34: 519–27. 25715657 10.1377/hlthaff.2014.0798 PMC5674528

[115] McCarthy EA, Subramaniam HL, Prust ML, et al Quality improvement intervention to increase adherence to ART prescription policy at HIV treatment clinics in Lusaka, Zambia: A cluster randomized trial. PLoS One 2017; 12: e0175534. 28419106 10.1371/journal.pone.0175534 PMC5395211

[116] Mekoth N, Dalvi V. Does quality of healthcare service determine patient adherence? Evidence from the primary healthcare sector in India. Hosp Top 2015; 93: 60–68. 26652042 10.1080/00185868.2015.1108141

[117] Gage AD, Leslie HH, Bitton A, et al Does quality influence utilization of primary health care? Evidence from Haiti. Global Health 2018; 14: 59. 29925416 10.1186/s12992-018-0379-0 PMC6011404

[118] Kruk ME, Hermosilla S, Larson E, Mbaruku GM. Bypassing primary care clinics for childbirth: a cross-sectional study in the Pwani region, United Republic of Tanzania. Bull World Health Organ 2014; 92: 246–53. 24700992 10.2471/BLT.13.126417 PMC3967574

[119] Leonard KL. Active patients in rural African health care: implications for research and policy. Health Policy Plan 2014; 29: 85–95. 23307907 10.1093/heapol/czs137

[120] Abrahim O, Linnander E, Mohammed H, Fetene N, Bradley E. A patient-centered understanding of the referral system in ethiopian primary health care units. PLoS One 2015; 10: e0139024. 26436759 10.1371/journal.pone.0139024 PMC4593586

[121] Li X, Lu J, Hu S, et al The primary health-care system in China. Lancet 2017; 390: 2584–94. 29231837 10.1016/S0140-6736(17)33109-4

[122] Fe E, Powell-Jackson T, Yip W. Doctor competence and the demand for healthcare: evidence from rural China. Health Econ 2017; 26: 1177–90. 27524208 10.1002/hec.3387

[123] New England Healthcare Institute Clinical care: a comprehensive analysis in support of system-wide improvements, 2 2008.

[124] WHO Antimicrobial resistance global report on surveillance. Geneva, Switzerland, 2014.

[125] Gibbons L, Belizán JM, Lauer JA, Betrán AP, Merialdi M, Althabe F. The global numbers and costs of additionally needed and unnecessary caesarean sections performed per year: overuse as a barrier to universal coverage. Geneva, Switzerland, 2010 http://www.who.int/healthsystems/topics/financing/healthreport/30C-sectioncosts.pdf (accessed Aug 14, 2018).

[126] Wagstaff A, Flores G, Smitz M-F, Hsu J, Chepynoga K, Eozenou P. Progress on impoverishing health spending in 122 countries: a retrospective observational study. Lancet Glob Health 2018; 6: e180–92. 29248366 10.1016/S2214-109X(17)30486-2

[127] Kruk ME, Mbaruku G, Rockers PC, Galea S. User fee exemptions are not enough: out-of-pocket payments for ‘free’ delivery services in rural Tanzania. Trop Med Int Health 2008; 13: 1442–51. 18983268 10.1111/j.1365-3156.2008.02173.x

[128] Wagstaff A, Flores G, Hsu J, et al Progress on catastrophic health spending in 133 countries: a retrospective observational study. Lancet Glob Health 2018; 6: e169–79. 29248367 10.1016/S2214-109X(17)30429-1

[129] Stenberg K, Hanssen O, Edejer TT-T, et al Financing transformative health systems towards achievement of the health Sustainable Development Goals: a model for projected resource needs in 67 low-income and middle-income countries. Lancet Glob Health 2017; 5: e875–87. 28728918 10.1016/S2214-109X(17)30263-2 PMC5554796

[130] Watkins D, Qi J, Horton S. Costing Universal Health Coverage: the DCP3 Model: DCP3 Working Paper Series. Working Paper # 20, 2017 http://dcp-3.org/sites/default/files/resources/20.%20Costs%20of%20UHC_Working%20Paper_Watkins%20_final%2013%20Nov_0.pdf (accessed Jan 14, 2018).

[131] Braveman P, Gruskin S. Defining equity in health. J Epidemiol Community Health 2003; 57: 254–58. 12646539 10.1136/jech.57.4.254 PMC1732430

[132] Hart JT. The inverse care law. Lancet 1971; 1: 405–12. 4100731 10.1016/s0140-6736(71)92410-x

[133] Lönnroth K, Jaramillo E, Williams BG, Dye C, Raviglione M. Drivers of tuberculosis epidemics: the role of risk factors and social determinants. Soc Sci Med 2009; 68: 2240–46. 19394122 10.1016/j.socscimed.2009.03.041

[134] Grintsova O, Maier W, Mielck A. Inequalities in health care among patients with type 2 diabetes by individual socio-economic status (SES) and regional deprivation: a systematic literature review. Int J Equity Health 2014; 13: 43. 24889694 10.1186/1475-9276-13-43 PMC4055912

[135] Watkins K. Leaving no-one behind: an equity agenda for the post-2015 goals 2013. https://www.odi.org/comment/7924-leaving-no-one-behind-equity-agenda-post-2015-goals (accessed Nov 4, 2017).

[136] Tangcharoensathien V, Mills A, Palu T. Accelerating health equity: the key role of universal health coverage in the Sustainable Development Goals. BMC Med 2015; 13: 101. 25925656 10.1186/s12916-015-0342-3 PMC4415234

[137] WHO Health in the 2030 Agenda for sustainable development. World Health Assembly resolution 6911. Geneva: World Health Organization, 2016.

[138] Tangcharoensathien V, Kanchanachitra C, Thomas R, Pfitzer JH, Whitney P. Addressing the health of vulnerable populations: a call for papers. Bull World Health Organ 2016; 94: 235.

[139] World Bank Inclusion Matters: The foundation for shared prosperity. Washington DC: The World Bank, 2013 https://openknowledge.worldbank.org/handle/10986/16195 (accessed Aug 14, 2018).

[140] Victora CG, Barros AJ, França GV, da Silva IC, Carvajal-Velez L, Amouzou A. The contribution of poor and rural populations to national trends in reproductive, maternal, newborn, and child health coverage: analyses of cross-sectional surveys from 64 countries. Lancet Glob Health 2017; 5: e402–07. 28238719 10.1016/S2214-109X(17)30077-3 PMC5565524

[141] Arsenault C, Harper S, Nandi A, Mendoza Rodríguez JM, Hansen PM, Johri M. Monitoring equity in vaccination coverage: a systematic analysis of demographic and health surveys from 45 Gavi-supported countries. Vaccine 2017; 35: 951–59. 28069359 10.1016/j.vaccine.2016.12.041

[142] Sharma J, Leslie HH, Kundu F, Kruk ME. Poor quality for poor women? Inequities in the quality of antenatal and delivery care in Kenya. PLoS One 2017; 12: e0171236. 28141840 10.1371/journal.pone.0171236 PMC5283741

[143] Das J, Mohpal A. Socioeconomic status and quality of care in rural India: new evidence from provider and household surveys. Health Aff (Millwood) 2016; 35: 1764–73. 27702947 10.1377/hlthaff.2016.0558

[144] Larson E, Hermosilla S, Kimweri A, Mbaruku GM, Kruk ME. Determinants of perceived quality of obstetric care in rural Tanzania: a cross-sectional study. BMC Health Serv Res 2014; 14: 483. 25326007 10.1186/1472-6963-14-483 PMC4283093

[145] Sofaer S, Firminger K. Patient perceptions of the quality of health services. Annu Rev Public Health 2005; 26: 513–59. 15760300 10.1146/annurev.publhealth.25.050503.153958

[146] Murphy A, Palafox B, O’Donnell O, et al Inequalities in the use of secondary prevention of cardiovascular disease by socioeconomic status: evidence from the PURE observational study. Lancet Glob Health 2018; 6: e292–301. 29433667 10.1016/S2214-109X(18)30031-7 PMC5905400

[147] Powell-Jackson T, Macleod D, Benova L, Lynch C, Campbell OM. The role of the private sector in the provision of antenatal care: a study of demographic and health surveys from 46 low- and middle-income countries. Trop Med Int Health 2015; 20: 230–39. 25358532 10.1111/tmi.12414

[148] Basu S, Andrews J, Kishore S, Panjabi R, Stuckler D. Comparative performance of private and public healthcare systems in low- and middle-income countries: a systematic review. PLoS Med 2012; 9: e1001244. 22723748 10.1371/journal.pmed.1001244 PMC3378609

[149] Jordan K, Marten R, Gureje O, Daelmans B, Kruk ME. Where is quality in health systems policy? A commentary on global policy documents. Lancet Glob Health (in press). 10.1016/S2214-109X(18)30375-9 PMC7473081 30322646

[150] Norheim OF. Ethical perspective: five unacceptable trade-offs on the path to universal health coverage. Int J Health Policy Manag 2015; 4: 711–14. 26673330 10.15171/ijhpm.2015.184 PMC4629695

[151] Persad GC, Emanuel EJ. The case for resource sensitivity: why it is ethical to provide cheaper, less effective treatments in global health. Hastings Cent Rep 2017; 47: 17–24. 28940341 10.1002/hast.764

[152] Jamison DT, Summers LH, Alleyne G, et al Global health 2035: a world converging within a generation. Lancet 2013; 382: 1898–955. 24309475 10.1016/S0140-6736(13)62105-4

[153] Gwatkin DR, Ergo A. Universal health coverage: friend or foe of health equity? Lancet 2011; 377: 2160–61. 21084113 10.1016/S0140-6736(10)62058-2

[154] Barros AJ, Victora CG, Cesar JA, Neumann NA, Bertoldi AD. Brazil: are health and nutrition programs reaching the neediest. Reaching the poor: with health, nutrition, and population services: what works, what doesn’t, and why Washington: The World Bank 2005: 281–306.

[155] Frenk J, González-Pier E, Gómez-Dantés O, Lezana MA, Knaul FM. Comprehensive reform to improve health system performance in Mexico. Lancet 2006; 368: 1524–34. 17071286 10.1016/S0140-6736(06)69564-0

[156] Miljeteig I, Onarheim KH, Defaye FB, et al Ethics capacity building in low-income countries: Ethiopia as a case study. Tidsskr Nor Laegeforen 2017; 137 DOI:10.4045/tidsskr.17.0759. 29181929

[157] Berwick DM. What ‘patient-centered’ should mean: confessions of an extremist. Health Aff (Millwood) 2009; 28: w555–65. 19454528 10.1377/hlthaff.28.4.w555

[158] Olivier de Sardan JP, Diarra A, Moha M. Travelling models and the challenge of pragmatic contexts and practical norms: the case of maternal health. Health Res Policy Syst 2017; 15 (suppl 1): 60. 28722553 10.1186/s12961-017-0213-9 PMC5516842

[159] Brinkerhoff D. Accountability and health systems: overview framework and strategies. Washington DC: Partners for Health Reform Plus, 2003.

[160] Govender C. The Deaths of 94 Mental Health-care Users in Gauteng, South Africa. Front Public Health 2017; 5: 126. 28620601 10.3389/fpubh.2017.00126 PMC5449499

[161] Brink S. Kenyan Woman Abused by Nurses during Childbirth wins Landmark Case. 2018 https://www.npr.org/sections/goatsandsoda/2018/04/10/600833683/kenyan-women-abused-by-nurses-during-childbirth-wins-landmark-case) (accessed Aug 14, 2018).

[162] Martin Hilber A, Blake C, Bohle LF, Bandali S, Agbon E, Hulton L. Strengthening accountability for improved maternal and newborn health: a mapping of studies in Sub-Saharan Africa. Int J Gynaecol Obstet 2016; 135: 345–57. 27802869 10.1016/j.ijgo.2016.09.008

[163] Van Belle S, Mayhew SH. Public accountability needs to be enforced -a case study of the governance arrangements and accountability practices in a rural health district in Ghana. BMC Health Serv Res 2016; 16: 568. 27729041 10.1186/s12913-016-1836-1 PMC5060000

[164] Buckley R. World Development Report 2004: Making services work for poor people-Overview. Washington, DC: The World Bank, 2003 http://documents.worldbank.org/curated/en/527371468166770790/World-Development-Report-2004-Making-services-work-for-poor-people-Overview (accessed Sept 10, 2017).

[165] National Department of Health Health Data Advisory and Co-ordination Committee (HDACC) Report. Republic of South Africa: National Department of Health, 2012 http://www.health.gov.za/index.php/2014–08–15–12–55–04/category/100–2012rp?download=187:health-data-advisory-and-co-ordination-committee-hdacc-report (accessed Aug 14, 2018).

[166] Hunter J, Chandran T, Asmall S, Tucker J-M, Ravhengani N, Mokgalagadi Y. The Ideal Clinic in South Africa: progress and challenges in implementation In: Padarath A, Barron P, eds. South African Health Review 2017. Durban: Health Systems Trust; 2017.

[167] World Bank Tanzania. Big results now for health project. Washington, DC: World Bank, 2014 http://documents.worldbank.org/curated/en/648601468112497753/Tanzania-Big-Results-Now-for-Health-Project (accessed April 26, 2018).

[168] World Bank Group Safety First: Improving access to quality health services in Kenya, expanding global knowledge on disease prevention. Washington, DC, 2016 http://pubdocs.worldbank.org/en/445551501768697125/KePSIE-Study-Brief.pdf (accessed May 2, 2018).

[169] Rwiyereka AK. Using Rwandan traditions to strengthen programme and policy implementation. Dev Pract 2014; 24: 686–92.

[170] Fujisawa R, Hewlett E, Nader C. Caring for Quality in Health: Lessons Learnt from 15 Reviews of Health Care Quality: OECD, 2017 https://www.oecd.org/els/health-systems/Caring-for-Quality-in-Health-Final-report.pdf (accessed June 2, 2017).

[171] National Advisory Group on the Safety of Patients in England A promise to learn—a commitment to act: Improving the Safety of Patients in England. England, 2013 https://www.gov.uk/government/uploads/system/uploads/attachment_data/file/226703/Berwick_Report.pdf (accessed March 17, 2017).

[172] National Quality Forum NQF’s strategic direction 2016–2019: lead, prioritize, and collaborate for better healthcare measurement. 2017 http://www.qualityforum.org/NQF_Strategic_Direction_2016–2019.aspx (accessed Nov 12, 2017).

[173] Health Data Collaborative Health data collaborative progress report 2016–2017: Health Data Collaborative, 2017 https://www.healthdatacollaborative.org/fileadmin/uploads/hdc/Documents/HealthDataCollaborative_Progress_Report_2016–2017.pdf (accessed Nov 11, 2017).

[174] World Bank Group, World Health Organization, Ministry of Finance, National Health and Family Planning Commission, Ministry of Human Resources and Social Security Deepening health reform in China, building high-quality and value-based service delivery. Washington, DC: World Bank Group, 2016 https://openknowledge.worldbank.org/bitstream/handle/10986/24720/HealthReformInChina.pdf (accessed Aug 14, 2018).

[175] Recommendations to OECD Ministers of Health from the high level reflection group on the future of health statistics: Strengthening the international comparison of health system performance through patient-reported indicators, 2017 http://www.oecd.org/health/health-systems/Recommendations-from-high-level-reflection-group-on-the-future-of-health-statistics.pdf (accessed Aug 14, 2018).

[176] La Vincente S, Aldaba B, Firth S, Kraft A, Jimenez-Soto E, Clark A. Supporting local planning and budgeting for maternal, neonatal and child health in the Philippines. Health Res Policy Syst 2013; 11: 3. 23343218 10.1186/1478-4505-11-3 PMC3557176

[177] Gates B. The next epidemic--lessons from Ebola. N Engl J Med 2015; 372: 1381–84. 25853741 10.1056/NEJMp1502918

[178] United Nations Development Program Sustainable Development Goals. Geneva: United Nations, 2015 http://www.un.org/sustainabledevelopment/sustainable-development-goals/ (accessed March 25, 2017).

[179] Martin GP, McKee L, Dixon-Woods M. Beyond metrics? Utilizing ‘soft intelligence’ for healthcare quality and safety. Soc Sci Med 2015; 142: 19–26. 26282705 10.1016/j.socscimed.2015.07.027 PMC4576210

[180] Wickremasinghe D, Hashmi IE, Schellenberg J, Avan BI. District decision-making for health in low-income settings: a systematic literature review. Health Policy Plan 2016; 31 (suppl 2): ii12–24. 27591202 10.1093/heapol/czv124 PMC5009221

[181] Mbondji PE, Kebede D, Soumbey-Alley EW, Zielinski C, Kouvividila W, Lusamba-Dikassa PS. Health information systems in Africa: descriptive analysis of data sources, information products and health statistics. J R Soc Med 2014; 107 (suppl): 34–45. 10.1177/0141076814531750 PMC4109358 24914127

[182] Kenya Ministry of Health Resource mapping for health information and monitoring and evaluation systems. Nairobi, Kenya: Ministry of Health, Republic of Kenya; USAID; Health Data Collaborative; Measure Evaluation PIMA, 2017 https://www.healthdatacollaborative.org/fileadmin/uploads/hdc/Documents/Country_documents/Kenya_HIS_Mapping_report_FINAL.pdf (accessed Nov 12, 2017).

[183] WHO Service Availability and Readiness Assessment (SARA) Reference Manual. Geneva: World Health Organization, 2013 http://www.who.int/healthinfo/systems/SARA_Reference_Manual_Full.pdf (accessed Feb 4, 2017).

[184] Gergen J, Josephson E, Coe M, Ski S, Madhavan S, Bauhoff S. Quality of care in performance-based financing: how it is incorporated in 32 programs across 28 countries. Glob Health Sci Pract 2017; 5: 90–107. 28298338 10.9745/GHSP-D-16-00239 PMC5493453

[185] Campbell H, El Arifeen S, Hazir T, et al Measuring coverage in MNCH: challenges in monitoring the proportion of young children with pneumonia who receive antibiotic treatment. PLoS Med 2013; 10: e1001421. 23667338 10.1371/journal.pmed.1001421 PMC3646212

[186] MEASURE Evaluation Routine health information systems: a curriculum on basic concepts and practices. Chapel Hill, North Carolina, USA: MEASURE Evaluation; 2017.

[187] AbouZahr C, Boerma T. Health information systems: the foundations of public health. Bull World Health Organ 2005; 83: 578–83. 16184276 PMC2626318

[188] WHO Global diffusion of eHealth: making universal health coverage achievable. Geneva: World Health Organization, 2016 http://www.who.int/goe/publications/global_diffusion/en/ (accessed Nov 14, 2017).

[189] Health Information Systems Programme DHIS 2 In Action. 2017 https://www.dhis2.org/inaction (accessed Jan 10, 2018).

[190] Akanbi MO, Ocheke AN, Agaba PA, et al Use of electronic health records in sub-Saharan Africa: progress and challenges. J Med Trop 2012; 14: 1–6. 25243111 PMC4167769

[191] Akhlaq A, McKinstry B, Muhammad KB, Sheikh A. Barriers and facilitators to health information exchange in low- and middle-income country settings: a systematic review. Health Policy Plan 2016; 31: 1310–25. 27185528 10.1093/heapol/czw056

[192] Dare AJ, Ng-Kamstra JS, Patra J, et al, and the Million Death Study Collaborators Deaths from acute abdominal conditions and geographical access to surgical care in India: a nationally representative spatial analysis. Lancet Glob Health 2015; 3: e646–53. 26278186 10.1016/S2214-109X(15)00079-0

[193] Boerma T, AbouZahr C, Evans D, Evans T. Monitoring intervention coverage in the context of universal health coverage. PLoS Med 2014; 11: e1001728. 25243586 10.1371/journal.pmed.1001728 PMC4171108

[194] Ng M, Fullman N, Dieleman JL, Flaxman AD, Murray CJL, Lim SS. Effective coverage: a metric for monitoring Universal Health Coverage. PLoS Med 2014; 11: e1001730. 25243780 10.1371/journal.pmed.1001730 PMC4171091

[195] Cazabon D, Alsdurf H, Satyanarayana S, et al Quality of tuberculosis care in high burden countries: the urgent need to address gaps in the care cascade. Int J Infect Dis 2017; 56: 111–16. 27794468 10.1016/j.ijid.2016.10.016 PMC5346036

[196] Mulley A, Coulter A, Wolpert M, Richards T, Abbasi K. New approaches to measurement and management for high integrity health systems. BMJ 2017; 356: j1401. 28360140 10.1136/bmj.j1401

[197] Nyblade L, Stangl A, Weiss E, Ashburn K. Combating HIV stigma in health care settings: what works? J Int AIDS Soc 2009; 12: 15. 19660113 10.1186/1758-2652-12-15 PMC2731724

[198] Leisher SH, Sprockett A, Longfield K, Montagu D, eds. Quality measurement in family planning: past, present, future: papers from the Bellagio meeting on family planning quality, October 2015. Oakland, CA: Metrics for Management, 2016 http://m4mgmt.org/wp-content/uploads/2017/07/Bellagio-book-web.pdf (accessed Aug 14, 2018).

[199] International Consortium for Health Outcomes Measurement Hypertension in low- and middle-income countries: data collection reference guide. Cambridge, MA, 2017 http://www.ichom.org/download/hypertension-in-low-and-middle-income-countries/ (accessed Nov 15, 2017).

[200] Okunade O, Arora J, Haverhals A, Niessen L. Collaborating for Value: the Santeon Hospitals in the Netherlands. Cambridge, MA: ICHOM, 2017 http://www.ichom.org/wp-content/uploads/2013/10/Santeon_Case_Study_Final.pdf (accessed Nov 15, 2017).

[201] Batbaatar E, Dorjdagva J, Luvsannyam A, Amenta P. Conceptualisation of patient satisfaction: a systematic narrative literature review. Perspect Public Health 2015; 135: 243–50. 26187638 10.1177/1757913915594196

[202] Batbaatar E, Dorjdagva J, Luvsannyam A, Savino MM, Amenta P. Determinants of patient satisfaction: a systematic review. Perspect Public Health 2017; 137: 89–101. 27004489 10.1177/1757913916634136

[203] Martin L, Nelson E. Whole system measures 2.0: a compass for health system leaders. Cambridge, Massachusetts: Institute for Healthcare Improvement; 2016.

[204] English M, Mwaniki P, Julius T, et al Hospital mortality—a neglected but rich source of information supporting the transition to higher quality health systems in low and middle income countries. BMC Med 2018; 16: 32. 29495961 10.1186/s12916-018-1024-8 PMC5833062

[205] Thomas J, Ayieko P, Ogero M, et al, and the Clinical Information Network Blood transfusion delay and outcome in county hospitals in Kenya. Am J Trop Med Hyg 2017; 96: 511–17. 27920394 10.4269/ajtmh.16-0735 PMC5303061

[206] Maluka S, Kamuzora P, San Sebastiån M, et al Decentralized health care priority-setting in Tanzania: evaluating against the accountability for reasonableness framework. Soc Sci Med 2010; 71: 751–59. 20554365 10.1016/j.socscimed.2010.04.035

[207] Barasa EW, Cleary S, Molyneux S, English M. Setting healthcare priorities: a description and evaluation of the budgeting and planning process in county hospitals in Kenya. Health Policy Plan 2017; 32: 329–37. 27679522 10.1093/heapol/czw132 PMC5362066

[208] Boerma T, Victora C, Abouzahr C. Monitoring country progress and achievements by making global predictions: is the tail wagging the dog? Lancet 2018; published online April 13. 10.1016/S0140-6736(18)30586-5. 29661480

[209] Walshe K, Shortell SM. When things go wrong: how health care organizations deal with major failures. Health Aff (Millwood) 2004; 23: 103–11. 10.1377/hlthaff.23.3.103 15160808

[210] Rao S. Hospital geocodes for Karnataka, India. 2016.

[211] Bhattacharyya S, Berhanu D, Taddesse N, et al District decision-making for health in low-income settings: a case study of the potential of public and private sector data in India and Ethiopia. Health Policy Plan 2016; 31 (suppl 2): ii25–34. 27591203 10.1093/heapol/czw017 PMC5009222

[212] WHO A handbook for national quality policy and strategy: a practical approach for developing policy and strategy to improve quality of care. Geneva: World Health Organization, 2018.

[213] Hoque DME, Kumari V, Hoque M, Ruseckaite R, Romero L, Evans SM. Impact of clinical registries on quality of patient care and clinical outcomes: A systematic review. PLoS One 2017; 12: e0183667. 28886607 10.1371/journal.pone.0183667 PMC5591016

[214] Andhra Pradesh Department of Health Medical and Family Welfare Bio Metric Attendance. 2018 http://hmfw.ap.gov.in/bio-metric.aspx (accessed Feb 1, 2018).

[215] Kenya National Bureau of Statistics Kenya Data Portal. 2018 http://kenya.opendataforafrica.org/ (accessed Feb 1, 2018).

[216] Evans AM, Campos A. Open government initiatives: challenges of citizen participation. J Policy Anal Manage 2012; 32: 172–85.

[217] Werner RM, Asch DA. The unintended consequences of publicly reporting quality information. JAMA 2005; 293: 1239–44. 15755946 10.1001/jama.293.10.1239

[218] Amabile TM, Kramer SJ. The power of small wins. Harv Bus Rev 2011; 89: 70–80.

[219] Dixon-Woods M, Martin G. Does quality improvement improve quality? Future Hosp J 2016; 3: 191–94. 31098223 10.7861/futurehosp.3-3-191 PMC6465806

[220] Dixon-Woods M, Pronovost PJ. Patient safety and the problem of many hands. BMJ Qual Saf 2016; 25: 485–88. 10.1136/bmjqs-2016-005232 PMC4959572 26912578

[221] Lipsitz LA. Understanding health care as a complex system: the foundation for unintended consequences. JAMA 2012; 308: 243–44. 22797640 10.1001/jama.2012.7551 PMC3511782

[222] Adam T, de Savigny D. Systems thinking for strengthening health systems in LMICs: need for a paradigm shift. Health Policy Plan 2012; 27 (suppl 4): iv1–3. 23014149 10.1093/heapol/czs084

[223] De Savigny D, Adam T. Systems thinking for health systems strengthening. Geneva World Health Organization; 2009.

[224] Thaler RH. Nudge: improving decisions about health, wealth, and happiness. New Haven; 2008.

[225] WHO Health Systems Governance for Universal Health Coverage: Action Plan. Geneva, Switzerland, 2014 http://www.who.int/universal_health_coverage/plan_action-hsgov_uhc/en/ (accessed Jan 23, 2018).

[226] Siddiqi S, Masud TI, Nishtar S, et al Framework for assessing governance of the health system in developing countries: gateway to good governance. Health Policy 2009; 90: 13–25. 18838188 10.1016/j.healthpol.2008.08.005

[227] WHO Quality of care: a process for making strategic choices in health systems. Geneva: World Health Organization, 2006 http://www.who.int/management/quality/assurance/QualityCare_B.Def.pdf (accessed Aug 14, 2018).

[228] Doubova SV, García-Saiso S, Pérez-Cuevas R, et al Quality governance in a pluralistic health system: Mexican experience and challenges. Lancet Glob Health 2018; published online Sept 5. 10.1016/S2214-109X(18)30321-8. 30196095

[229] Stroh DP. Systems thinking for social change: a practical guide to solving complex problems, avoiding unintended consequences, and achieving lasting results. White River Junction, Vermont Chelsea Green Publishing; 2015.

[230] Edmondson AC, Harvey J-F. Cross-boundary teaming for innovation: Integrating research on teams and knowledge in organizations. Hum Resour Manage Rev 2017; DOI:10.1016/j.hrmr.2017.03.002.

[231] Grépin KA. HIV donor funding has both boosted and curbed the delivery of different non-HIV health services in sub-Saharan Africa. Health Aff (Millwood) 2012; 31: 1406–14. 22778329 10.1377/hlthaff.2012.0279

[232] Wilson N. Can Disease-Specific Funding Harm Health? in the Shadow of HIV/AIDS Service Expansion. Demography 2015; 52: 1671–700. 26370281 10.1007/s13524-015-0427-9

[233] Topp SM, Chipukuma JM. How did rapid scale-up of HIV services impact on workplace and interpersonal trust in Zambian primary health centres: a case-based health systems analysis. BMJ Glob Health 2016; 1: e000179. 10.1136/bmjgh-2016-000179 PMC5321392 28588985

[234] WHO Towards better leadership and management in health: report on an international consultation on strengthening leadership and management in low-income countries. Geneva, Switerland, 2007.

[235] Bradley EH, Taylor LA, Cuellar CJ. Management matters: a leverage point for health systems strengthening in global health. Int J Health Policy Manag 2015; 4: 411–15. 26188805 10.15171/ijhpm.2015.101 PMC4493581

[236] Lega F, Prenestini A, Spurgeon P. Is management essential to improving the performance and sustainability of health care systems and organizations? A systematic review and a roadmap for future studies. Value Health 2013; 16 (suppl): S46–51. 23317645 10.1016/j.jval.2012.10.004

[237] Mabuchi S, Sesan T, Bennett SC. Pathways to high and low performance: factors differentiating primary care facilities under performance-based financing in Nigeria. Health Policy Plan 2018; 33: 41–58. 29077844 10.1093/heapol/czx146 PMC5886213

[238] Bradley EH, Byam P, Alpern R, et al A systems approach to improving rural care in Ethiopia. PLoS One 2012; 7: e35042. 22558113 10.1371/journal.pone.0035042 PMC3338815

[239] Kebede S, Mantopoulos J, Ramanadhan S, et al Educating leaders in hospital management: a pre-post study in Ethiopian hospitals. Glob Public Health 2012; 7: 164–74. 21259143 10.1080/17441692.2010.542171

[240] Umble KE, Brooks J, Lowman A, et al Management training in Vietnam’s National Tuberculosis Program: an impact evaluation. Int J Tuberc Lung Dis 2009; 13: 238–46. 19146754

[241] Fryatt R, Bennett S, Soucat A. Health sector governance: should we be investing more? BMJ Glob Health 2017; 2: e000343. 10.1136/bmjgh-2017-000343 PMC5717939 29225938

[242] El-Jardali F, Akl EA, Fadlallah R, et al Interventions to combat or prevent drug counterfeiting: a systematic review. BMJ Open 2015; 5: e006290. 10.1136/bmjopen-2014-006290 PMC4368988 25787989

[243] Akhtar A. Health care regulation in low-and middle income countries: a review of the literature. Health Policy and Health Finance Knowledge Hub: Working Paper Series. 2011; (14).

[244] WHO Strengthening the capacity of governments to constructively engage the private sector in providing essential health-care services: Report by the Secretariat. Geneva, 2010 http://apps.who.int/gb/ebwha/pdf_files/WHA63/A63_25-en.pdf (accessed July 19, 2017).

[245] Naylor CD, Gerace R, Redelmeier DA. Maintaining physician competence and professionalism: Canada’s fine balance. JAMA 2015; 313: 1825–26. 25965227 10.1001/jama.2015.3705

[246] Dixon-Woods M, Yeung K, Bosk CL. Why is U.K. medicine no longer a self-regulating profession? The role of scandals involving “bad apple” doctors. Soc Sci Med 2011; 73: 1452–59. 21975027 10.1016/j.socscimed.2011.08.031

[247] Bauchner H, Fontanarosa PB, Thompson AE. Professionalism, governance, and self-regulation of medicine. JAMA 2015; 313: 1831–36. 25965230 10.1001/jama.2015.4569

[248] Del Rosario SS. PMA legacy. Philippine Medical Association https://www.philippinemedicalassociation.org/pma-legacy/ (accessed April 25, 2018).

[249] Potts H. Partiticipation and the Right to the Highest Attainable Standard of Health: University of Essex: Human Rights Centre, 2008 http://repository.essex.ac.uk/9714/1/participation-right-highest-attainable-standard-health.pdf (accessed Aug 14, 2018).

[250] Howard-Grabman L, Miltenburg AS, Marston C, Portela A. Factors affecting effective community participation in maternal and newborn health programme planning, implementation and quality of care interventions. BMC Pregnancy Childbirth 2017; 17: 268. 28854886 10.1186/s12884-017-1443-0 PMC5577661

[251] Wallerstein N. What is the evidence on effectiveness of empowerment to improve health? Copenhagen: WHO Regional Office for Europe, 2006 http://www.euro.who.int/Document/E88086.pdf (accessed March 24, 2018).

[252] Tangcharoensathien V, Witthayapipopsakul W, Panichkriangkrai W, Patcharanarumol W, Mills A. Health systems development in Thailand: a solid platform for successful implementation of universal health coverage. Lancet 2018; 391: 1205–23. 29397200 10.1016/S0140-6736(18)30198-3

[253] London L, Schneider H. Globalisation and health inequalities: can a human rights paradigm create space for civil society action? Soc Sci Med 2012; 74: 6–13. 21511377 10.1016/j.socscimed.2011.03.022

[254] Ray S, Madzimbamuto F, Fonn S. Activism: working to reduce maternal mortality through civil society and health professional alliances in sub-Saharan Africa. Reprod Health Matters 2012; 20: 40–49. 22789081 10.1016/S0968-8080(12)39617-1

[255] Rowe AK, Rowe SY, Peters DH, Holloway KA, Chalker J, Ross-Degnan D. A systematic review of the effectiveness of strategies to improve health care provider practices in low-income and middle-income countries. Lancet Glob Health. (in press) 10.1016/S2214-109X(18)30398-X PMC6185992 30309799

[256] Greenfield D, Braithwaite J. Health sector accreditation research: a systematic review. Int J Qual Health Care 2008; 20: 172–83. 18339666 10.1093/intqhc/mzn005

[257] Barbazza E, Tello JE. A review of health governance: definitions, dimensions and tools to govern. Health Policy 2014; 116: 1–11. 24485914 10.1016/j.healthpol.2014.01.007

[258] Langley GJ, Moen R, Nolan K, Nolan T, Norman C, Provost L. The improvement guide: a practical approach to enhancing organizational performance. 2nd edn. San Francisco, California Jossey-Bass Publishers; 2009.

[259] Quao NSA, Bonney J, Forson PK, Oduro G. Overcrowding in a low resource emergency setting in west Africa: perceptions by health workers in the accident and emergency center, Komfo Anokye Teaching Hospital (Kath) Kumasi, Ghana. Prehosp Disaster Med 2017; 32: S33–34.

[260] El-Sadr WM, Harripersaud K, Rabkin M. Reaching global HIV/AIDS goals: What got us here, won’t get us there. PLoS Med 2017; 14: e1002421. 29112691 10.1371/journal.pmed.1002421 PMC5675304

[261] Duncombe C, Rosenblum S, Hellmann N, et al Reframing HIV care: putting people at the centre of antiretroviral delivery. Trop Med Int Health 2015; 20: 430–47. 25583302 10.1111/tmi.12460 PMC4670701

[262] Kredo T, Ford N, Adeniyi FB, Garner P. Decentralising HIV treatment in lower- and middle-income countries. Cochrane Database Syst Rev 2013; 6: CD009987. 10.1002/14651858.CD009987.pub2 PMC10009870 23807693

[263] Holmes KK, Bertozzi S, Bloom BR, Jha P. Disease Control Priorities, Third Edition: Volume 6. Major Infectious Diseases. Washington, DC World Bank; 2017. 30212102

[264] Bitton A, Ratcliffe HL, Veillard JH, et al Primary health care as a foundation for strengthening health systems in low- and middle-income countries. J Gen Intern Med 2017; 32: 566–71. 27943038 10.1007/s11606-016-3898-5 PMC5400754

[265] Duffy S, Lee TH. In-Person Health Care as Option B. N Engl J Med 2018; 378: 104–06. 29320653 10.1056/NEJMp1710735

[266] Lee A, Cousens S, Darmstadt G, et al Care during labor and birth for the prevention of intrapartum-related neonatal deaths: a systematic review and Delphi estimation of mortality effect. BMC Public Health 2011; 11 (suppl 3): S10. 10.1186/1471-2458-11-S3-S10 PMC3231883 21501427

[267] Kruk ME, Leslie HH, Verguet S, Mbaruku GM, Adanu RMK, Langer A. Quality of basic maternal care functions in health facilities of five African countries: an analysis of national health system surveys. Lancet Glob Health 2016; 4: e845–55. 27670090 10.1016/S2214-109X(16)30180-2

[268] Elmusharaf K, Byrne E, AbuAgla A, et al Patterns and determinants of pathways to reach comprehensive emergency obstetric and neonatal care (CEmONC) in South Sudan: qualitative diagrammatic pathway analysis. BMC Pregnancy Childbirth 2017; 17: 278. 28851308 10.1186/s12884-017-1463-9 PMC5576292

[269] Straneo M, Hanson C, Fogliati P, Mbaruku GM. Minimum obstetric volume in low-income countries. Lancet 2017; 389: 698. 10.1016/S0140-6736(17)30342-2 28229874

[270] Goldenberg RL, McClure EM. Improving birth outcomes in low- and middle-income countries. N Engl J Med 2017; 377: 2387–88. 29236634 10.1056/NEJMe1713831

[271] Hofman JJ, Dzimadzi C, Lungu K, Ratsma EY, Hussein J. Motorcycle ambulances for referral of obstetric emergencies in rural Malawi: do they reduce delay and what do they cost? Int J Gynaecol Obstet 2008; 102: 191–97. 18555998 10.1016/j.ijgo.2008.04.001

[272] BellowsB, ConlonCM, HiggsE, et al A taxonomy and results from a comprehensive review of 28 maternal health voucher programmes. J Health Popul Nutr 2013; 31 (suppl 2): 106–28.24992806

[273] Gorry C. Cuban maternity homes: a model to address at-risk pregnancy. MEDICC Rev 2011; 13: 12–15. 21778953 10.37757/MR2011V13.N3.4

[274] Ouma PO, Maina J, Thuranira PN, et al Access to emergency hospital care provided by the public sector in sub-Saharan Africa in 2015: a geocoded inventory and spatial analysis. Lancet Glob Health 2018; 6: e342–50. 29396220 10.1016/S2214-109X(17)30488-6 PMC5809715

[275] Bailey PE, Keyes EB, Parker C, Abdullah M, Kebede H, Freedman L. Using a GIS to model interventions to strengthen the emergency referral system for maternal and newborn health in Ethiopia. Int J Gynaecol Obstet 2011; 115: 300–09. 21982854 10.1016/j.ijgo.2011.09.004

[276] Munabi-Babigumira S, Glenton C, Lewin S, Fretheim A, Nabudere H. Factors that influence the provision of intrapartum and postnatal care by skilled birth attendants in low- and middle-income countries: a qualitative evidence synthesis. Cochrane Database Syst Rev 2017; 11: CD011558. 29148566 10.1002/14651858.CD011558.pub2 PMC5721625

[277] Agyepong IA, Sewankambo N, Binagwaho A, et al The path to longer and healthier lives for all Africans by 2030: the *Lancet* Commission on the future of health in sub-Saharan Africa. Lancet 2018; 390: 2803–59. 28917958 10.1016/S0140-6736(17)31509-X

[278] Mullan F, Frehywot S, Omaswa F, et al Medical schools in sub-Saharan Africa. Lancet 2011; 377: 1113–21. 21074256 10.1016/S0140-6736(10)61961-7

[279] Global Health Workforce Alliance, WHO A universal truth: no health without a workforce. Geneva: World Health Organization, 2014 http://www.who.int/workforcealliance/knowledge/resources/GHWA-a_universal_truth_report.pdf?ua=1 (accessed May 5, 2017).

[280] Leslie HH, Gage A, Nsona H, Hirschhorn LR, Kruk ME. Training and supervision did not meaningfully improve quality of care for pregnant women or sick children in sub-saharan Africa. Health Aff (Millwood) 2016; 35: 1716–24. 27605655 10.1377/hlthaff.2016.0261

[281] Frenk J, Chen L, Bhutta ZA, et al Health professionals for a new century: transforming education to strengthen health systems in an interdependent world. Lancet 2010; 376: 1923–58. 21112623 10.1016/S0140-6736(10)61854-5

[282] Kaaya EE, Macfarlane SB, Mkony CA, et al Educating enough competent health professionals: advancing educational innovation at Muhimbili University of Health and Allied Sciences, Tanzania. PLoS Med 2012; 9: e1001284. 22904688 10.1371/journal.pmed.1001284 PMC3419186

[283] Lozano R, Soliz P, Gakidou E, et al Benchmarking of performance of Mexican states with effective coverage. Lancet 2006; 368: 1729–41. 17098091 10.1016/S0140-6736(06)69566-4

[284] Thornton RLJ, Powe NR, Roter D, Cooper LA. Patient-physician social concordance, medical visit communication and patients’ perceptions of health care quality. Patient Educ Couns 2011; 85: e201–08. 21840150 10.1016/j.pec.2011.07.015 PMC3217162

[285] Cooper LA, Roter DL, Johnson RL, Ford DE, Steinwachs DM, Powe NR. Patient-centered communication, ratings of care, and concordance of patient and physician race. Ann Intern Med 2003; 139: 907–15. 14644893 10.7326/0003-4819-139-11-200312020-00009

[286] El Koussa M, Atun R, Bowser D, Kruk ME. Factors influencing physicians’ choice of workplace: systematic review of drivers of attrition and policy interventions to address them. J Glob Health 2016; 6: 020403. 27648254 10.7189/jogh.06.020403 PMC5017032

[287] Selamu M, Thornicroft G, Fekadu A, Hanlon C. Conceptualisation of job-related wellbeing, stress and burnout among healthcare workers in rural Ethiopia: a qualitative study. BMC Health Serv Res 2017; 17: 412. 28629360 10.1186/s12913-017-2370-5 PMC5477383

[288] Kazmi R, Amjad S, Khan D. Occupational stress and its effect on job performance. A case study of medical house officers of district Abbottabad. J Ayub Med Coll Abbottabad 2008; 20: 135–39. 19610539

[289] Aloulou J, Damak R, Masmoudi F, Sidhom O, Amami O. [Burn out in health care providers: a Tunisian study about 142 nurses]. Tunis Med 2013; 91: 44–49. 23404597

[290] Kumar S. Burnout and Doctors: Prevalence, prevention and intervention. Healthcare (Basel) 2016; 4: E37. 27417625 10.3390/healthcare4030037 PMC5041038

[291] Willis-Shattuck M, Bidwell P, Thomas S, Wyness L, Blaauw D, Ditlopo P. Motivation and retention of health workers in developing countries: a systematic review. BMC Health Serv Res 2008; 8: 247. 19055827 10.1186/1472-6963-8-247 PMC2612662

[292] Linzer M, Poplau S, Grossman E, et al A cluster randomized trial of interventions to improve work conditions and clinician burnout in primary care: results from the healthy work place (HWP) study. J Gen Intern Med 2015; 30: 1105–11. 25724571 10.1007/s11606-015-3235-4 PMC4510236

[293] WHO Global strategy on human resources for health: workforce 2030. Geneva: World Health Organization, 2016 http://apps.who.int/iris/bitstream/10665/250368/1/9789241511131-eng.pdf (accessed June 2, 2017).

[294] Perlo J, Balik B, Swensen S, Kabcenell A, Landsman J, Feeley D. IHI Framework for Improving Joy in Work White Paper. Cambridge, Massachusetts: Institute for Healthcare Improvement, 2017.

[295] The World Health Report 2000 Health systems: improving performance. Geneva World Health Organization; 2001. 11910962

[296] UN Committee on Economic, Social, and Cultural Rights Substantive issues arising in the implementation of the International Covenant on Economic, Social and Cultural Rights. Geneva: United Nations Economic and Social Council, 2000 https://digitallibrary.un.org/record/628545?ln=en (accessed April 18, 2017).

[297] Hibbard JH, Greene J. What the evidence shows about patient activation: better health outcomes and care experiences; fewer data on costs. Health Aff (Millwood) 2013; 32: 207–14. 23381511 10.1377/hlthaff.2012.1061

[298] Cohen J, Golub G, Kruk ME, McConnell M. Do active patients seek higher quality prenatal care?: A panel data analysis from Nairobi, Kenya. Prev Med 2016; 92: 74–81. 27667338 10.1016/j.ypmed.2016.09.014 PMC5100690

[299] Prost A, Colbourn T, Seward N, et al Women’s groups practising participatory learning and action to improve maternal and newborn health in low-resource settings: a systematic review and meta-analysis. Lancet 2013; 381: 1736–46. 23683640 10.1016/S0140-6736(13)60685-6 PMC3797417

[300] Molina E, Carella L, Pacheco A, Cruces G, Gasparini L. Community monitoring interventions to curb corruption and increase access and quality in service delivery: a systematic review. J Dev Effect 2017; 9: 462–99.

[301] Björkman M, Svensson J. Power to the People: Evidence from a Randomized Field Experiment on Community-Based Monitoring in Uganda. Q J Econ 2009; 124: 735–69.

[302] Nyqvist MB. Walque Dd, Svensson J. Information is power: experimental evidence on the long-run impact of community based monitoring, 2014 http://hdl.handle.net/10986/20364 (accessed May 1, 2017).

[303] Pandey P, Sehgal AR, Riboud M, Levine D, Goyal M. Informing resource-poor populations and the delivery of entitled health and social services in rural India: a cluster randomized controlled trial. JAMA 2007; 298: 1867–75. 17954538 10.1001/jama.298.16.1867

[304] Gullo S, Galavotti C, Altman L. A review of CARE’s Community Score Card experience and evidence. Health Policy Plan 2016; 31: 1467–78. 27190223 10.1093/heapol/czw064 PMC5091339

[305] Centola D, Macy M. Complex Contagions and the weakness of long ties 1. Am J Sociol 2007; 113: 702–34.

[306] Leonard KL, Adelman SW, Essam T. Idle chatter or learning? Evidence of social learning about clinicians and the health system from rural Tanzania. Soc Sci Med 2009; 69: 183–90. 19501941 10.1016/j.socscimed.2009.05.020

[307] WHO Developing a national health financing strategy: a reference guide. Geneva, Switzerland: WHO, 2017 http://apps.who.int/iris/bitstream/10665/254757/1/9789241512107-eng.pdf?ua=1 (accessed Aug 14, 2018).

[308] WHO Strategic purchasing for universal health coverage: unlocking the potential. Global meeting summary and key messages. Geneva Geneva: World Health Organization, 2017 http://www.who.int/iris/handle/10665/254757 (accessed Feb 1, 2018).

[309] Musgrove P. Financial and other rewards for good performance or results: a guided tour of concepts and terms and a short glossary. Washington, DC: The World Bank, 2011 https://www.rbfhealth.org/sites/rbf/files/RBFglossarylongrevised_0.pdf (accessed Aug 14, 2018).

[310] Josephson E, Gergen J, Coe M, Ski S, Madhavan S, Bauhoff S. How do performance-based financing programmes measure quality of care? A descriptive analysis of 68 quality checklists from 28 low- and middle-income countries. Health Policy Plan 2017; 32: 1120–26. 28549142 10.1093/heapol/czx053 PMC5886109

[311] Meessen B, Soucat A, Sekabaraga C. Performance-based financing: just a donor fad or a catalyst towards comprehensive health-care reform? Bull World Health Organ 2011; 89: 153–56. 21346927 10.2471/BLT.10.077339 PMC3040374

[312] Health Results Innovation Trust Fund Achieving results for women’s and children’s health: progress report 2015: World Bank, 2015 https://www.rbfhealth.org/publication/achieving-results-women%E2%80%99s-and-childrens-health-2015-progress-report (accessed Jan 11, 2018).

[313] Health Results Innovation Trust Fund Learning agenda for results-based financing in the health sector the health results innovation trust fund learning strategy: World Bank, 2016 https://www.rbfhealth.org/sites/rbf/files/The%20Health%20Results%20Innovation%20Trust%20Fund%20Learning%20Strategy.pdf (accessed March 26, 2017).

[314] Binyaruka P, Patouillard E, Powell-Jackson T, Greco G, Maestad O, Borghi J. Effect of paying for performance on utilisation, quality, and user costs of health services in Tanzania: a controlled before and after study. PLoS One 2015; 10: e0135013. 26317510 10.1371/journal.pone.0135013 PMC4552688

[315] Yip W, Powell-Jackson T, Chen W, et al Capitation combined with pay-for-performance improves antibiotic prescribing practices in rural China. Health Aff (Millwood) 2014; 33: 502–10. 24572187 10.1377/hlthaff.2013.0702

[316] de Walque D, Robyn PJ, Saidou H, Sorgho G, Steenland M. Looking into the performance-based financing black box: evidence from an impact evaluation in the health sector in Cameroon. Washington DC: The World Bank, 2017 http://documents.worldbank.org/curated/en/834601502391015068/Looking-into-the-performance-based-financing-black-box-evidence-from-an-impact-evaluation-in-the-health-sector-in-Cameroon (accessed Nov 5, 2017). 10.1093/heapol/czab002 PMC12147217 33963406

[317] Friedman J, Qamruddin JN, Chansa C, Das AK. Impact Evaluation of Zambia’s Health Results-Based Financing Pilot Project. Washington DC: World Bank, 2016 http://documents.worldbank.org/curated/en/798081509456632349/Impact-evaluation-of-Zambia-s-health-results-based-financing-pilot-project (accessed May 14, 2017).

[318] Borghi J, Little R, Binyaruka P, Patouillard E, Kuwawenaruwa A. In Tanzania, the many costs of pay-for-performance leave open to debate whether the strategy is cost-effective. Health Aff (Millwood) 2015; 34: 406–14. 25732490 10.1377/hlthaff.2014.0608

[319] Chimhutu V, Lindkvist I, Lange S. When incentives work too well: locally implemented pay for performance (P4P) and adverse sanctions towards home birth in Tanzania—a qualitative study. BMC Health Serv Res 2014; 14: 23. 24438556 10.1186/1472-6963-14-23 PMC3897973

[320] Conrad DA, Perry L. Quality-based financial incentives in health care: can we improve quality by paying for it? Annu Rev Public Health 2009; 30: 357–71. 19296779 10.1146/annurev.publhealth.031308.100243

[321] Mohanan M, Giardili S, Das V, et al Evaluation of a social franchising and telemedicine programme and the care provided for childhood diarrhoea and pneumonia, India. 2017. 10.2471/BLT.16.179556 PMC5418816 28479635

[322] Kibria A, Mancher M, McCoy MA, Graham RP, Garber AM, Newhouse JP. Variation in health care spending: target decision making, not geography. Washington, DC: The National Academies Press, 2013. 24851301

[323] Ekman B, Pathmanathan I, Liljestrand J. Integrating health interventions for women, newborn babies, and children: a framework for action. Lancet 2008; 372: 990–1000. 18790321 10.1016/S0140-6736(08)61408-7

[324] Schouten LM, Hulscher ME, van Everdingen JJ, Huijsman R, Grol RP. Evidence for the impact of quality improvement collaboratives: systematic review. BMJ 2008; 336: 1491–94. 18577559 10.1136/bmj.39570.749884.BE PMC2440907

[325] Wells S, Tamir O, Gray J, Naidoo D, Bekhit M, Goldmann D. Are quality improvement collaboratives effective? A systematic review. BMJ Qual Saf 2018; 27: 226–40. 10.1136/bmjqs-2017-006926 29055899

[326] Magge H, Chilengi R, Jackson EF, Wagenaar BH, Kante AM, the AHI PHIT Partnership Collaborative Tackling the hard problems: implementation experience and lessons learned in newborn health from the African Health Initiative. BMC Health Serv Res 2017; 17 (suppl 3): 829. 29297352 10.1186/s12913-017-2659-4 PMC5763287

[327] WHO The Innov8 approach for reviewing national health programmes to leave no one behind. 2016 http://apps.who.int/iris/bitstream/10665/250442/1/9789241511391-eng.pdf?ua=1 (accessed Aug 14, 2018). 10.1080/16549716.2018.1423744 PMC5912432 29569529

